# Ringing the Changes: Effects of Heterocyclic Ring Size on Stereoselectivity in [(η^5^-C_5_Me_5_)RhCl], [(η^5^-C_5_Me_5_)IrCl] and [Ru(η^6^-cymene)Cl] Complexes of Chiral 3-Amino-1-Azacycles

**DOI:** 10.3390/molecules29194659

**Published:** 2024-09-30

**Authors:** Vladimir Y. Vladimirov, Matheo Charrier-Chardin, Benson M. Kariuki, Benjamin D. Ward, Paul D. Newman

**Affiliations:** School of Chemistry, Cardiff University, Cardiff CF10 3AT, UK; vladimirovvy@cardiff.ac.uk (V.Y.V.); matheo.charrierchardin@gmail.com (M.C.-C.); wardbd@cardiff.ac.uk (B.D.W.)

**Keywords:** diastereoselectivity, azacycles, transfer hydrogenation, diamines

## Abstract

Ring size-dependent diastereoselective coordination of unsymmetrical diamines containing one azacyclic nitrogen and one exocyclic nitrogen to [(η^5^-C_5_Me_5_)MCl]^+^ cores where M = Rh, Ir and [Ru(η^6^-cymene)Cl]^+^ is reported herein. Total stereoselectivity was observed with the six- and seven-membered azacycles, whereas the five-derivative proved poorly selective. All complexes were active for transfer hydrogenation but showed no enantioselectivity with prochiral ketones.

## 1. Introduction

Stereogenic-at-metal complexes are useful catalysts in asymmetric photo, outer-sphere (Hydrogen-bonding) and Lewis acid catalysis [1,2,3,4,5,6,7,8,9,10]. Piano-stool complexes of Rh, Ir and Ru figure frequently within these fields and examples include those that are stereogenic-at-metal only [8,11,12,13], as well as diastereomeric complexes resulting from the use of a prescribed chiral ligand(s) [11,12,13,14,15,16,17,18,19]. These latter systems often perform extremely well in asymmetric transformations and are synthetically judicious, as complete control of the stereogenic metal centre is readily achieved through the astute choice of chiral ligand(s).

**Figure 1:**
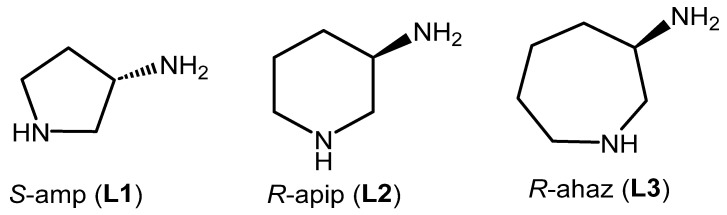
Structure of the aminoazacyclic ligands.

In response to Noyori’s seminal work [20], mono-tosylated diamine ligands have proven to be popular for asymmetric transformations using half-sandwich complexes of Rh, Ir and Ru [21,22,23]. Although employed far less, asymmetric transfer hydrogenation catalysts bearing non-tosylated diamines have been reported [24]. To our surprise, we were unable to find any examples containing the readily available 3-amino-1-azacycles (Figure 1), which prompted us to explore the coordination chemistry of these three diamines with [(η^5^-C_5_Me_5_)RhCl]^+^, [(η^5^-C_5_Me_5_)IrCl]^+^ and [(η^6^-cymene)RuCl]^+^, in order to investigate what effect, if any, variations in the ring size of the azacycle had on potential stereoselective binding. Substitution-induced influences were of further interest, as discussed below.

There are a number of structural changes anticipated upon increasing the azacycle ring from five to six to seven; most notably, an increase in flexibility and access to additional conformers. With regard to the current complexes, unfavourable steric contacts between the (CH_2_)_n_ bridge in the azacycles and the “seat” of the piano-stool complexes were anticipated to promote stereoselective coordination, with the extent of such selectivity being dependent on the ring size and conformational preference of the diamine.

## 2. Results and Discussion

### 2.1. Synthesis and Characterisation of the Complexes

The synthesis of the [(η^5^-C_5_Me_5_)Rh(**L**)Cl]Cl, [(η^5^-C_5_Me_5_)Ir(**L**)Cl] Cl and [(η^6^-cymene)Ru(**L**)Cl] Cl complexes was very straightforward through the addition of the appropriate diamine (**L1**-**L3**) to solutions of the starting dimers in CH_2_Cl_2_ or MeOH. The complexes were isolated in essentially quantitative yield after the removal of all volatiles. For all the diamines in this study, there were two potential modes of attachment depending upon the relative orientation of the diamine with respect to the capping ligand (C_5_Me_5_ or cymene). The relative orientation dictates the stereochemistry at the newly developed sterogenic-at-metal centre, resulting in the formation of, potentially, two diastereomers. Inspection of the NMR spectra of [(η^5^-C_5_Me_5_)Rh(**L1**)Cl] Cl/PF_6_, **Rh-L1** showed an approximate 45:55 mixture of the two diastereomers, as evidenced by the well-separated, duplicate peaks in both the ^1^H and ^13^C NMR spectra (see ESI). Full assignment of each diastereomer was achieved by identifying through-space contacts between the cyclopentadienyl methyls and determinant hydrogens in the azacycle (see ESI). This lack of selectivity extended to both the [(η^5^-C_5_Me_5_)Ir(**L1**)Cl] Cl/PF_6_, **Ir-L1** (62:38 dr) and [(η^6^-cymene)Ru(**L1**)Cl] Cl/PF_6_, **Ru-L1** (60:40 dr) complexes, albeit with more pronounced major:minor isomer distributions.

**Figure 2:**
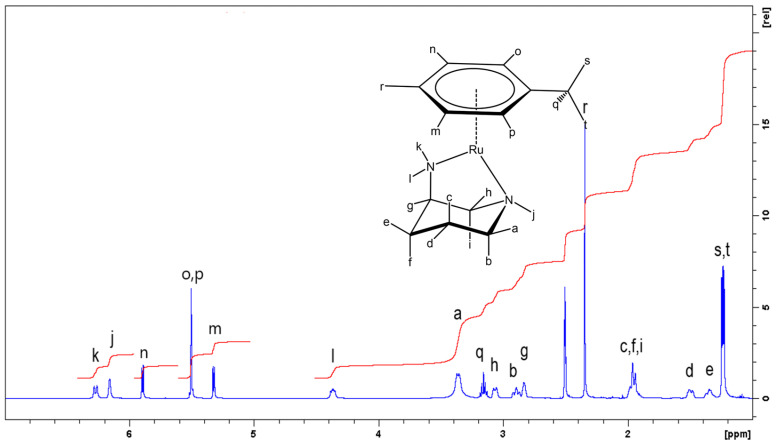
^1^H NMR spectrum of *S*-[(η^6^-cymene)Ru(**L2**)Cl] PF_6_ recorded at 500 MHz in D_6_-dmso.

It was not surprising that the smallest ligand **L1** showed little discrimination on coordination, but the poor ratios noted above were not encouraging for the subsequent study of the larger rings. However, when the same synthetic procedure was applied to the six-membered *R*-apip ligand (**L2**), only a single diastereomer was isolated in all cases, as confirmed upon inspection of the ^1^H and ^13^C NMR spectra of the complexes (see ESI and Figure 2). The stereoselectivity was highlighted in the fully-assigned ^1^H NMR spectrum of [(η^6^-cymene)Ru(**L2**)Cl] PF_6_ (Figure 2), where complete assignment was made possible by the acquisition of a series of 1D and 2D NMR experiments, and aided by the clear separation between peaks, as shown in the figure. All four aromatic hydrogens were inequivalent due to the stereochemically active metal centre, as were the two *iso*-propyl methyl groups. The mass spectrum showed the complex cation at 371.0824 amu. The presence of a single diastereomer was again evidenced in the ^13^C{^1^H} NMR spectrum, which contained a total of fifteen signals for the fifteen unique carbon environments.

**Figure 3:**
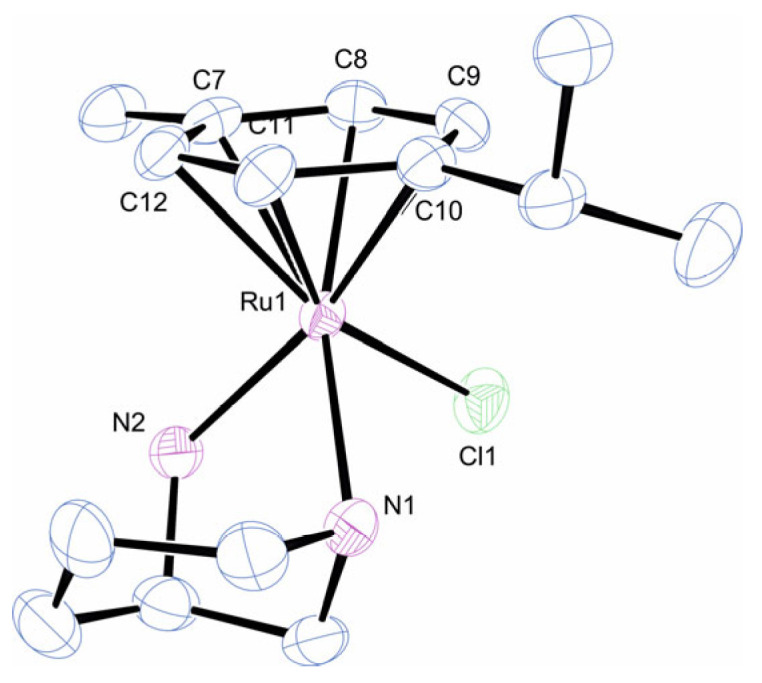
Molecular structure of *S*-[(η^6^-Cymene)Ru(**L2**)Cl]^+^. Anion and hydrogens omitted for clarity. Selected bond lengths (Å) and angles (°): Ru1-N1 2.173 (4); Ru1-N2 2.144 (4); Ru1-Cl1 2.3950 (13); Ru1-C7 2.186 (4); Ru1-C8 2.171 (5); Ru1-C9 2.175 (5); Ru1-C10 2.187 (4); Ru1-C11 2.167 (4); Ru1-C12 2.174 (4); N1-Ru1-N2 76.63 (15); N1-Ru1-Cl1 87.65 (12); N2-Ru1-Cl1 84.59 (12); N1-Ru1-centroid 133.94; N2-Ru1-centroid 132.94; and Cl1-Ru1-centroid 123.66.

To confirm the stereochemistry at the metal centre and to gain insight into the factors that contribute to the discrimination, single crystals of the ruthenium complex were grown and analysed by single-crystal X-ray diffraction (SCXRD). From the structure of the complex cation shown in Figure 3, it is evident that the *S* configuration was adopted at both the ruthenium centre and the secondary nitrogen. The stereochemistry of this nitrogen was fixed and determined solely by the chirality of the ring carbon, as bidentate coordination is only possible with the C(*R*)/N(*S*) combination. The azacycle adopted a chair conformation with the exo-NH_2_ group, projecting axially from the ring as is necessary for bidentate coordination, and the five-membered chelate had the δ conformation. The Ru-N bond lengths and N-Ru-N bond angles were very similar to those reported for the related complex with 2-(aminomethyl)piperidine, where values of 2.126(3), 2.153(3) Å and 79.47(11)⁰ were observed [24]. To emphasise the lack of structural data on metal complexes of **L2**, a recent search (26 June 2024) on the Cambridge Structural Database revealed only a single metal complex of the ligand; a copper(II) complex of the *S* enantiomer that shows similar geometric features in the diamine as seen here, except the chiral nitrogen is necessarily *R* in the reported complex. Inspection of the structure in Figure 3 does not reveal any obvious steric contacts that would explain the diastereoselectivity, with the trimethylene bridge of the azacycle directed towards the cymene ligand rather than away from it.

**Figure 4:**
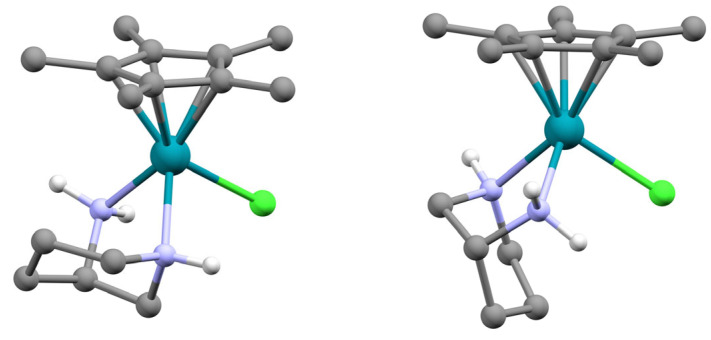
The two possible diastereomers of [(η^5^-C_5_Me_5_)Rh(**L2**)Cl]^+^. R_C_, S_N_, S_Rh_ (left image) and R_C_, S_N_, R_Rh_ (right).

Extension of the **L2** chemistry to form both [(η^5^-C_5_Me_5_)Rh(**L2**)Cl] PF_6_ and [(η^5^-C_5_Me_5_)Ir(**L2**)Cl] PF_6_ again led to the observation and isolation of a single diastereomer. This implies that both the η^5^-C_5_Me_5_ and η^6^-cymene units are sufficiently bulky to prevent the formation of the second possible diastereomer. The *S* configuration at the metal was maintained in all cases, as evidenced by selected contacts (or lack of) between certain CH_2_ hydrogens in the trimethylene bridge of the azacycle and the cymene or C_5_Me_5_ methyl groups in the 2D NOESY spectra; such contacts are not possible if the metals adopt the *R* configuration with **L2**. This is best illustrated by reference to the two possible diastereomers, R_C_, S_N_, S_Rh_ and R_C_, S_N_, R_Rh_ (Figure 4).

From the figure, it can be seen that in the assigned R_C_, S_N_, S_Rh_ diastereomer the through-space contacts to the C_5_Me_5_ methyls are with hydrogens in the trimethylene bridge as those at the two position of the heterocycle are pointed away. This is quite different in the R_C_, S_N_, R_Rh_ option, as these latter hydrogens are now contacting the C_5_Me_5_ methyls and the trimethylene bridge resides below the N_2_Cl plane. The only contacts seen in the NOESY spectra were between hydrogens at the four, five and six positions for the complexes of **L2** and the four, six and seven positions in the complexes of **L3**; no contacts with the C2 hydrogens were ever observed. Confirmation of this structure for [(η^5^-C_5_Me_5_)Rh(**L2**)Cl] PF_6_ was determined by SCXRD and is shown in the ESI.

**Figure 5:**
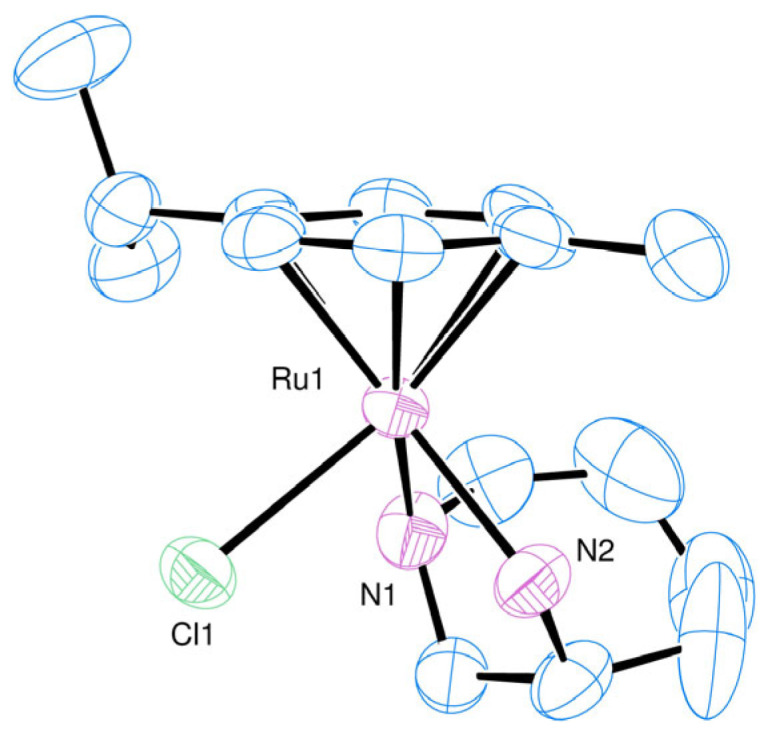
Molecular structure of *S*-[(η^6^-Cymene)Ru(**L3**)Cl]^+^. Anion and hydrogens omitted for clarity. Selected bond lengths (Å) and angles (°): Ru1-N1 2.159 (7); Ru1-N2 2.128 (6); Ru1-Cl1 2.4309 (18); N1-Ru1-N2 78.9 (3); N1-Ru1-Cl1 84.7 (2); and N2-Ru1-Cl1 83.92 (19).

As alluded above, the discrimination evident in the formation of the **L2** complexes was maintained for the **L3** complexes. There was no evidence of a second diastereomer in all cases. Analysis of the ^1^H NMR spectrum of [(η^6^-cymene)Ru(**L3**)Cl] PF_6_ showed the same inequivalency in the peaks of the cymene ligand as noted for **L2**. There was no spectral complexity associated with the presence of a second isomer in both the ^1^H and ^13^C NMR spectra. The same applied to the Rh and Ir complexes, as noted in the ESI. Examination of the solid-state structure of *S*-[(η^6^-Cymene)Ru(**L3**)Cl] PF_6_ (Figure 5) showed an analogous structure to that of the complex with **L2**, confirming the adoption of the *S* configuration at the ruthenium. The seven-membered ring adopted an asymmetric boat conformation, the five-membered chelate, the expected δ conformation with very similar metrics to those of the complex with **L2**. A search of the CSD (26 June 2024) database revealed less than ten structurally characterised complexes of **L3** with Cu (II), Pd (II) and Pt (II) as the only metal ions. It was therefore concluded that the 3-amino derivatives of the six- and seven-membered azacycles are capable of stereoselective coordination, whereas the five-membered is not.

### 2.2. Computational Studies of the Coordination Stereoselectivity

**Figure 6:**
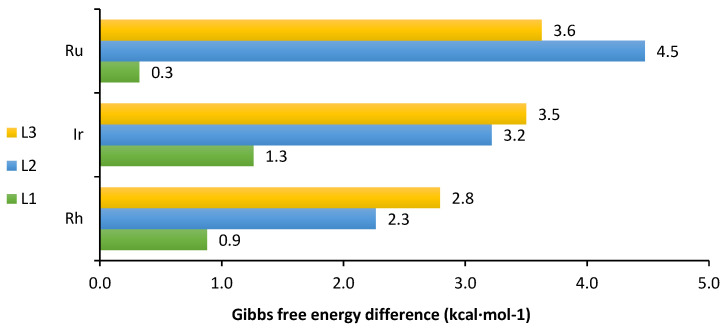
Relative Gibbs free energy preference for the metal centre configuration to be the same as the N1 atom; i.e., S_C_, R_N_, R_M_ for **L1** and R_C_, S_N_, S_M_ for **L2** and **L3**. Calculated in Orca 5.0.4 at the ωB97M-V/def2-QZVPPD/CPCM (DCM)//r^2^SCAN-3c/CPCM(DCM)/298.15 K [25].

Having established a ring size dependence on stereochemical control at the metal centre in these piano-stool complexes, it was prudent to provide some rationale as to the origin of the effect. The initial DFT calculations incorporated the following assumptions: the ligands will assume the most favourable ring configuration (envelope, chair and twist chair for L1, L2 and L3, respectively) and the primary amine groups were axially orientated as necessary for coordination. The results of the calculations (Figure 6) confirmed a preference for the geometries already established from the afore-mentioned spectroscopic and SCXRD studies. Although a thermodynamic preference was noted for all, the stability difference was significantly greater for the six and seven-membered azacycles than for the five-membered. The calculations showed that the energy difference between diastereomers increases as Rh < Ir < Ru for the six- and seven-membered azacycles, and Ru < Rh < Ir for the five-membered. Examining the distances between the carbon backbone of the azacycles and the corresponding polyhapto ligands did not give a clear indication for a preference of one configuration over another. However, we did observe that shorter C2-Cl distances seemed to be preferred for the complexes bearing the six- and seven-membered azacycles (see Supplementary Materials). This would likely be due to a favourable H-bond-type interaction between one of the protons at the C2 position and the chloride ligand. This is plausible considering the C2 carbon atom must inevitably bear a partial positive charge due to its proximity to the two nitrogens, which would mean that such H-bond-type interactions would, indeed, be feasible.

### 2.3. Asymmetric Transfer Hydrogenation Studies

Access to these stereogenic-at-metal complexes was encouraging, as we were interested in their application as catalysts for asymmetric transfer hydrogenation. Using 4-fluoroacetophenone as a substrate for convenient monitoring by ^19^F NMR spectroscopy and *S*-[(η^6^-Cymene)Ru(**L2**)Cl]Cl as a catalyst, a series of ATH reactions were performed. The reactions proved to be sluggish, requiring five days at 80 °C and five equivalents of potassium *tert*-butoxide for full conversion. In an effort to reduce the timeframe of the reductions, silver salts (AgBF_4_ or AgPF_6_) were employed as chloride abstraction agents, but this did not lead to enhanced conversion. Using the results from the ruthenium hydrogenations, a set of standard conditions was selected as follows: 1/5/100 catalyst/base/substrate ratio and a substrate concentration of 0.20 mol dm^−3^ in 5 cm^3^ of 2-propanol at reflux for approximately 3 days. The intermediate time of 3 days was selected (as opposed to the 5 days used initially) to allow for any differences in activity to manifest as differences in conversion. The results of these reactions are presented in Table 1 and indicate that all the complexes synthesised perform relatively well for the transfer hydrogenation of 4-fluoroacetophenone. Whilst accepting that the difference was marginal, the iridium complexes appeared to perform best, followed by rhodium and ruthenium. Chiral HPLC using a Chiralpak IF column with a silica-supported amylose stationary phase and hexane/2-propanol mixture as the mobile phase was employed to analyse the enantiomeric distribution in the products. Unfortunately, even though conversions were high, as shown in Table 1, all products were essentially racemic with enantiomeric excesses of <2%.

**Figure 7:**
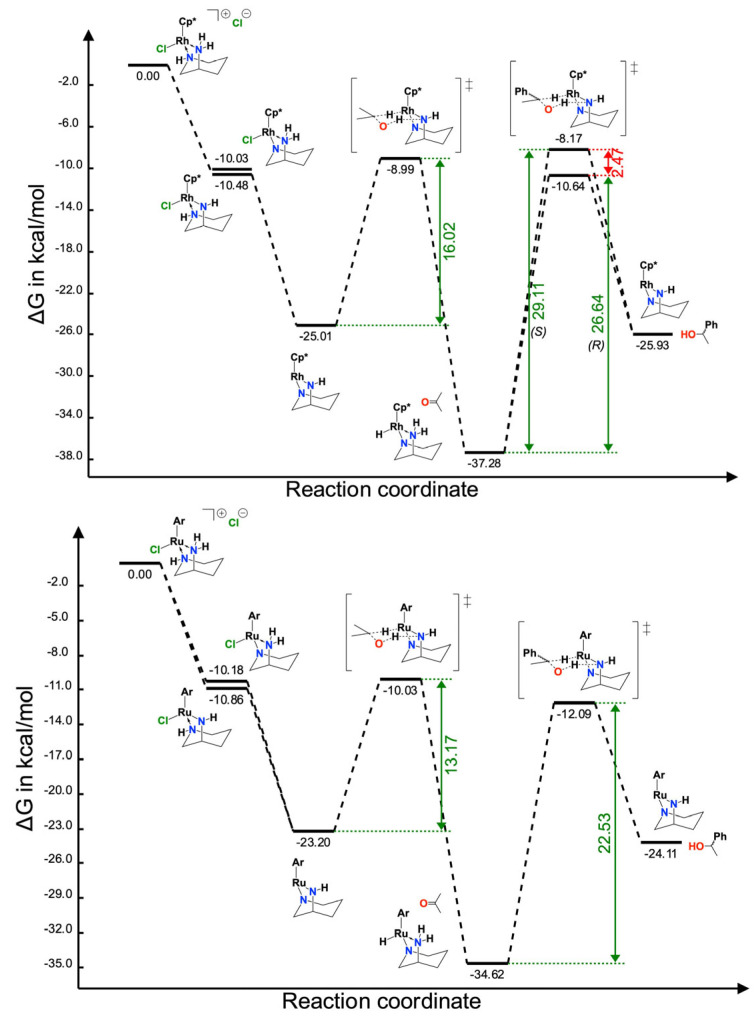
Energy profile for the ATH of acetophenone by *S*-[(η^5^-C_5_Me_5_)Rh(R-apip)Cl]^+^ (**top**) and *S*-[(η^6^-Cymene)Ru(R-apip)Cl]^+^ (**bottom**) where Cp* is η^5^-C_5_Me_5_. Calculated in Orca 5.0.4 at the ωB97M-V/def2-QZVPPD/CPCM(Ethanol)//r^2^SCAN-3c/CPCM(Ethanol)/353.15 K [25,26,28].

DFT calculations were performed using the **Ru-L2** and **Rh-L2** complexes and acetophenone as a template for trying to understand the lack of stereoselectivity in the ATH catalysis. The energy profiles and relevant species are shown in Figure 7. An initial two-step deprotonation is hereby proposed, whereby each nitrogen atom is deprotonated, which should be feasible, considering the basic conditions employed. These results indicate that there was little discrimination as to the first site of deprotonation, with only 0.68 (Ru) and 0.45 (Rh) kcal mol^−1^ preference for this to be the NH_2_ group. The second deprotonation yielded a neutral bis-amido species, which represents the active dehydrogenation species that generates a neutral hydride complex and acetone after abstraction of 2 × H from propan-2-ol. The loaded metal–hydride complex subsequently hydrogenated the acetophenone substrate to yield 1-phenylethanol, and concomitantly regenerated the initial active form of the catalyst. This is akin to the mechanism originally reported by Noyori [26]. In addition, our proposed bis-amido species is also similar to the one isolated by Noyori, with two RuN bonds of varying lengths [27]. From the calculations, the Ru-N2 bond is 1.926 Å and the RuN1 bond is 2.088 Å, whilst the Rh-N2 bond is 1.939 Å and the Ru-N1 bond is 2.090 Å (see ESI). This disparity mirrors that for Noyori’s system, which features Ru-N bonds of 2.065 Å and 1.897 Å, respectively. Noyori cited the shorter Ru-N function as the principal contributor to the observed distinct dehydrogenative activity for methanol, ethanol and 2-propanol. Our model also predicts the shorter M-N bond as the active protonation site, coupled with the fact that the N1 atom is geometrically constrained within the ring of the ligand. Overall, the calculated Gibbs free energies and barriers are reasonable for the reaction conditions with the resultant energy profile agreeing with the lack of observed enantioselectivity, with only a small difference (2.47 kcal mol^−1^) for Rh and no difference at all for Ru in the transition state energies leading to *R* or *S* products (Figure 7). For all transition states, we observed the resulting imaginary frequency vibration to be along the C-H-M coordinate, with no significant vibration along the O-H-N coordinate, where the proton was associated primarily with the nitrogen atom. This indicates that the proton transfer must occur along the path to the saddle point. This is not concerning and, indeed, one would expect a proton transfer to readily occur at the reaction conditions.

**Figure 8:**
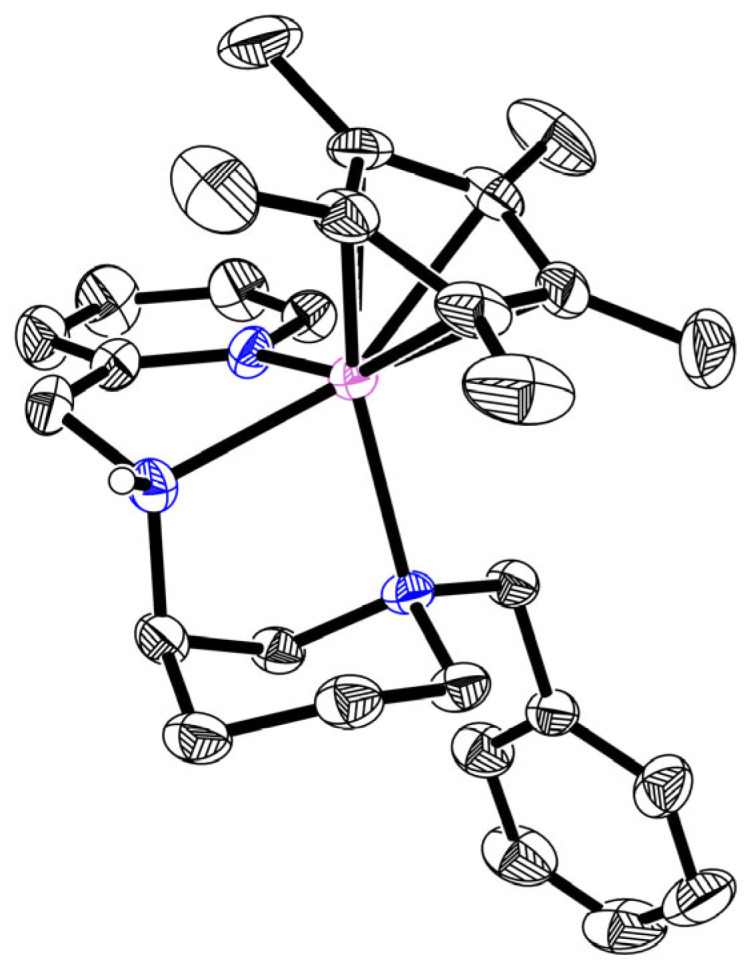
Molecular structure of [(η^5^-C_5_Me_5_) Rh (apippyr) Cl]^+^. Anion and hydrogens omitted for clarity. Pertinent metrics are given in the ESI.

A possible reason for the lack of stereo-control is the absence of projecting substituents at one or other of the nitrogen donors to provide steric impedance to the approaching acetophenone. For the bare diamines, there is insufficient spatial differentiation in the coordination sphere to encourage a selective approach of the ketone and promote stereoselectivity through enhancing the energy gap between the respective pro-*R* and pro-*S* transition states. If one of the amines in the azacycle were functionalised, then this may induce a degree of discrimination. To test the feasibility of this and the mechanism presented in Figure 7, we employed the *R*-1-benzyl-3-aminopiperidine ligand (Bzapip) and the subsequent complex [(η^5^-C_5_Me_5_)Rh(Bzapip)Cl]X (X = Cl or PF_6_^−^). The complex was made in the usual manner and the stereochemistry was confirmed as *S* at the metal by comparison of pertinent NMR spectra with those of the complex with the parent diamine. When the ATH was performed with this complex, either as the chloride or hexafluorophosphate salt, very poor conversion was observed compared to the [(η^5^-C_5_Me_5_)Rh(apip)Cl] X analogue under equivalent conditions. This observation supports the notion of the active, doubly deprotonated complex depicted in Figure 7. A final structural modification was then adopted, where a pyridyl group was introduced on the primary amine. This generated the coordinatively saturated [(η^5^-C_5_Me_5_)Rh(apippyr)Cl]X species, as shown in the molecular structure in Figure 8. The mechanism shown in Figure 7 demands the presence of an accessible coordination site for formation of M-H. Potential on/off coordination of the pyridine donor may release a binding site to allow for the formation of the necessary Rh-H function; however, this clearly was not the case as the complex proved to be a poor catalyst, highlighting the need for an available binding site.

## 3. Materials and Methods

All chemicals were purchased from commercial sources and used without further purification unless otherwise stated. All reactions and manipulations involving phosphines were performed under nitrogen using standard Schlenk techniques and previously dried, degassed solvents. NMR spectra were recorded on Bruker Fourier 300, DPX 400 and Avance 500 or 600 MHz NMR spectrometers (Billerica, MA, USA). ^1^H and ^13^C{^1^H} NMR chemical shifts were referenced relative to the residual solvent resonances in the deuterated solvent. Mass spectra (ESI) were recorded on a Waters LCT premier XE spectrometer (Temecula, CA, USA). UV/Vis spectra were obtained on a Cary 60 spectrophotometer and recorded over the range of 800 to 250 nm, with a 600 nm min^−1^ scan rate using a 1 cm path length quartz cuvette. Emission spectra were collected using a Cary Eclipse spectrophotometer from 700 to 450 nm, with an excitation wavelength of 410 nm and a 600 nm min^−1^ scan rate.

Single-crystal XRD data were collected using Mo-Kα radiation of 0.71073 Å on an Agilent SuperNova Dual Atlas (Santa Clara, CA, USA) diffractometer with a mirror monochromator. The sample temperature was maintained at 200 K using an Oxford Cryosystems cooling apparatus. The crystal structures were solved using SHELXT [29] and refined using SHELXL [30]. Non-hydrogen atoms were refined with anisotropic displacement parameters. Hydrogen atoms were inserted in idealised positions, and a riding model was used with their Uiso set at 1.2 or 1.5 times the value of Ueq for the atom to which they were bonded. CCDC 2380115–2380118 contain the supplementary crystallographic data for this paper. These data can be obtained free of charge via https://www.ccdc.cam.ac.uk/ (accessed on 1 June 2024) or from the CCDC, 12 Union Road, Cambridge CB2 1EZ, UK; fax: +44-1223-336033; e-mail: deposit@ccdc.cam.ac.uk. A table of pertinent details of the data collection and refinement is included in the ESI along with relevant spectroscopic and analytical data in Figures S1–S58.

DFT calculations were performed using the ORCA quantum chemistry software package (version 5.0.4) [25]. The relevant structures were subjected to geometry optimisation and confirmed as either stationary points (for ground states) or saddle points (for transition states) via vibrational frequency calculations. For all transition states, an accurate Hessian matrix was calculated for the initial input geometry and the transition states were confirmed as saddle points by the presence of a single imaginary (i.e., negative) vibrational frequency and by visualising the corresponding vibration using the “orca_pltvib” tool provided with the ORCA software. The conductor-like polarisable continuum model (CPCM) was used to model solvation [31]. A climbing image nudged elastic band plus zoomed climbing image nudged elastic band calculation (Zoom-NEB-CI) [32], along with the fast inertial relaxation engine (FIRE) optimisation method [33], as implemented in ORCA, were utilised to obtain an accurate transition state guess prior to eigenvector-following calculations. The geometry optimisation and vibrational frequency calculations were performed at the r2SCAN-3c level of theory (step 1), followed by singe-point energy calculations at the ωB97M-V/def2-QZVPPD level of theory, to obtain accurate electronic energies (step 2). To obtain the final Gibbs free energy, the following formula was used: G [1] − E[1] + E[2] where G is the Total Gibbs free energy and E is the electronic energy, with the relevant steps shown in the brackets. Convergence tolerances were set using the “verytightopt” and “verytightscf” keywords, along with setting “convcheckmode 0” in the “%scf” block, and “enforcestrictconvergence true” in the “%geom” block. The KDIIS algorithm was used for SCF convergence (ORCA switches this to a trust-region augmented Hessian (TRAH) algorithm [34,35] if convergence problems are detected). Other settings should be taken as defined by default in Orca 5.0.4. Examples of the input scripts used are available in the SI. The selection of the level of theory employed was guided by a publication on best practice DFT protocols by Grimme and co-workers [36]. Calculations were performed remotely using the supercomputing facilities at Cardiff University, operated by Advanced Research Computing at Cardiff (ARCCA) on behalf of the Cardiff Supercomputing Facility and the HPC Wales and Supercomputing Wales (SCW) projects.

### General Synthesis of the [(η^5^-C_5_Me_5_) M (L) Cl] X Complexes

To a stirred solution of the relevant [(η^5^-C_5_Me_5_)MCl_2_]_2_ complex (100 mg) in CH_2_Cl_2_ was added 2 mol equivalents of the appropriate diamine. After stirring for between 24 and 72 h, the solvents were removed in vacuo to give orange (Rh) to yellow (Ir) solids. The only exception was for Bzapip, which required a period of reflux in MeOH for complete reaction. The complexes were converted to the PF_6_^−^ salts by metathesis in water using NH_4_PF_6_.

*R,S-[(η^5^-C_5_Me_5_)Rh(S-amp)Cl]PF_6_*: yield = 77%. ^1^H (CDCl_3_, 400 MHz): δ_H_ (major isomer) 6.78 (d, J 10.0 Hz, 1H), 5.49 (s, 1H), 3.62 (s, 1H), 3.46 (m, 1H), 3.30 (t, J 15.1 Hz, 1H), 3.22 (m, 1H), 2.86 (m, 1H), 2.25 (m, 1H), 2.04 (d, J 14.0 Hz, 1H), 1.79 (s, 15H): δ_H_ (minor isomer) 6.82 (dd, *J*_HH_ *J*_HH_ 7.0, 3.2 Hz, 1H), 5.65 (s, 1H), 3.77 (s, 1H), 3.46 (m, 1H), 3.02 (d, J 14.5 Hz, 1H), 2.74 (m, 2H), 2.25 (m, 1H), 1.97 (m, 1H), 1.79 (s, 3H) ppm. ^13^C{^1^H} (CDCl_3_, 125 MHz): δ_C_ (major isomer) 95.2 (d, J 8.2 Hz, C), 56.6 (CH2), 52.4 (CH, CH2), 28.6 (CH2) 9.6 (CH3): δ_C_ (minor isomer) 95.1 (d, J 8.1 Hz, C), 62.2 (CH2), 50.6 (CH), 47.3 (CH2), 29.7 (CH2) 9.5 (CH3) ppm. HRMS (ES): *m*/*z* 371.1528 (calc. 371.1525) [L]^+^, 100%.

*R,S-[(η^5^-C_5_Me_5_)Ir(S-amp Cl]PF_6_*: yield = 89%. ^1^H (CDCl_3_, 400 MHz): δ_H_ (major isomer) 7.42 (d, J 10.3 Hz, 1H), 6.31 (s, 1H), 3.80-0.80 (m, 8H), 1.75 (s, 15H): δ_H_ (minor isomer) 7.67 (dd, *J*_HH_ *J*_HH_ 6.4, 3.3 Hz, 1H), 6.45 (s, 1H), 3.80-0.80 (m, 8H), 1.74 (s, 3H) ppm. ^13^C{^1^H} (CDCl_3_, 100 MHz): δ_C_ (major isomer) 86.6 (C), 57.2 (CH2), 53.4 (CH, CH2), 28.4 (CH2) 9.4 (CH3): δ_C_ (minor isomer) 86.5 (C), 64.2 (CH2), 53.4 (CH), 52.4 (CH2), 28.4 (CH2) 9.5 (CH3) ppm. HRMS (ES): *m*/*z* 449.1337 (calc. 449.1335) [M]^+^, 100%.

*R,S-[(η^6^-cymene)Ru(S-amp)Cl]PF_6_*: yield = 65%. ^1^H (CDCl_3_, 400 MHz): δ_H_ (major isomer) 8.01 (s br, 1H), 5.94 (m, 1H), 5.78–5.23 (m, 4H), 3.70–0.70 (m, 9H), 2.33 (s, 3H), 1.37–1.15 (m, 6H): δ_H_ (minor isomer) 8.21 (s br, 1H), 6.43 (s, 1H), 6.35 (s, 1H), 5.78–5.23 (m, 4H), 4.61 (s br, 1H), 3.70–0.70 (m, 7H), 2.41 (s, 3H), 1.37–1.15 (m, 6H) ppm. HRMS (ES): *m*/*z* 371.1528 (calc. 371.1525) [L]^+^, 100%.

*S-[(η^5^-C_5_Me_5_)Rh(R-apip)Cl]Cl*: yield = 99%. ^1^H (d_6_-DMSO, 300 MHz): δ_H_ 6.02 (s br, 1H), 5.08 (d, J 11.0 Hz, 1H), 4.54 (s br, 1H), 3.56–3.28 (m br, 3H), 3.10 (d, J 11.3 Hz, 1H), 2.99 (s, 1H), 2.73 (t, J 11.4 Hz, 1H), 2.18 (d, J 11.0 Hz, 1H), 1.88 (m, 2H), 1.65 (s, 15H) ppm. ^13^C{^1^H} (d_6_-DMSO, 100 MHz): δ_C_ 94.8 (d, J 8.1 Hz, C), 55.4 (CH2), 49.9 (CH2), 48.0 (CH), 27.8 (CH2) 19.3 (CH2), 9.5 (CH3) ppm. HRMS (ES): *m*/*z* 373.0918 (calc. 373.0918) [L]^+^, 20%.

*S-[(η^5^-C_5_Me_5_)Ir(R-apip)Cl]Cl*: yield = 99%. ^1^H (d_6_-DMSO, 300 MHz): δ_H_ 6.49 (s br, 1H), 5.64 (d, J 12.0 Hz, 1H), 5.19 (dd, J 11.5, 5.8 Hz, 1H), 3.71 (d, J 11.0 Hz, 1H), 3.00–2.85 (m br, 3H), 2.40 (d, J 11.4 Hz, 1H), 1.90–1.77 (m br, 2H), 1.62 (s, 15H), 1.49 (m, 2H) ppm. ^13^C{^1^H} (d_6_-DMSO, 100 MHz): δ_C_ 87.1 (C), 57.2 (CH2), 52.4 (CH2), 49.7 (CH), 26.7 (CH2) 19.6 (CH2), 9.8 (CH3) ppm. HRMS (ES): *m*/*z* 463.1479 (calc. 463.1492) [M]^+^, 70%.

*S-[(η^6^-cymene)Ru(R-apip)Cl]Cl*: yield = 99%. ^1^H (d_6_-DMSO, 500 MHz): δ_H_ 6.27 (d, J 11.0 Hz, 1H), 6.16 (s br, 1H), 5.90 (d, J 5.8 Hz, 1H), 5.50 (s, 2H), 5.33 (d, 5.6 Hz, 1H), 4.37 (dd, J 10.7, 6.2 Hz, 1H), 3.37 (m br, 1H), 3.16 (sep, J 6.8 Hz, 1H), 3.07 (d, J 10.7 Hz, 1H), 2.90 (m, 1H), 2.84 (m, 1H), 2.35 (s, 3H), 1.97 (m, 3H), 1.50 (m, 1H), 1.35 (m, 1H), 1.24 (d, J 6.8 Hz, 3H), 1.24 (d, J 6.8 Hz, 3H) ppm. ^13^C{^1^H} (d_6_-DMSO, 75 MHz): δ_C_ 103.2 (C), 98.5 (C), 84.1 (CH), 83.1 (CH), 82.9 (CH), 82.5 (CH), 54.9 (CH2), 53.7 (CH2), 48.1 (CH), 30.1 (CH), 27.4 (CH2), 24.2 (CH3), 21.5 (CH3), 19.7 (CH2), 18.2 (CH3) ppm. HRMS (ES): *m*/*z* 371.0824 (calc. 371.0828) [M]^+^, 100%.

*S-[(η^5^-C_5_Me_5_)Rh(R-ahaz)Cl]PF_6_*: yield = 87%. ^1^H (CDCl_3_, 500 MHz): δ_H_ 7.47 (d, J 10.6 Hz, 1H), 4.13 (s br 1H), 3.41 (m, 1H), 3.23 (m, 1H), 3.12 (m, 1H), 2.94 (t, J 11.2 Hz, 1H), 2.40 (d, J 13.4 Hz, 1H), 2.12 (m, 1H), 2.03–1.82 (m, 2H), 1.88 (m, 2H), 1.78 (s, 15H), 1.63 (m, 1H), 1.43 (m, 1H) ppm. ^13^C{^1^H} (CDCl_3_, 125 MHz): δ_C_ 95.9 (d, J 8.3 Hz, C), 54.7 (CH2), 53.5 (CH), 51.9 (CH2), 33.2 (CH2), 29.1 (CH2) 22.4 (CH2), 10.1 (CH3) ppm. HRMS (ES): *m*/*z* 387.1082 (calc. 387.1074) [M]^+^, 20%.

*S-[(η^5^-C_5_Me_5_)Ir(R-ahaz)Cl]PF_6_*: yield = 84%. ^1^H (CDCl_3_, 500 MHz): δ_H_ 7.74 (d, J 10.7 Hz, 1H), 4.61 (s br 1H), 3.70 (m br, 1H), 3.35 (m, 1H), 3.25 (m, 1H), 3.19 (m, 1H), 3.03 (m, 1H), 2.45 (d, J 12.8 Hz, 1H), 2.18 (m, 1H), 1.85 (m, 2H), 1.76 (s, 15H), 1.60-1.40 (m, 3H) ppm. ^13^C{^1^H} (CDCl_3_, 125 MHz): δ_C_ 87.3 (C), 56.1 (CH2), 54.6 (CH), 53.8 (CH2), 32.7 (CH2), 29.7 (CH2) 22.5 (CH2), 9.9 (CH3) ppm. HRMS (ES): *m*/*z* 477.1641 (calc. 477.1648) [M]^+^, 40%.

*S-[(η^6^-cymene)Ru(R-ahaz)Cl]PF_6_*: yield = 78%. ^1^H (CDCl_3_, 500 MHz): δ_H_ 8.17 (s br, 1H), 6.04 (d, J 5.8 Hz, 1H), 5.81 (d, J 5.8 Hz, 1H), 5.37 (s, 2H), 4.23 (s br, 1H), 3.47 (m br, 1H), 3.23 (m br, 1H), 3.09 (m, 3H), 2.86 (m, 1H), 2.50 (s, 3H), 2.23 (m, 1H), 2.15 (m, 1H), 1.98 (m, 1H), 1.86 (m, 2H), 1.78 (m, 1H), 1.40 (m, 1H), 1.25 (d, J 6.8 Hz, 3H), 1.23 (d, J 6.8 Hz, 3H) ppm. ^13^C{^1^H} (CDCl_3_, 125 MHz): δ_C_ 108.2 (C), 97.8 (C), 82.5 (CH), 82.2 (2 × CH), 80.2 (CH), 82.5 (CH), 55.8 (CH2), 54.2 (CH2), 53.3 (CH), 32.2 (CH2), 30.8 (CH), 29.0 (CH2), 23.5 (CH3), 22.9 (CH2), 21.4 (CH3), 19.5 (CH3) ppm. HRMS (ES): *m*/*z* 385.0990 (calc. 385.0985) [M]^+^, 100%.

*S-[(η^5^-C_5_Me_5_)Rh(R-Bzapip)Cl]Cl*: yield = 91%. ^1^H (d_6_-DMSO, 300 MHz): δ_H_ 7.52–7.32 (m, 5H), 5.22 (d, J 11.4 Hz, 1H), 4.79 (d, J 13.1 Hz, 1H), 4.68 (s br, 1H), 4.20 (d, J 13.1 Hz, 1H), 3.60–3.23 (m br, 4H), 3.18 (s br, 1H), 2.86–2.35 (m, 2H), 2.02 (m, 1H), 1.74 (s, 15H), 1.20 (m, 1H) ppm. ^13^C{^1^H} (d_6_-DMSO, 75 MHz): δ_C_ 132.4 (CH), 132.1 (CH), 129.2 (C), 129.0 (CH), 128.7 (CH), 128.6 (CH), 95.5 (d, J 8.2 Hz, C), 65.1 (CH2), 59.5 (CH2), 55.4 (CH2), 49.2 (CH), 26.6 (CH2) 20.3 (CH2), 9.5 (CH3) ppm. HRMS (ES): *m*/*z* 463.1386 (calc. 463.1387) [M]^+^, 10%.

*R-Bzapippyr.* A solution of Bzapip (0.5 g, 2.63 mmol) and 2-pyridinecarboxaldehyde (0.28 g, 2.63 mmol) in EtOH (40 ml) was heated at reflux for 1 h. After cooling, the volatiles were removed in vacuo and the residue redissolved in EtOH (40 ml). To the stirred solution was added solid NaBH_4_ (0.2 g, excess), portionwise, over 1 h, and the mixture was stirred at RT overnight. On return, the solution was acidified with conc. HCl (0.5 ml) and the volume reduced in vacuo to ~5 ml. Water (30 ml) was added, along with solid NaOH, until the solution was strongly basic and the aqueous mixture was subsequently extracted with CH_2_Cl_2_ (2 × 50 ml). The isolated organic phase was dried over MgSO_4_, filtered, and the volatiles removed to give a viscous, light-yellow oil. Yield = 0.69 g (88%). ^1^H (CDCl_3_, 400 MHz): δ_H_ 8.46 (d, J 4.9 Hz, 1H), 7.54 (td, J 7.6, 1.7 Hz, 1H), 7.25–7.13 (m, 6H), 7.06 (dd, J 7.5, 6.9 Hz, 1H), 3.85 (d, J 14.1 Hz, 1H), 3.81 (d, J 14.1 Hz, 1H), 3.44 (s, 2H), 2.81 (d, J 10.1 Hz, 1H), 2.69 (sep, J 4.1 Hz, 1H), 2.57 (m, 1H), 2.01 (t, J 10.0 Hz, 1H), 1.92 (t, J 8.5 Hz, 1H), 1.81 (m, 1H), 1.63 (m, 1H), 1.49 (m, 1H), 1.18 (m, 1H) ppm. ^13^C{^1^H} (d_6_-acetone, 75 MHz): δ_C_ 159.9 (C), 149.2 (CH), 138.3 (C), 136.4 (CH), 129.2 (CH), 128.2 (CH), 127.0 (CH), 122.3 (CH), 121.9 (CH), 63.3 (CH2), 59.4 (CH), 54.0 (CH2), 53.8 (CH2), 52.4 (CH2), 31.0 (CH2) 23.7 (CH2) ppm. HRMS (ES): *m*/*z* 664.1761 (calc. 664.1763) [M]^+^, 100%.

*S-[(η^5^-C_5_Me_5_)Rh(R-Bzapippyr)Cl]PF_6_*: yield = 67%. ^1^H (d_6_-acetone, 300 MHz): δ_H_ 9.14 (d, J 5.5 Hz, 1H), 8.21 (td, J 7.8, 1.4 Hz, 1H), 7.84 (m, 2H), 7.30–7.09 (m, 5H), 6.46 (d, J 4.6 Hz, 1H), 4.90 (dd, J 17.4, 5.7 Hz, 1H), 4.64 (d, J 17.5 Hz, 1H), 4.56 (d, J 13.7 Hz, 1H), 4.25 (d, J 13.3 Hz, 1H), 3.45 (d, J 12.3 Hz, 1H), 3.34 (s br, 1H), 2.59 (m, 1H), 2.14 (m, 1H), 1.75 (s, 15H), 1.38 (m, 1H) ppm. ^13^C{^1^H} (d_6_-acetone, 75 MHz): δ_C_ 161.9 (C), 151.8 (CH), 141.4 (CH), 132.0 (CH), 130.1 (C), 129.2 (CH), 128.4 (CH), 127.9 (CH), 124.6 (CH), 98.8 (d, J 7.9 Hz, C), 65.9 (CH2), 62.6 (CH), 62.3 (CH2), 59.5 (CH2), 55.5 (CH2), 26.8 (CH2) 20.4 (CH2), 8.4 (CH3) ppm. HRMS (ES): *m*/*z* 282.1971 (calc. 282.1970) [L + H]^+^, 100%.

## 4. Conclusions

The selective coordination of asymmetric 3-aminoazacycles to [(η^5^-C_5_Me_5_)RhCl]^+^, [(η^5^-C_5_Me_5_)IrCl]^+^ and [(η^6^-cymene)RuCl]^+^ cores is controlled by the azacyclic ring size. The smallest five-membered azacycle ligand shows no selectivity, whereas the larger six- and seven-membered derivatives are essentially stereospecific. The compounds are active for transfer hydrogenation, but rates are sluggish and the resulting alcohols are formed with very low enantioselectivity. DFT analysis has revealed very little asymmetric discrimination in key reaction steps to explain the lack of stereoselectivity in the transfer hydrogenation of prochiral ketones.

## Figures and Tables

**Table 1:** Results for the transfer hydrogenation of 4-fluoroacetophenone in propan-2-ol *^a^*.

Complex	Conversion (%) *^b^*	e.e. (%) *^c^*
Rh-L1	98	1.8
Rh-L2	89	0.5
Rh-L3	95	1.6
Ir-L1	98	0.4
Ir-L2	97	0.2
Ir-L3	97	0.3
Ru-L1	90	0.6
Ru-L2	90	1.8
Ru-L3	94	0.3

*^a^* T = 82 ⁰; KO*^t^*Bu equivalents = 5; substrate equivalents = 100; substrate concentration = 0.2 mol dm^−3^; total volume (including substrate) = 5.1 cm^−3^; time = 68 h. Reactions performed in microwave vials in the dark, each data set is an average of two independent catalytic runs. *^b^* determined by ^19^F NMR spectroscopy. *^c^* determined by chiral HPLC: Chiralpak IF column, 99:1 Hexane: 2-propanol, 2.0 mL/min, 211 nm detection, sample filtered through a 0.2 μm PTFE filter, followed by a plug of alumina prior to analysis.

## Data Availability

Data are contained within the article and Supplementary Materials.

## Supplementary Materials

The following supporting information can be downloaded at: https://www.mdpi.com/article/10.3390/molecules29194659/s1: Figures S1–S58: NMR and mass spectra of the compounds. Crystal data and molecular structures and Orca input commands.
