# Supplementary material for: Ringing the Changes: Effects of Heterocyclic Ring Size on Stereoselectivity in [(η5-C5Me5)RhCl], [(η5-C5Me5)IrCl] and [Ru(η6-cymene)Cl] Complexes of Chiral 3-Amino-1-Azacycles
Source: Molecules. 2024 Sep 30;29(19):4659. doi: 10.3390/molecules29194659 (PMC11478173; doi:10.3390/molecules29194659)
Supplement: Supplementary file 1 [file molecules-29-04659-s001.zip › molecules-3209672-supplementary.pdf]

Supplementary materials

Ringing the Changes: Effects of Heterocyclic Ring Size on Stereoselectivity in [(η^5-C_5Me_5)RhCl], [(η^5-C_5Me_5)IrCl] and [Ru(η^6-cymene)Cl] Complexes of Chiral 3-Amino-1-Azacycles

Vladimir Y. Vladimirov, Matheo Charrier-Chardin, Benson M. Kariuki, Benjamin D. Ward and Paul D. Newman *

School of Chemistry, Cardiff University, Cardiff CF10 3AT, UK; vladimirovvy@cardiff.ac.uk (V.Y.V.); matheo.charrierchardin@gmail.com (M.C.-C.); kariukib@cardiff.ac.uk (B.M.K.); wardbd@cardiff.ac.uk (B.D.W.)
* Correspondence: newmanp1@cardiff.ac.uk; Tel.: +44-(0)2920-870464

Contents

Figure S1 ^{1}H NMR spectra of *R,S*-[(η^5-C_5Me_5)Rh(*S*-amp)Cl]PF_6 recorded at 500 MHz in $CDCl_3$. S5

Figure S2 ^{13}C{^{1}H} NMR spectrum of *R,S*-[(η^5-C_5Me_5)Rh(*S*-amp)Cl]PF_6 recorded at 125 MHz in $CDCl_3$. S5

Figure S3 COSY NMR spectrum of *R,S*-[(η^5-C_5Me_5)Rh(*S*-amp)Cl]PF_6 recorded at 125 MHz in $CDCl_3$. S6

Figure S4 NOESY NMR spectrum of *R,S*-[(η^5-C_5Me_5)Rh(*S*-amp)Cl]PF_6 recorded at 125 MHz in $CDCl_3$. S6

Figure S5 HSQC NMR spectrum of *R,S*-[(η^5-C_5Me_5)Rh(*S*-amp)Cl]PF_6 recorded at 125 MHz in $CDCl_3$. S7

Figure S6 HRMS (ES^+) spectrum of *R,S*-[(η^5-C_5Me_5)Rh(*S*-amp)Cl]PF_6. S8

Figure S7 ^{1}H NMR spectra of *R,S*-[(η^5-C_5Me_5)Ir(*S*-amp)Cl]PF_6 recorded at 500 MHz in $CDCl_3$. S8

Figure S8 ^{13}C{^{1}H} NMR spectrum of *R,S*-[(η^5-C_5Me_5)Ir(*S*-amp)Cl]PF_6 recorded at 125 MHz in $CDCl_3$. S9

Figure S9 HRMS of *R,S*-[(η^5-C_5Me_5)Ir(*S*-amp)Cl]PF_6 . S10

Figure S10 ^{1}H NMR spectrum of *R,S*-[(η^6-cym)Ru(*S*-amp)Cl]PF_6 recorded at 500 MHz in $CDCl_3$. S10

Figure S11 ^{13}C{^{1}H} NMR spectrum of *R,S*-[(η^6-cym)Ru(*S*-amp)Cl]PF_6 recorded at 125 MHz in $CDCl_3$. S10

Figure S12 HRMS of *R,S*-[(η^6-cym)Ru(*S*-amp)Cl]PF_6. S11

Figure S13 ^{1}H NMR spectra of [(η^5-C_5Me_5)Rh(*R*-apip)Cl]Cl recorded at 300 MHz in D_6-dmso. S12

Figure S14 Labelled ^{1}H NMR spectrum of [(η^5-C_5Me_5)Rh(*R*-apip)Cl]Cl recorded at 300 MHz in D_6-dmso. S13

Figure S15 ^{13}C{^{1}H} NMR spectrum of [(η^5-C_5Me_5)Rh(*R*-apip)Cl]Cl recorded at 100 MHz in D_6-dmso. S14

Figure S16 ^{1}H-^{1}H COSY NMR spectrum of [(η^5-C_5Me_5)Rh(*R*-apip)Cl]Cl recorded at 100 MHz in D_6-dmso. S14

Figure S17 ^{1}H-^{1}H NOESY NMR spectrum of [(η^5-C_5Me_5)Rh(*R*-apip)Cl]Cl recorded at 100 MHz in D_6-dmso. S14

Figure S18 ^{13}C-^{1}H HSQC NMR spectrum of [(η^5-C_5Me_5)Rh(*R*-apip)Cl]Cl recorded at 100 MHz in D_6-dmso. S15

Figure S19 HRMS (ES$^+$) spectrum of [(η^5-C_5Me_5)Rh(*R*-apip)Cl]Cl. S16

Figure S20 ^{1}H NMR spectra of [(η^5-C_5Me_5)Ir(*R*-apip)Cl]Cl recorded at 300 MHz in D_6-dmso. S16

Figure S21 ^{13}C{^{1}H} NMR spectrum of [(η^5-C_5Me_5)Ir(*R*-apip)Cl]Cl recorded at 100 MHz in D_6-dmso. S17

Figure S22 HRMS (ES$^+$) spectrum of [(η^5-C_5Me_5)Ir(*R*-apip)Cl]Cl. S18

Figure S23 ^{1}H NMR spectrum of *R,S*-[(η^6-cym)Ru(*S*-apip)Cl]PF_6 recorded at 500 MHz in d_6-DMSO. S18

Figure S24 ^{13}C{^{1}H} NMR spectrum of *R,S*-[(η^6-cym)Ru(*S*-apip)Cl]PF_6 recorded at 500 MHz in d_6-DMSO. S18

Figure S25 HSQC NMR spectrum of *R,S*-[(η^6-cym)Ru(*S*-apip)Cl]PF_6 recorded at 500 MHz in d_6-DMSO. S19

Figure S26 ^{1}H-^{1}H COSY NMR spectrum of *R,S*-[(η^6-cym)Ru(*S*-apip)Cl]PF_6 at 500 MHz in d_6-DMSO. S19

Figure S27 ^{1}H-^{1}H NOESY NMR spectrum of *R,S*-[(η^6-cym)Ru(*S*-apip)Cl]PF_6 at 500 MHz in d_6-DMSO. S20

Figure S28 HRMS of *R,S*-[(η^6-cym)Ru(*S*-apip)Cl]PF_6 recorded at 500 MHz in d_6-DMSO. S21

Figure S29 ^{1}H NMR spectrum of [(η^5-C_5Me_5)Rh(*R*-ahaz)Cl]PF_6 recorded at 500 MHz in $CDCl_3$. S21

Figure S30 ^{13}C{^{1}H} NMR spectrum of [(η^5-C_5Me_5)Rh(*R*-ahaz)Cl]PF_6 recorded at 500 MHz in $CDCl_3$. S22

Figure S31 HRMS of [(η^5-C_5Me_5)Rh(*R*-ahaz)Cl]PF_6. S23

Figure S32 ^{1}H NMR spectra of [(η^5-C_5Me_5)Ir(*R*-ahaz)Cl]Cl recorded at 300 MHz in D_6-dmso. S24

Figure S33 ^{13}C{^{1}H} NMR spectrum of [(η^5-C_5Me_5)Ir(*R*-ahaz)Cl]Cl recorded at 300 MHz in D_6-dmso. S25

Figure S34 ^{1}H-^{1}H COSY NMR spectrum of [(η^5-C_5Me_5)Ir(*R*-ahaz)Cl]Cl recorded at 300 MHz in D_6-dmso. S25

Figure S35 ^{1}H-^{1}H NOESY NMR spectrum of [(η^5-C_5Me_5)Ir(*R*-ahaz)Cl]Cl recorded at 300 MHz in D_6-dmso. S26

Figure S36 ^{13}C-^{1}H HSQC NMR spectrum of [(η^5-C_5Me_5)Ir(*R*-ahaz)Cl]Cl recorded at 300 MHz in D_6-dmso. S26

Figure S37 HRMS of [(η^5-C_5Me_5)Ir(*R*-ahaz)Cl]Cl. S27

Figure S38 ^{1}H NMR spectrum of [(η^6-cym)Ru(*R*-ahaz)Cl]Cl recorded at 300 MHz in D_6-dmso. S27

Figure S39 ^{13}C{^{1}H} NMR spectrum of [(η^6-cym)Ru(*R*-ahaz)Cl]Cl recorded at 300 MHz in D_6-dmso. S28

Figure S40 ^{1}H-^{1}H COSY NMR spectrum of [(η^6-cym)Ru(*R*-ahaz)Cl]Cl recorded at 300 MHz in D_6-dmso. S28

Figure S41 ^{1}H-^{1}H NOESY NMR spectrum of [(η^6-cym)Ru(*R*-ahaz)Cl]Cl recorded at 300 MHz in D_6-dmso. S29

Figure S42 ^{13}C-^{1}H HSQC NMR spectrum of [(η^6-cym)Ru(*R*-ahaz)Cl]Cl recorded at 300 MHz in D_6-dmso. S29

Figure S43 HRMS of [(η^6-cym)Ru(*R*-ahaz)Cl]Cl. S30

Figure S44 ^{1}H NMR spectra of [(η^5-C_5Me_5)Rh(*R*-Bzapip)Cl]Cl recorded at 300 MHz in D_6-dmso. S31

Figure S45 $^{13}C\{^1H\}$ NMR spectra of [(η^5-C_5Me_5)Rh(*R*-Bzapip)Cl]Cl recorded at 300 MHz in D_6-dmso. S32

Figure S46 ^1H-^1H COSY NMR spectra of [(η^5-C_5Me_5)Rh(*R*-Bzapip)Cl]Cl recorded at 300 MHz in D_6-dmso. S32

Figure S47 ^1H-^1H NOESY NMR spectra of [(η^5-C_5Me_5)Rh(*R*-Bzapip)Cl]Cl recorded at 300 MHz in D_6-dmso. S33

Figure S48 ^{13}C-^1H HSQC NMR spectra of [(η^5-C_5Me_5)Rh(*R*-Bzapip)Cl]Cl recorded at 300 MHz in D_6-dmso. S33

Figure S49 HRMS of [(η^5-C_5Me_5)Rh(*R*-Bzapip)Cl]Cl. S34

Figure S50 ^1H NMR spectrum of *R*-Bzapippyr recorded at 400 MHz in $CDCl_3$. S35

Figure S51 $^{13}C\{^1H\}$ NMR spectrum of *R*-Bzapippyr recorded at 400 MHz in $CDCl_3$. S35

Figure S52 HRMS of *R*-Bzapippyr. S36

Figure S53 ^1H NMR spectra of [(η^5-C_5Me_5)Rh(*R*-Bzapippyr)Cl]PF_6 recorded at 300 MHz in D_6-acetone. S37

Figure S54 $^{13}C\{^1H\}$ NMR spectra of [(η^5-C_5Me_5)Rh(*R*-Bzapippyr)Cl]PF_6 at 300 MHz in D_6-acetone. S39

Figure S55 ^1H-^1H COSY NMR spectra of [(η^5-C_5Me_5)Rh(*R*-Bzapippyr)Cl]PF_6 recorded at 300 MHz in D_6-acetone. S39

Figure S56 ^1H-^1H NOESY NMR spectra of [(η^5-C_5Me_5)Rh(*R*-Bzapippyr)Cl]PF_6 recorded at 300 MHz in D_6-acetone. S39

Figure S57 ^{13}C-^1H HSQC NMR spectra of [(η^5-C_5Me_5)Rh(*R*-Bzapippyr)Cl]PF_6 recorded at 300 MHz in D_6-acetone. S40

Figure S58 HRMS of [(η^5-C_5Me_5)Rh(*R*-Bzapippyr)Cl]PF_6. S41

Crystallographic data S42

Orca input commands S45

NMR and mass spectra of *R,S*-[(η^5-C_5Me_5)Rh(*S*-amp)Cl]PF_6.

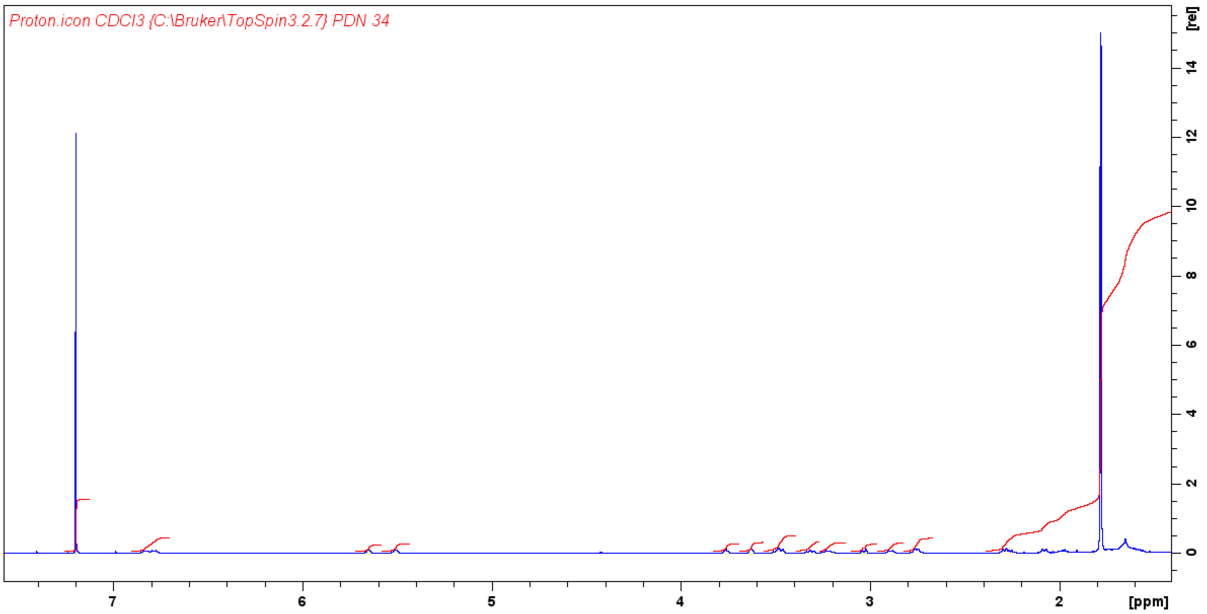

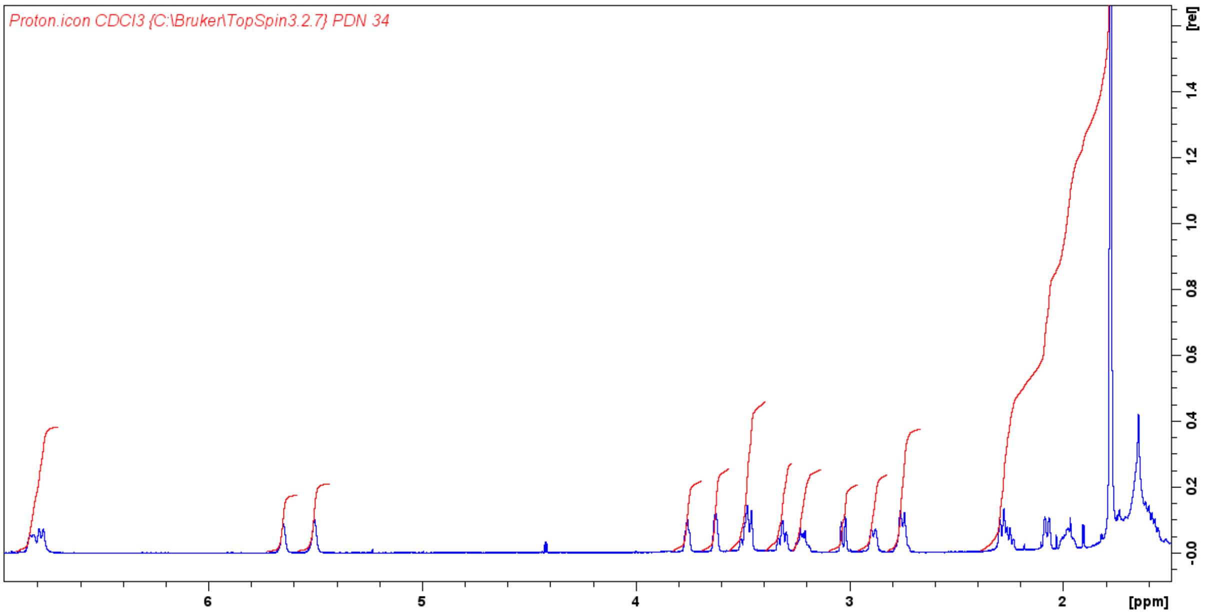

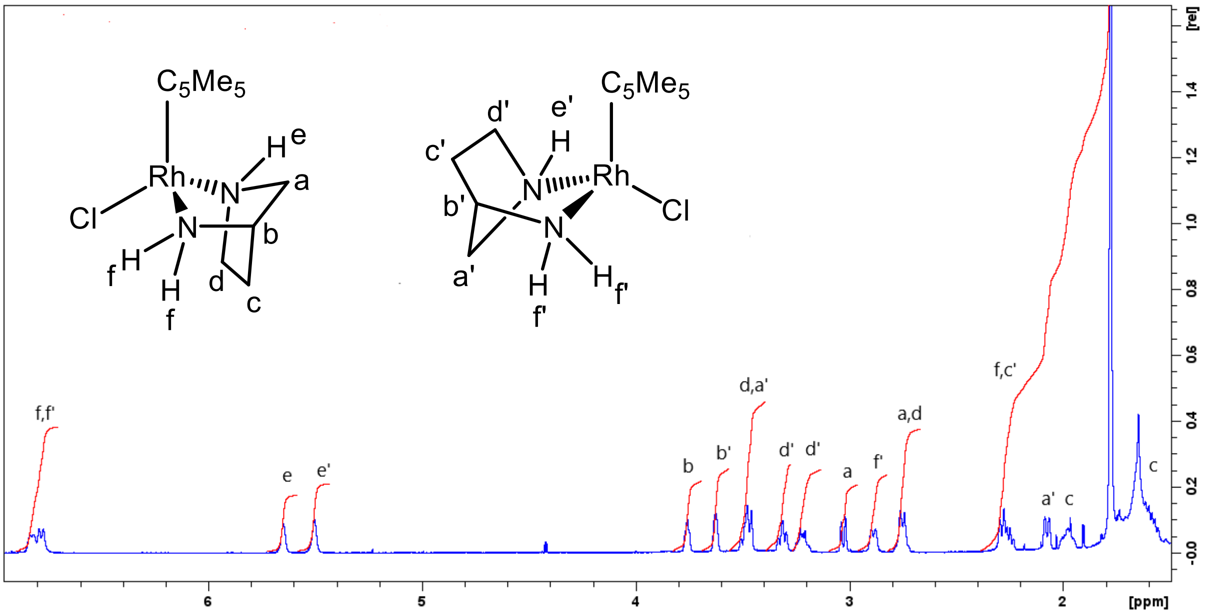

Figure S1 ^{1}H NMR spectra of *R,S*-[(η^5-C_5Me_5)Rh(*S*-amp)Cl]PF_6 recorded at 500 MHz in $CDCl_3$.

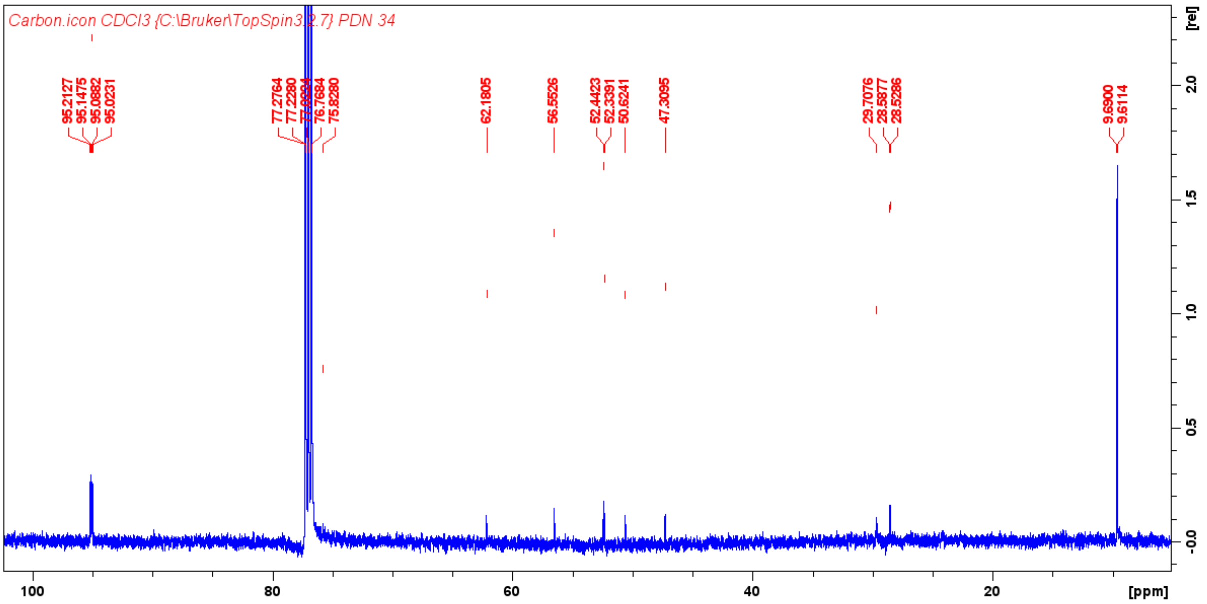

Figure S2 ^{13}C{^{1}H} NMR spectrum of *R,S*-[(η^5-C_5Me_5)Rh(*S*-amp)Cl]PF_6 recorded at 125 MHz in $CDCl_3$.

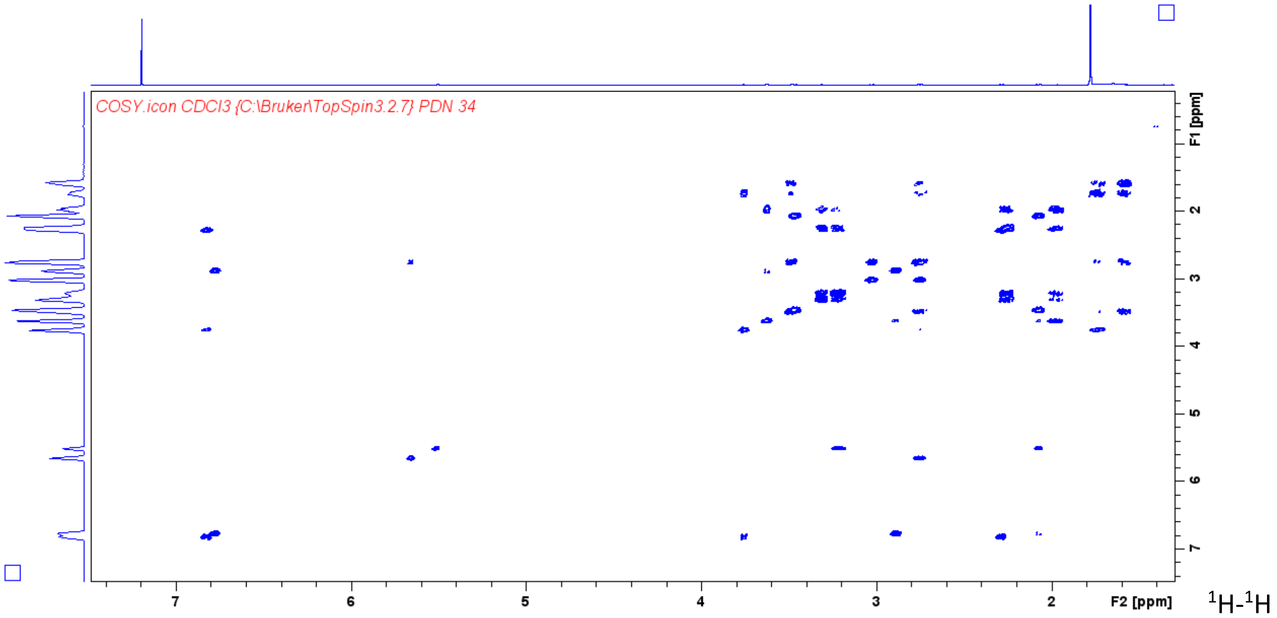

Figure S3 COSY NMR spectrum of *R,S*-[(η^5-C_5Me_5)Rh(*S*-amp)Cl]PF_6 recorded at 125 MHz in $CDCl_3$.

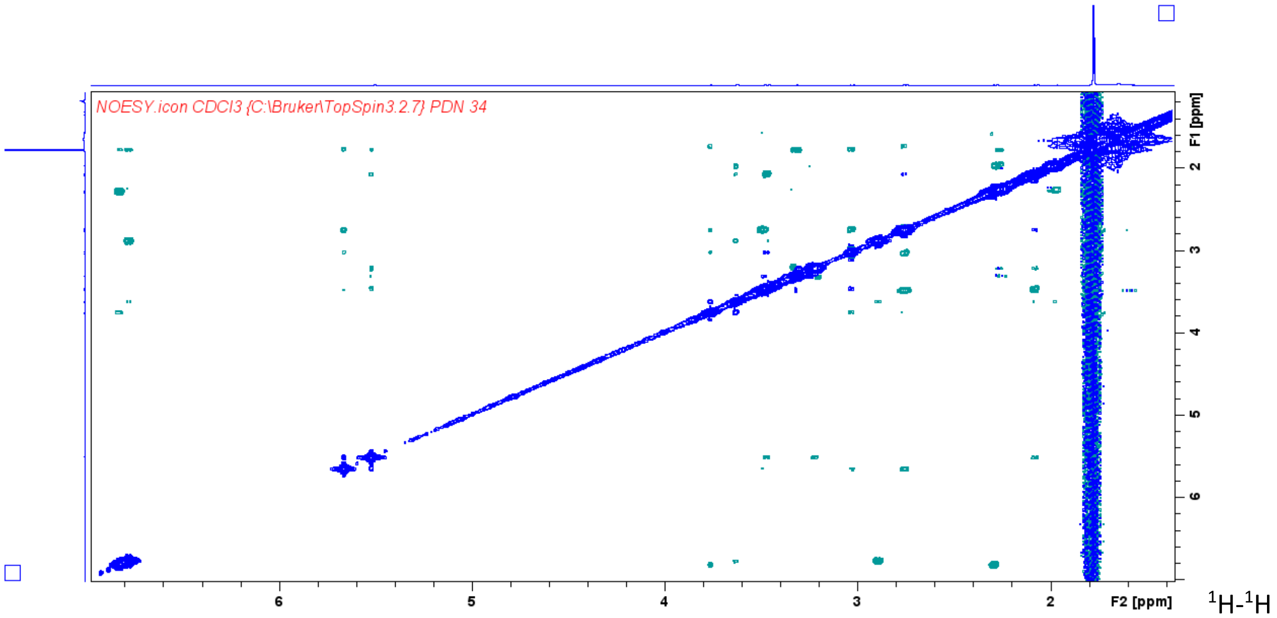

Figure S4 NOESY NMR spectrum of *R,S*-[(η^5-C_5Me_5)Rh(*S*-amp)Cl]PF_6 recorded at 125 MHz in $CDCl_3$.

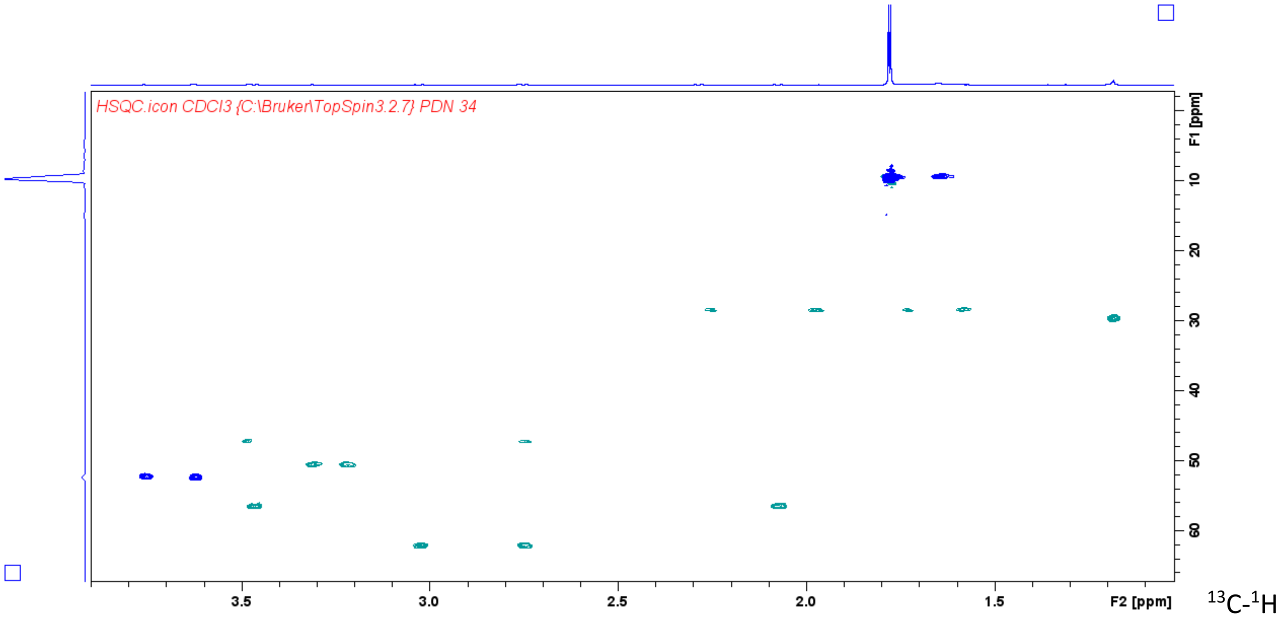

Figure S5 HSQC NMR spectrum of *R,S*-[(η[5]-C_5Me_5)Rh(*S*-amp)Cl]PF_6 recorded at 125 MHz in $CDCl_3$.

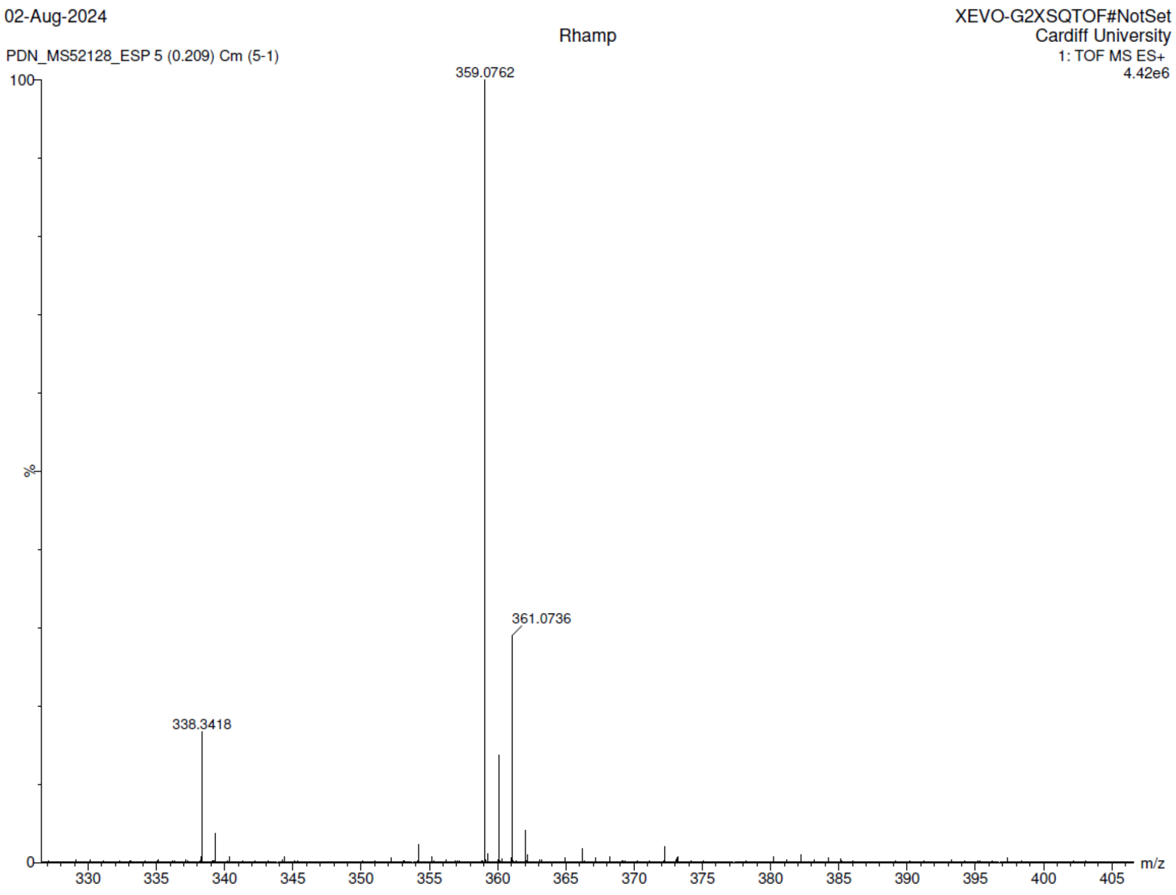

Minimum:				-1.5				
Maximum:		5.0	10.0	50.0				
Mass	Calc. Mass	mDa	PPM	DBE	i-FIT	Norm	Conf(%)	Formula
359.0762	359.0761	0.1	0.3	3.0	1577.0	n/a	n/a	C14 H25 N2 Cl Rh

Figure S6 HRMS (ES^+) spectrum of *R,S*-[(η^5-C_5Me_5)Rh(*S*-amp)Cl]PF_6.

NMR and mass spectra of *R,S*-[(η^5-C_5Me_5)Ir(*S*-amp)Cl]PF_6.

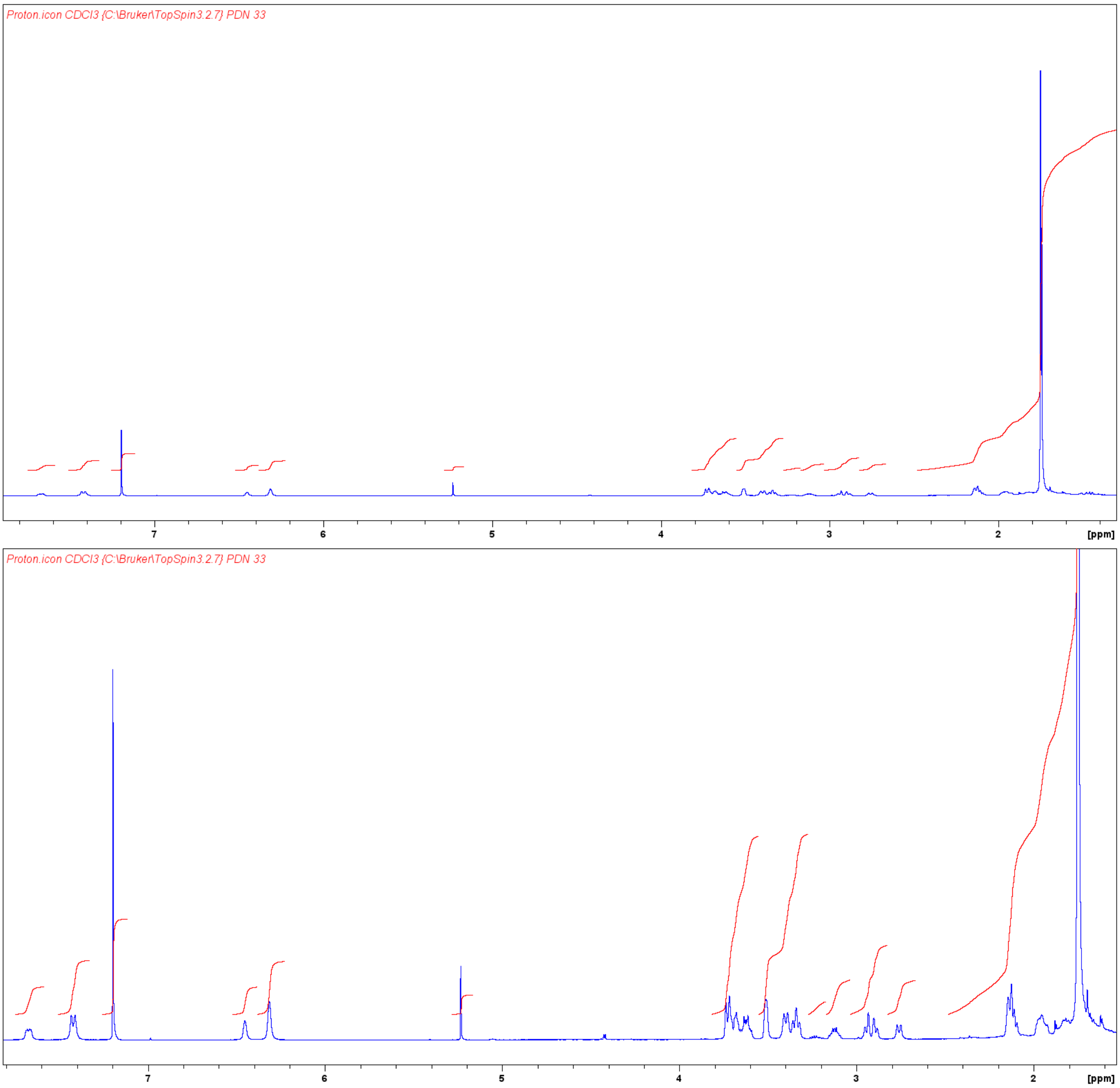

Figure S7 ^1H NMR spectra of *R,S*-[(η^5-C_5Me_5)Ir(*S*-amp)Cl]PF_6 recorded at 500 MHz in $CDCl_3$.

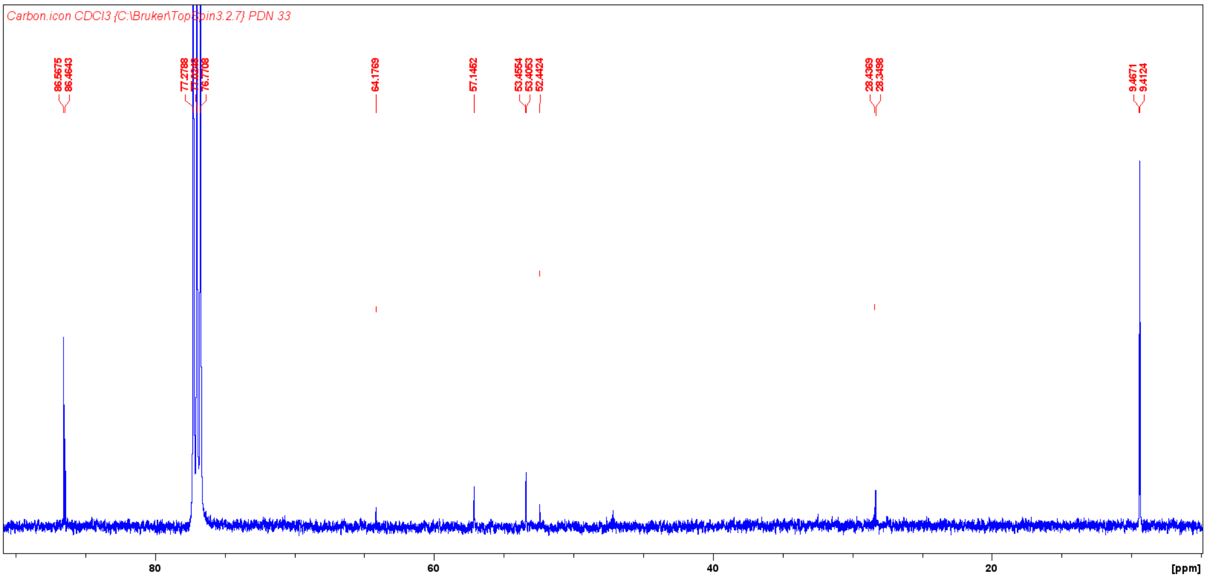

Figure S8 $^{13}C\{^{1}H\}$ NMR spectrum of *R,S*-[(η^5-C_5Me_5)Ir(*S*-amp)Cl]PF_6 recorded at 125 MHz in $CDCl_3$.

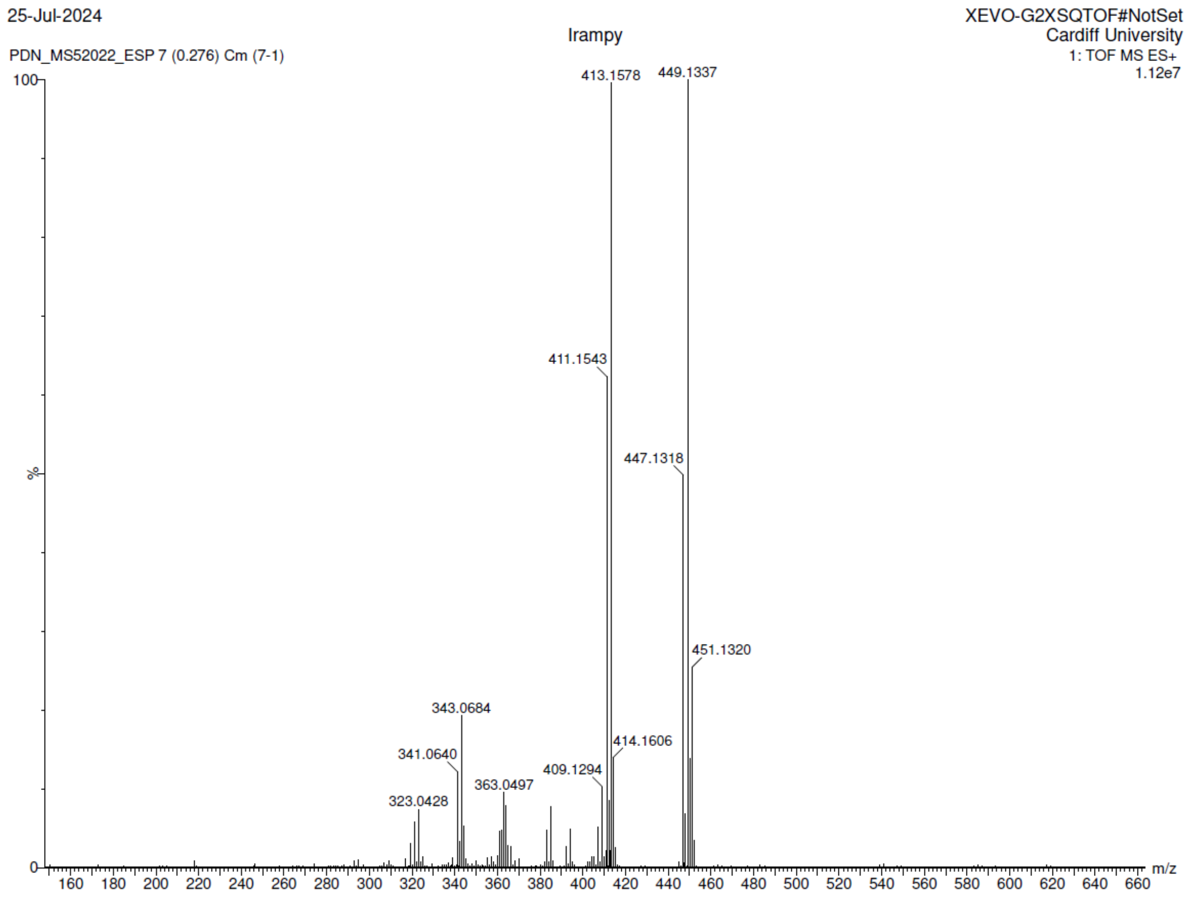

Minimum:				-1.5				
Maximum:		5.0	10.0	50.0				
Mass	Calc. Mass	mDa	PPM	DBE	i-FIT	Norm	Conf(%)	Formula
449.1337	449.1335	0.2	0.4	4.0	1209.4	n/a	n/a	C14 H25 N2 Cl Ir

Figure S9 HRMS of *R,S*-[(η^5-C_5Me_5)Ir(*S*-amp)Cl]PF_6.

NMR and mass spectra of *R,S*-[(η^6-cym)Ru(*S*-amp)Cl]PF_6.

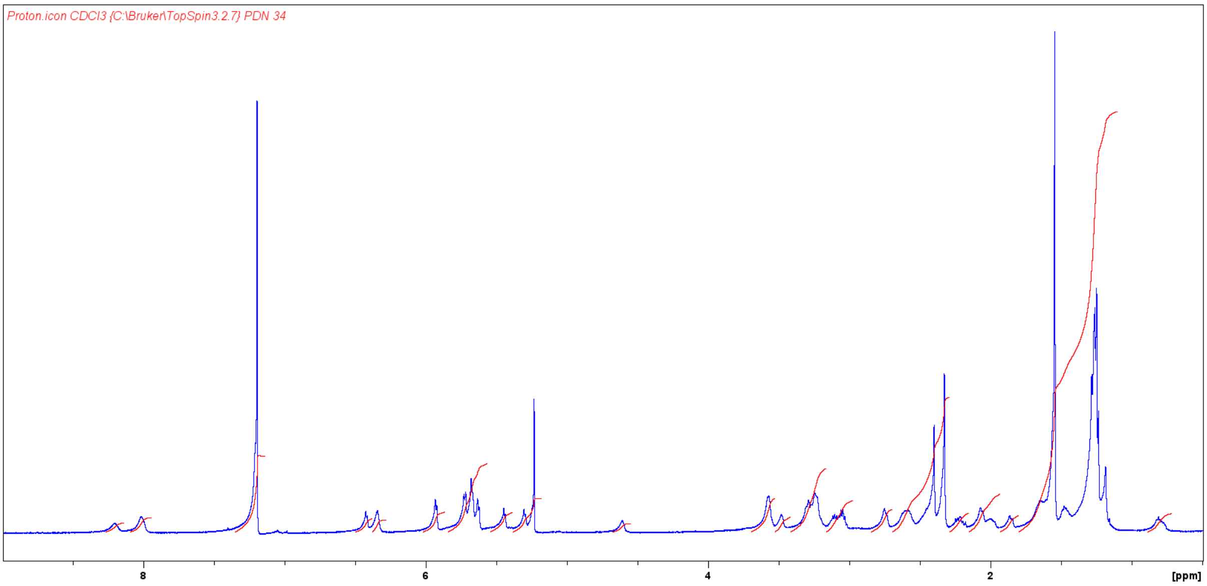

Figure S10 ^{1}H NMR spectrum of *R,S*-[(η^6-cym)Ru(*S*-amp)Cl]PF_6 recorded at 500 MHz in $CDCl_3$.

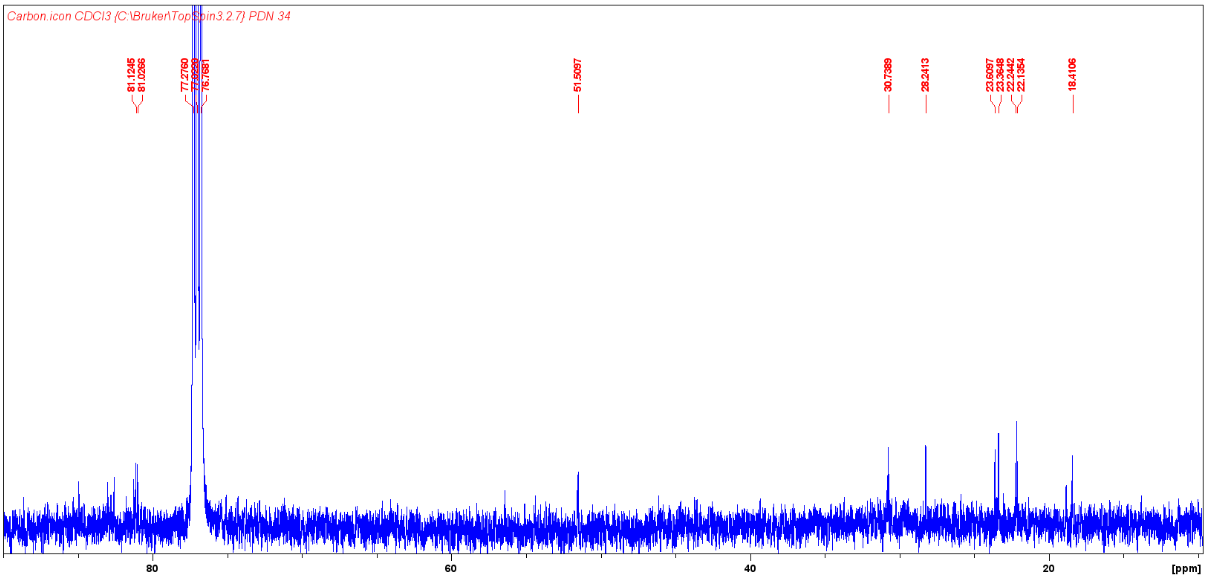

Figure S11 $^{13}C\{^1H\}$ NMR spectrum of *R,S*-[(η^6-cym)Ru(*S*-amp)Cl]PF_6 recorded at 125 MHz in $CDCl_3$.

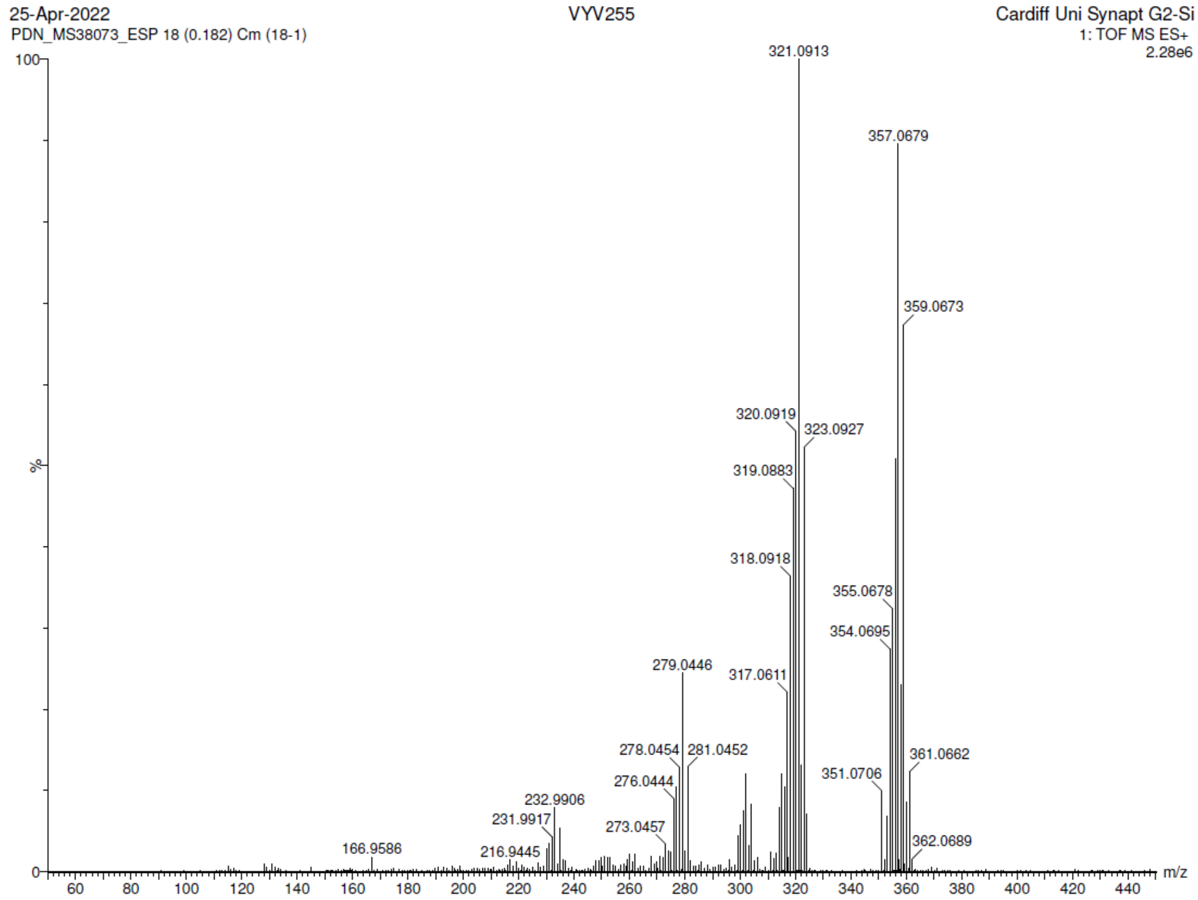

Monoisotopic Mass, Odd and Even Electron Ions
11 formula(e) evaluated with 1 results within limits (up to 50 closest results for each mass)
Elements Used:
C: 0-14 H: 0-24 N: 0-2 Cl: 0-1 102Ru: 0-1

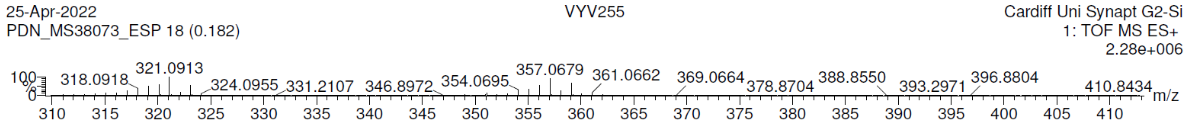

Minimum:				-1.5				
Maximum:		20.0	5.0	50.0				
Mass	Calc. Mass	mDa	PPM	DBE	i-FIT	Norm	Conf(%)	Formula
357.0679	357.0672	0.7	2.0	3.5	978.8	n/a	n/a	C14 H24 N2 Cl 102Ru

Figure S12 HRMS of *R,S*-[(η^6-cym)Ru(*S*-amp)Cl]PF_6.

NMR and mass spectra of [(η^5-C_5Me_5)Rh(*R*-apip)Cl]Cl.

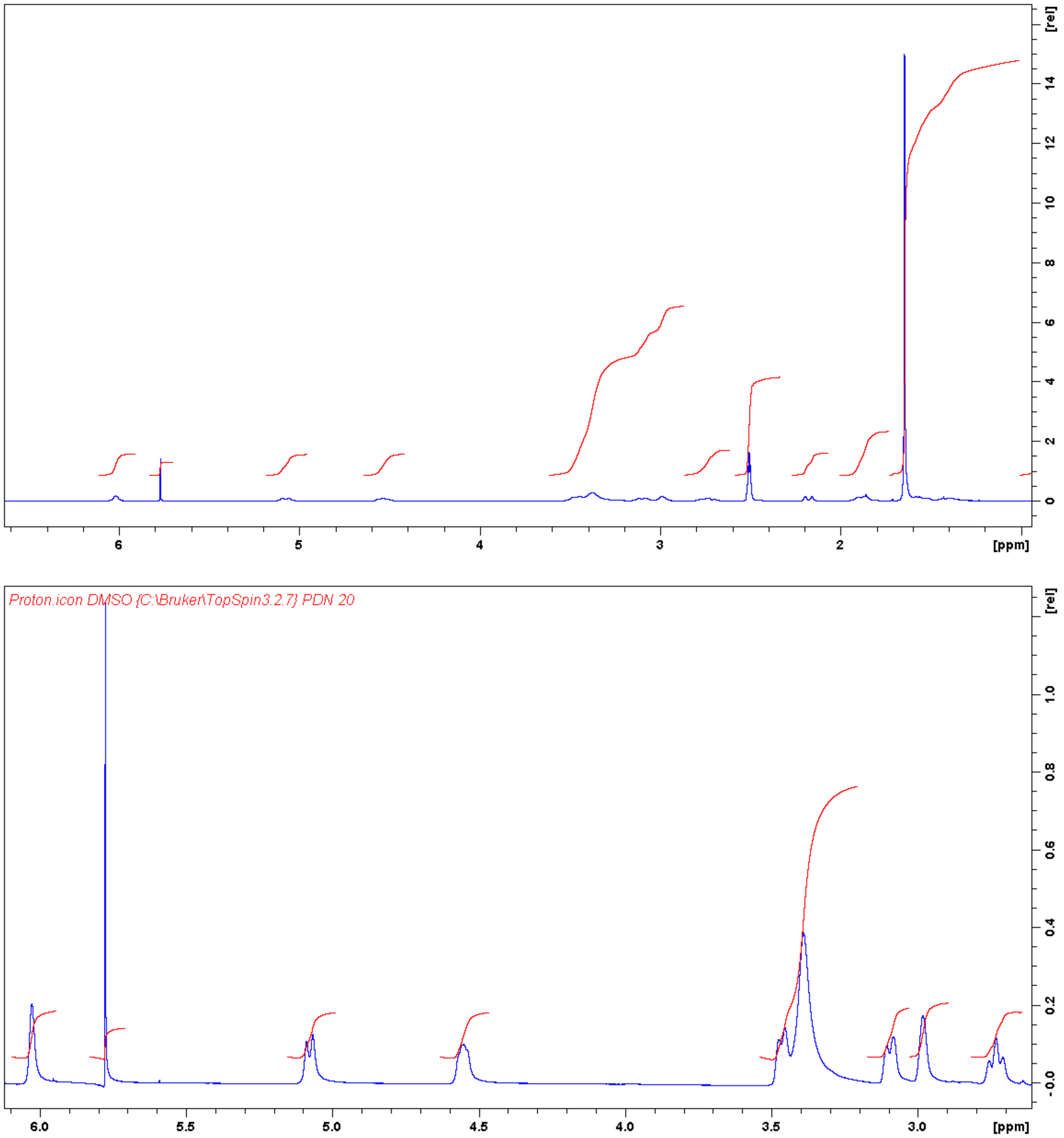

Figure S13 ^{1}H NMR spectra of [(η^5-C_5Me_5)Rh(*R*-apip)Cl]Cl recorded at 300 MHz in D_6-dmso.

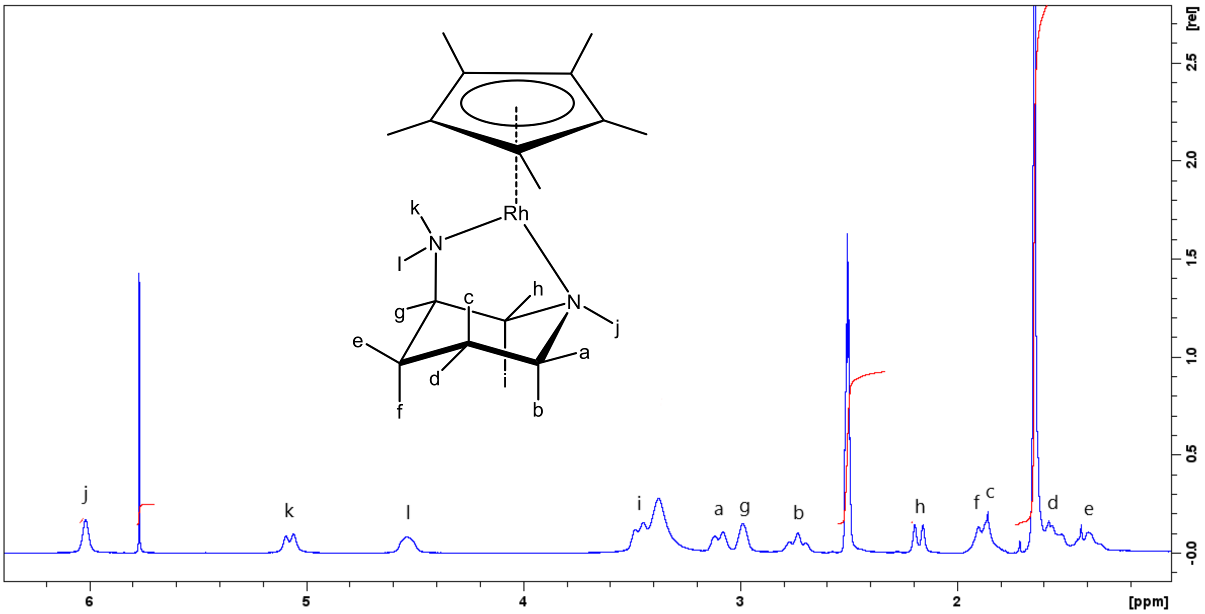

Figure S14 Labelled ^{1}H NMR spectrum of [(η^5-C_5Me_5)Rh(*R*-apip)Cl]Cl recorded at 300 MHz in D_6-dmso.

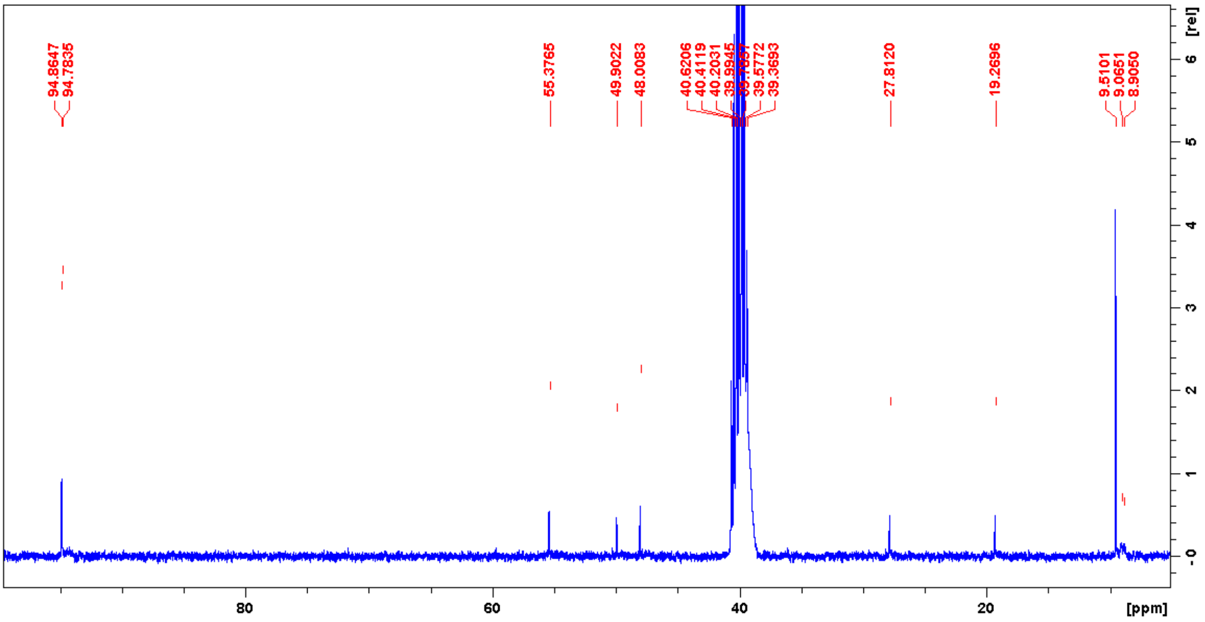

Figure S15 $^{13}C\{^1H\}$ NMR spectrum of [(η^5-C_5Me_5)Rh(*R*-apip)Cl]Cl recorded at 100 MHz in D_6-dmso.

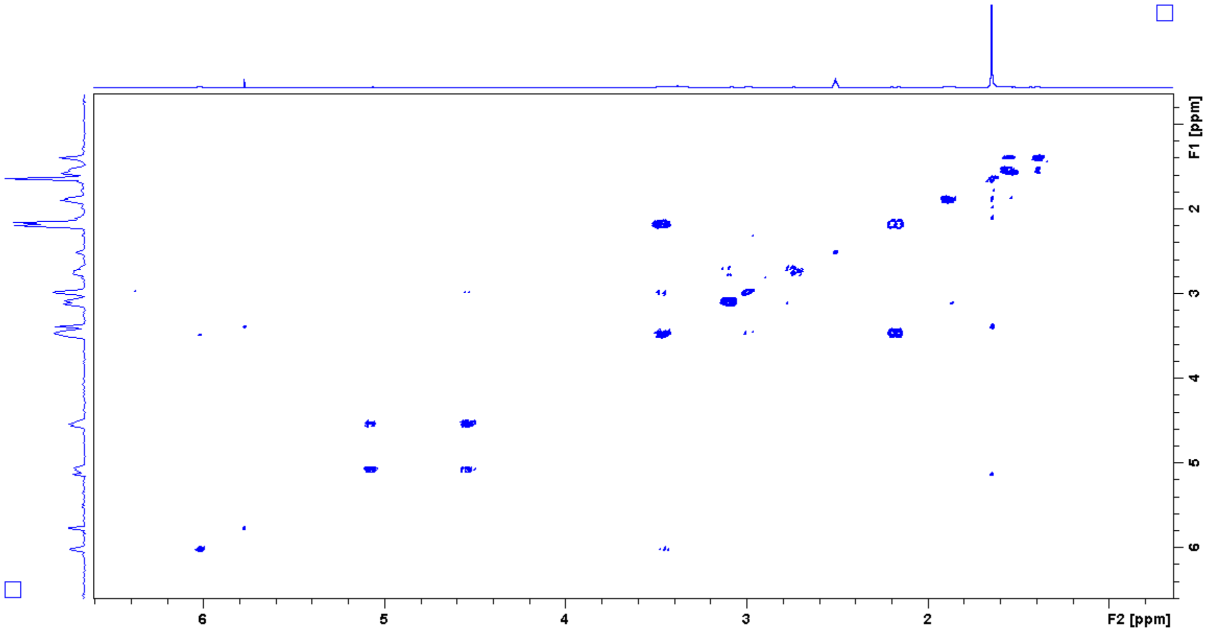

Figure S16 ^1H-^1H COSY NMR spectrum of [(η^5-C_5Me_5)Rh(*R*-apip)Cl]Cl recorded at 100 MHz in D_6-dmso.

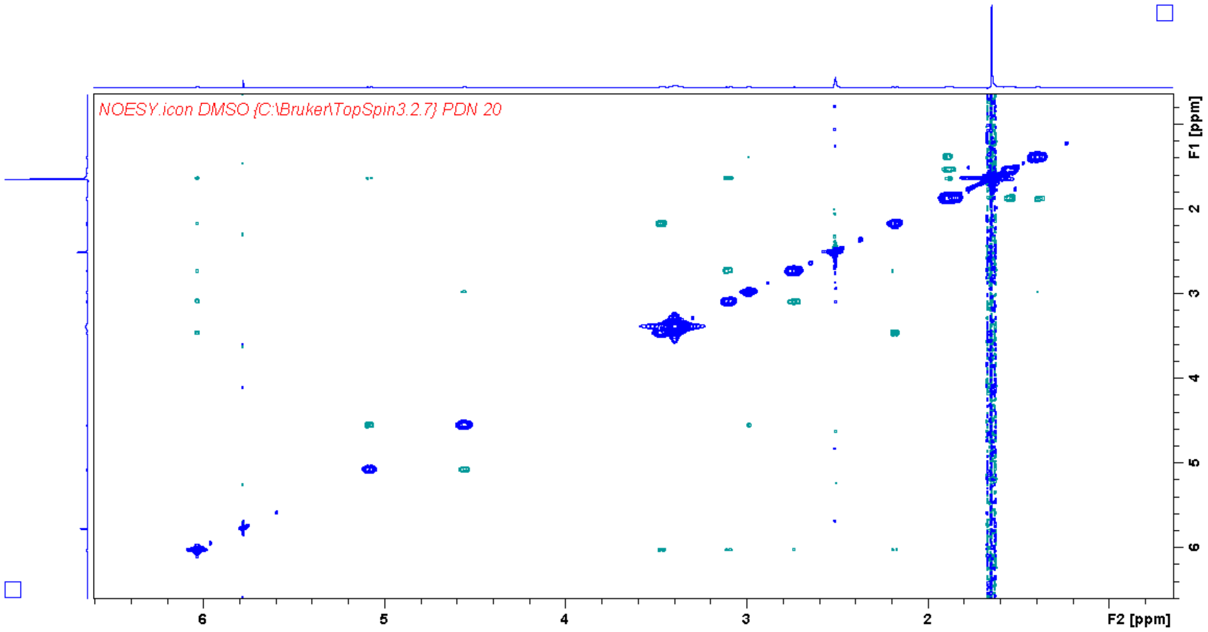

Figure S17 ^1H-^1H NOESY NMR spectrum of [(η^5-C_5Me_5)Rh(*R*-apip)Cl]Cl recorded at 100 MHz in D_6-dmso.

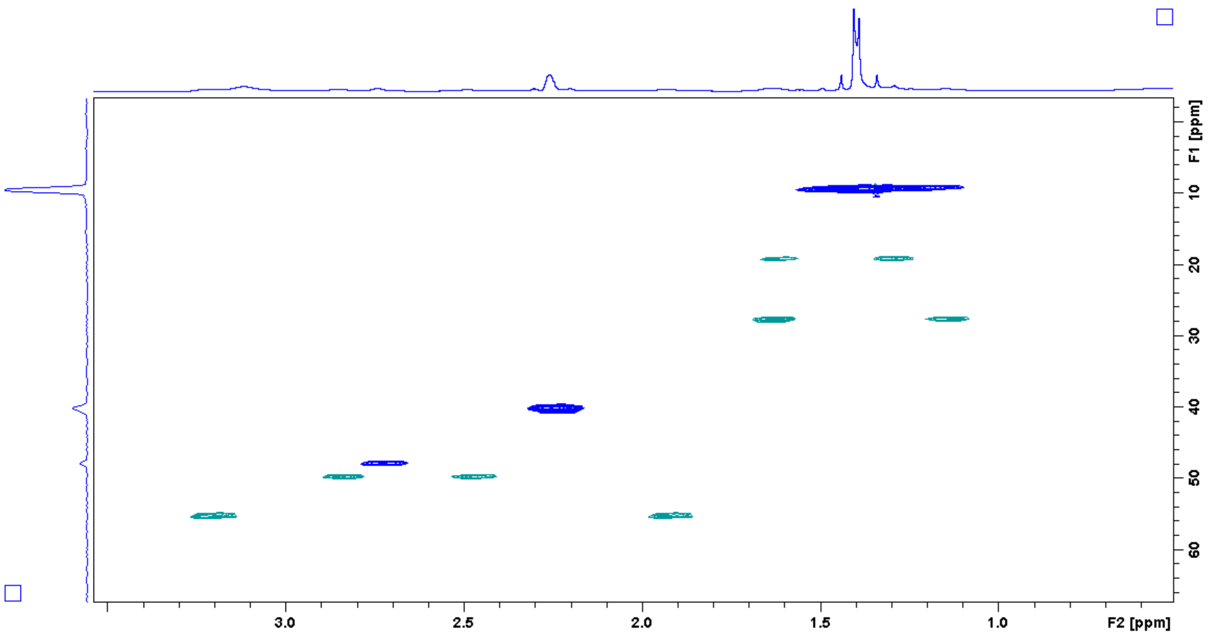

Figure S18 ^{13}C-^{1}H HSQC NMR spectrum of [(η^5-C_5Me_5)Rh(*R*-apip)Cl]Cl recorded at 100 MHz in D_6-dmso.

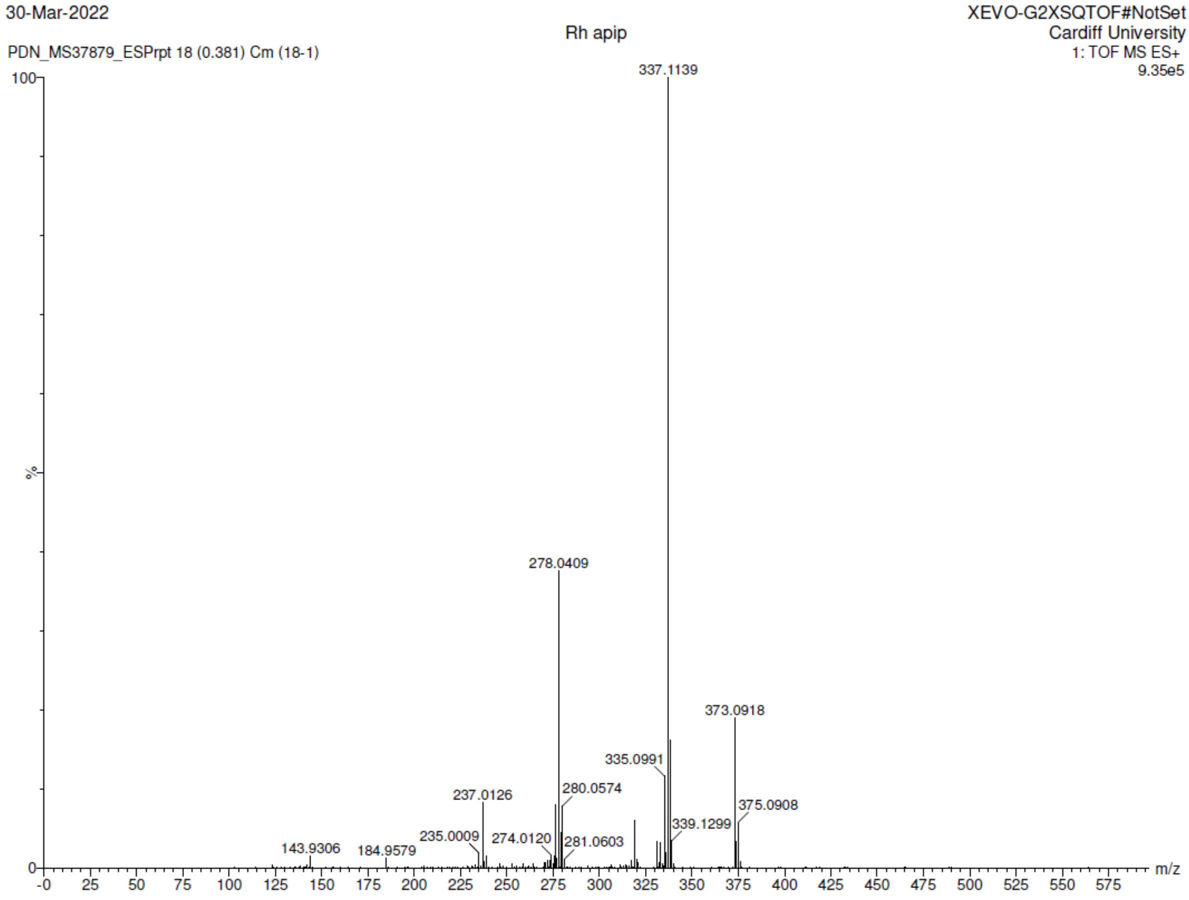

Minimum:				-1.5				
Maximum:		5.0	5.0	100.0				
Mass	Calc. Mass	mDa	PPM	DBE	i-FIT	Norm	Conf(%)	Formula
373.0918	373.0918	0.0	0.0	3.0	301.3	n/a	n/a	C15 H27 N2 Cl 103Rh

Figure S19 HRMS (ES^+) spectrum of [(η^5-C_5Me_5)Rh(*R*-apip)Cl]Cl.

NMR and mass spectra of [(η^5-C_5Me_5)Ir(*R*-apip)Cl]Cl.

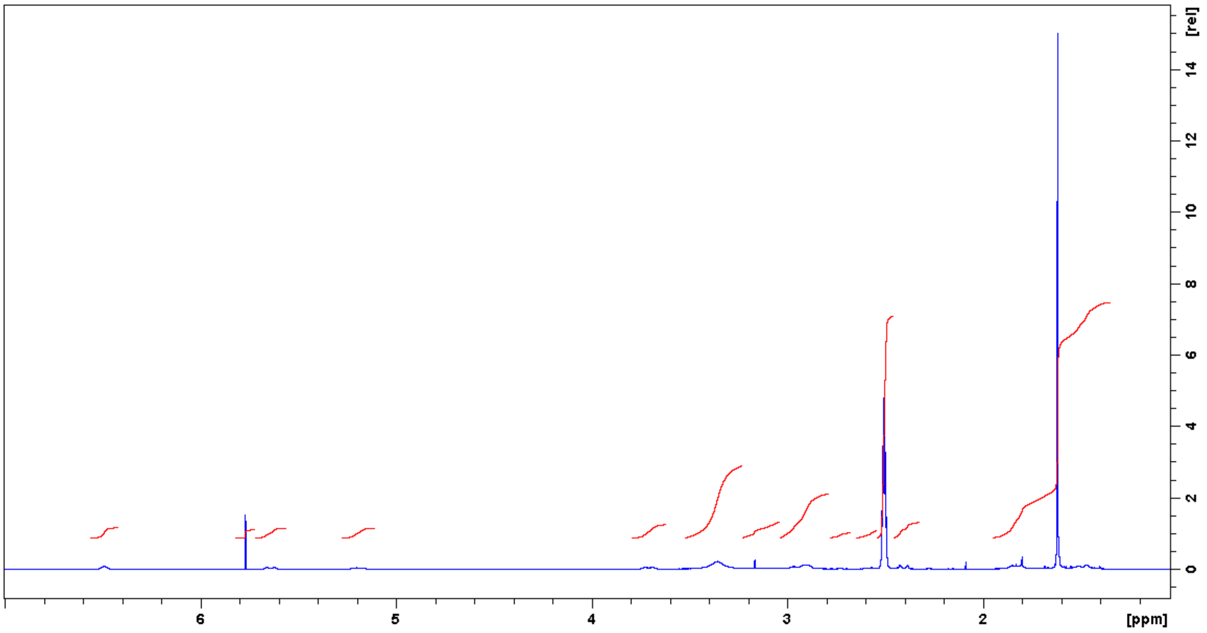

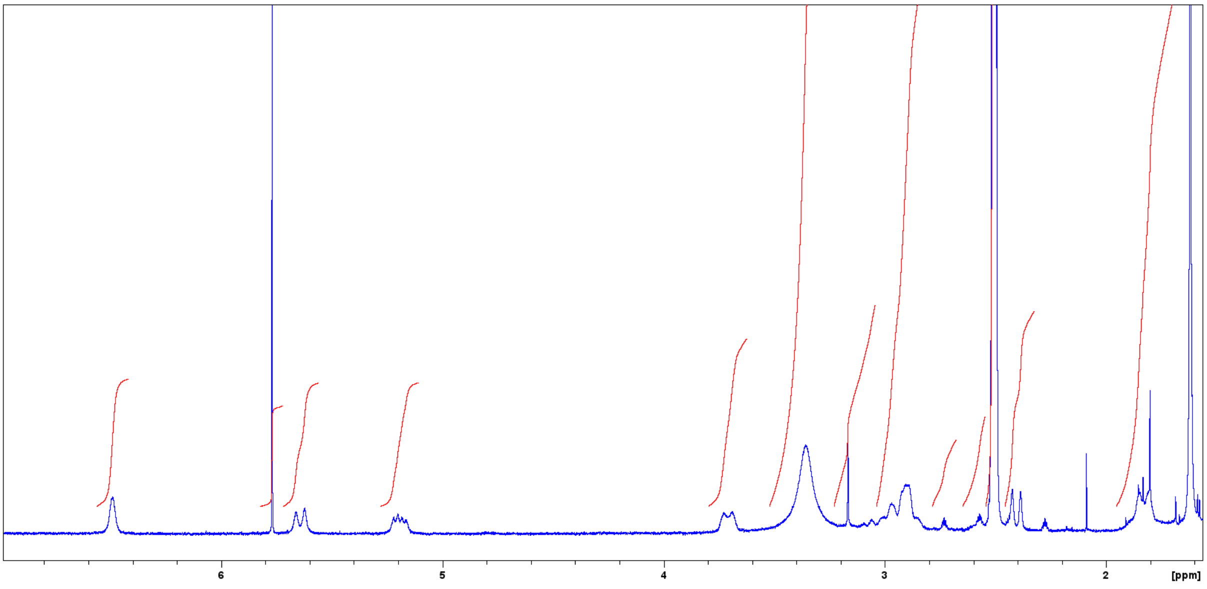

Figure S20 ^1H NMR spectra of [(η^5-C_5Me_5)Ir(*R*-apip)Cl]Cl recorded at 300 MHz in D_6-dmso.

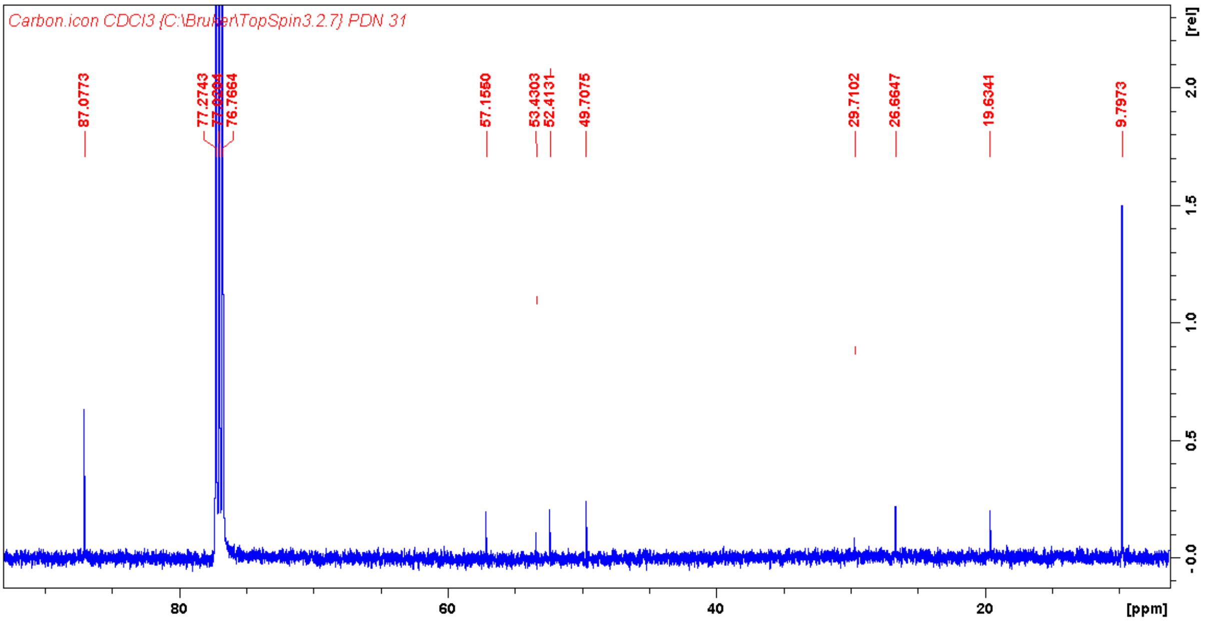

Figure S21 $^{13}C\{^{1}H\}$ NMR spectrum of $[(\eta^5\text{-}C_5Me_5)Ir(R\text{-apip})Cl]Cl$ recorded at 100 MHz in D_6-dmso.

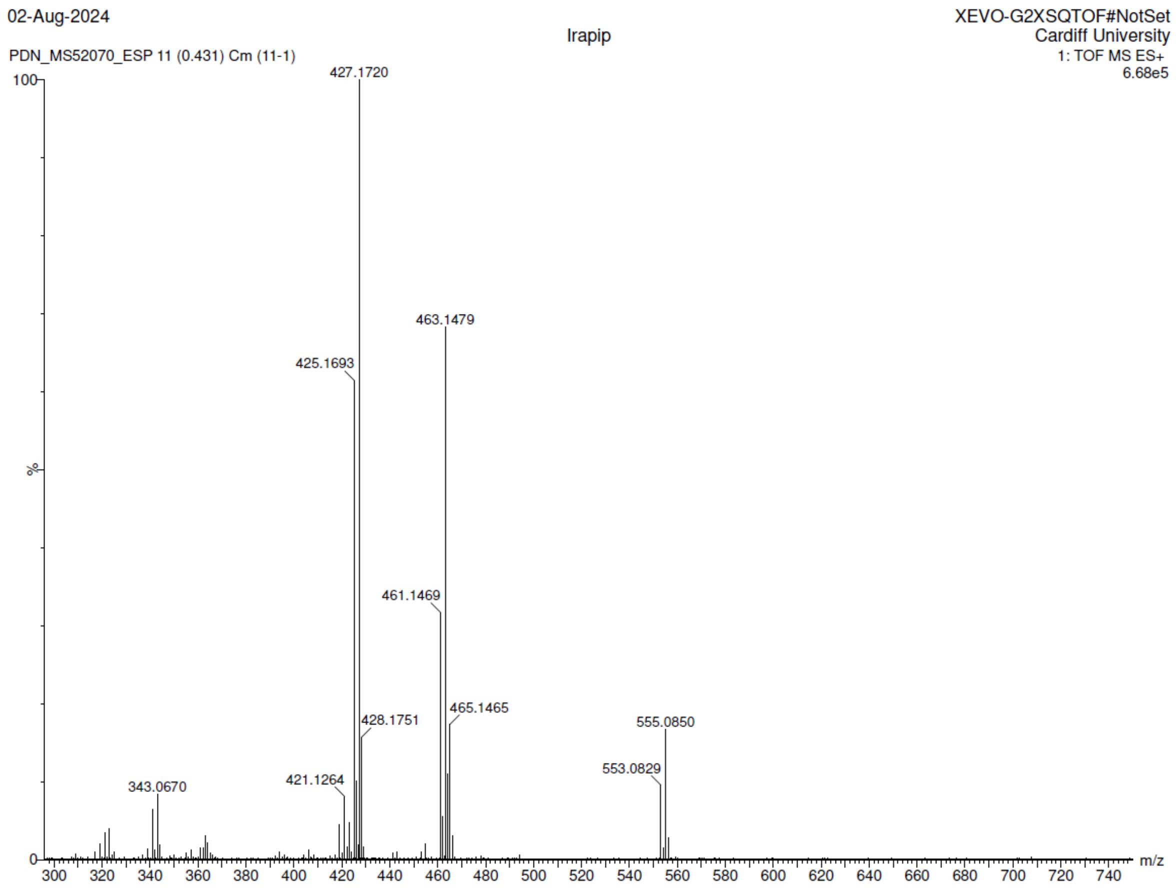

	Mass	Calc. Mass	mDa	PPM	DBE	i-FIT	Norm	Conf(%)	Formula
Minimum:					-1.5				
Maximum:			5.0	10.0	50.0				
	463.1479	463.1492	-1.3	-2.8	4.0	778.6	n/a	n/a	C15 H27 N2 Cl Ir

Figure S22 HRMS (ES$^+$) spectrum of [(η^5-C_5Me_5)Ir(*R*-apip)Cl]Cl.

NMR and mass spectra of *R,S*-[(η^6-cym)Ru(*R*-apip)Cl]PF_6.

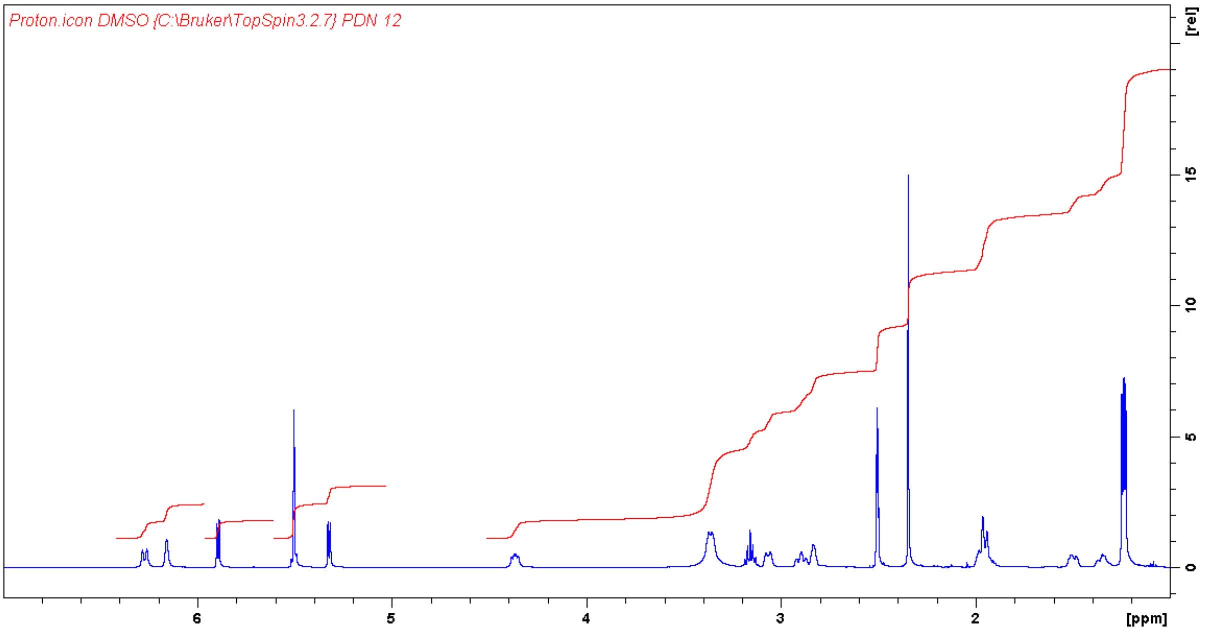

Figure S23 ^{1}H NMR spectrum of *R,S*-[(η^6-cym)Ru(*S*-apip)Cl]PF_6 recorded at 500 MHz in d_6-DMSO.

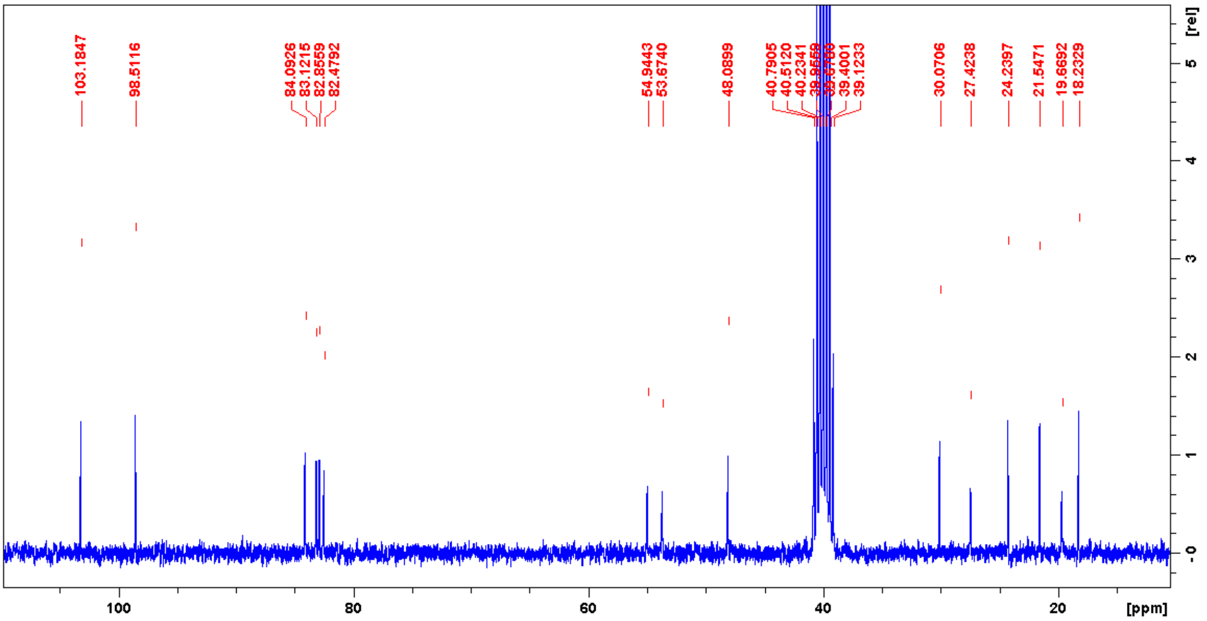

Figure S24 ^{13}C{^{1}H} NMR spectrum of *R,S*-[(η^6-cym)Ru(*S*-apip)Cl]PF_6 recorded at 500 MHz in d_6-DMSO.

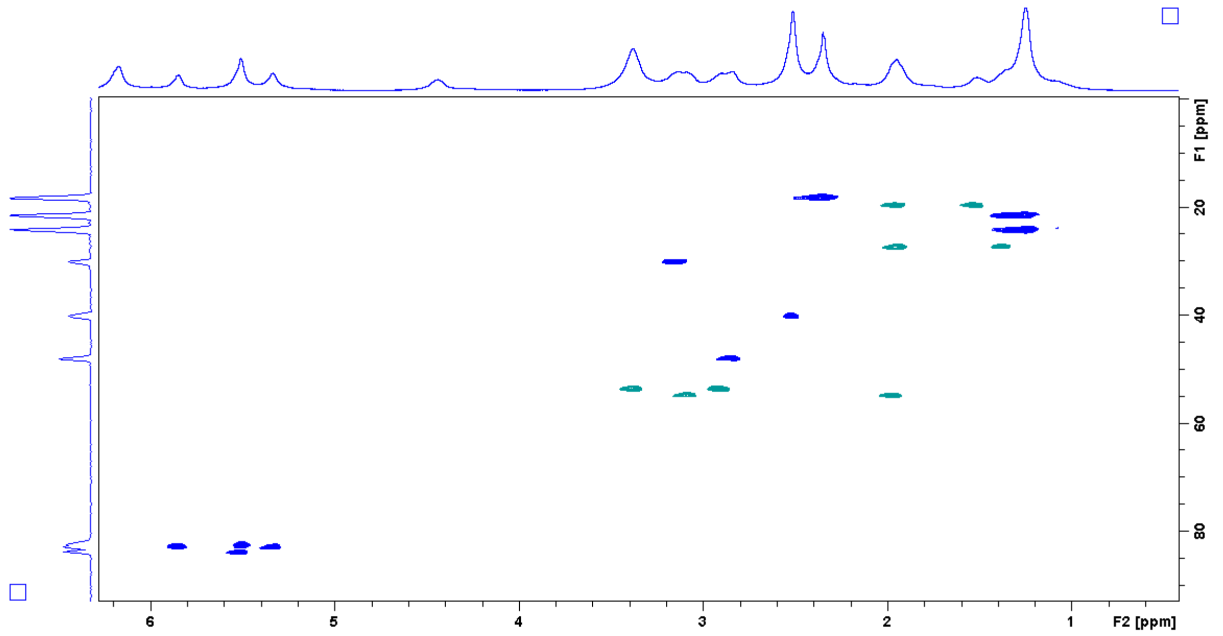

Figure S25 HSQC NMR spectrum of *R,S*-[(η^6-cym)Ru(*S*-apip)Cl]PF_6 recorded at 500 MHz in d_6-DMSO.

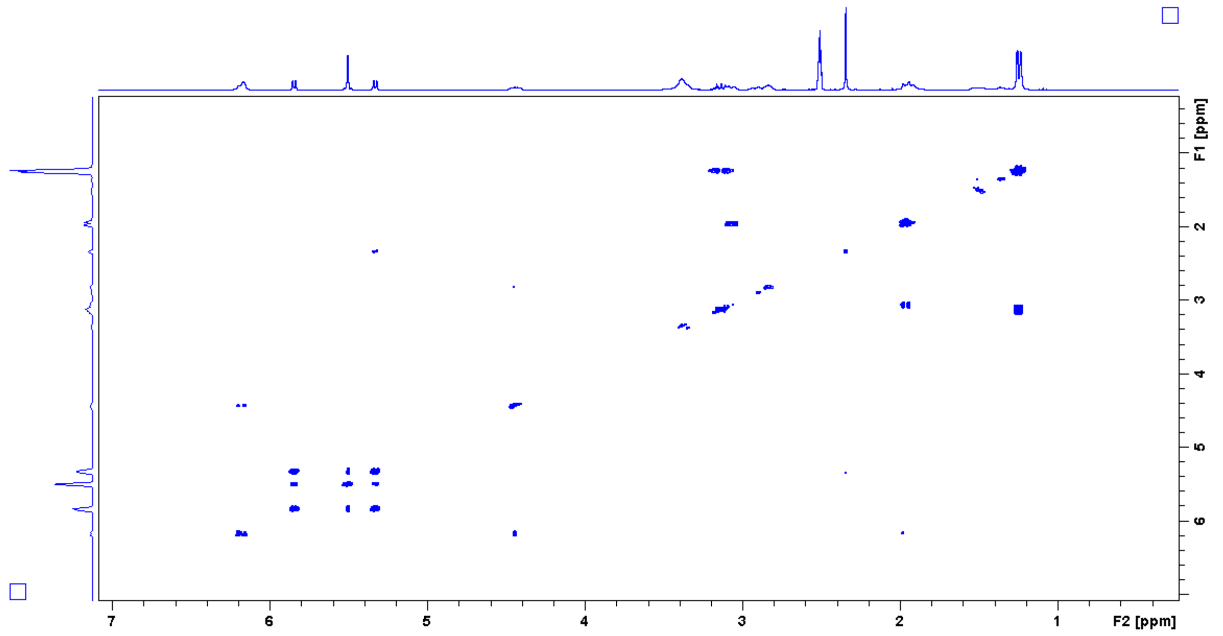

Figure S26 ^{1}H-^{1}H COSY NMR spectrum of *R,S*-[(η^6-cym)Ru(*S*-apip)Cl]PF_6 recorded at 500 MHz in d_6-DMSO.

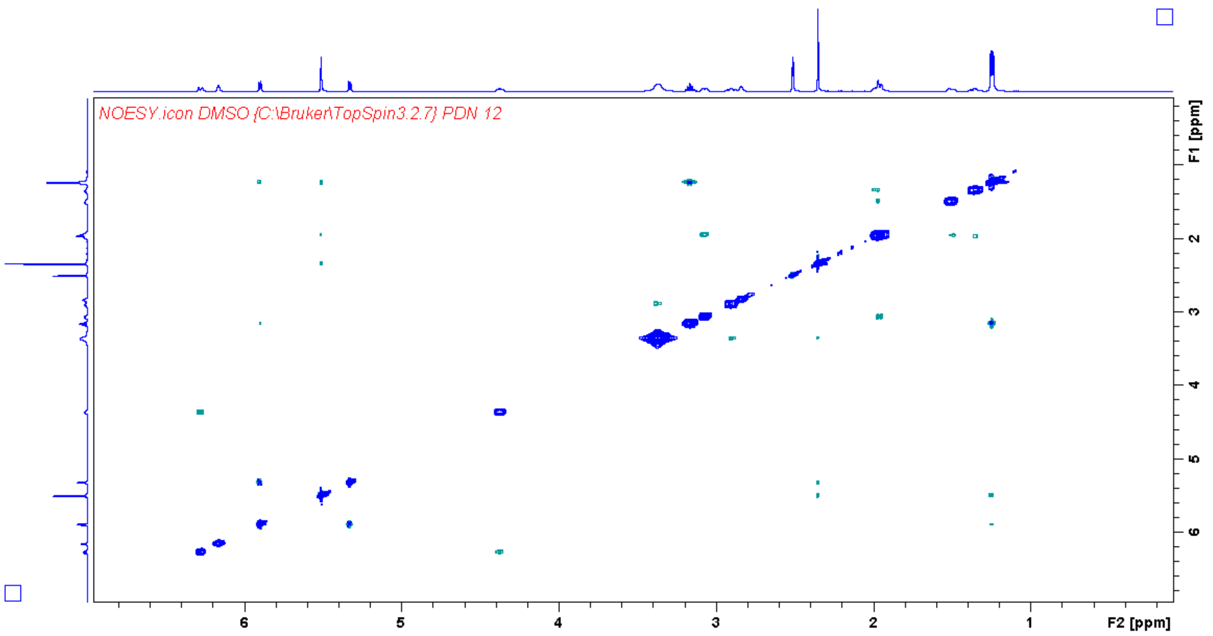

Figure S27 ^{1}H-^{1}H NOESY NMR spectrum of *R,S*-[(η^{6}-cym)Ru(*S*-apip)Cl]PF_6 recorded at 500 MHz in d_6-DMSO.

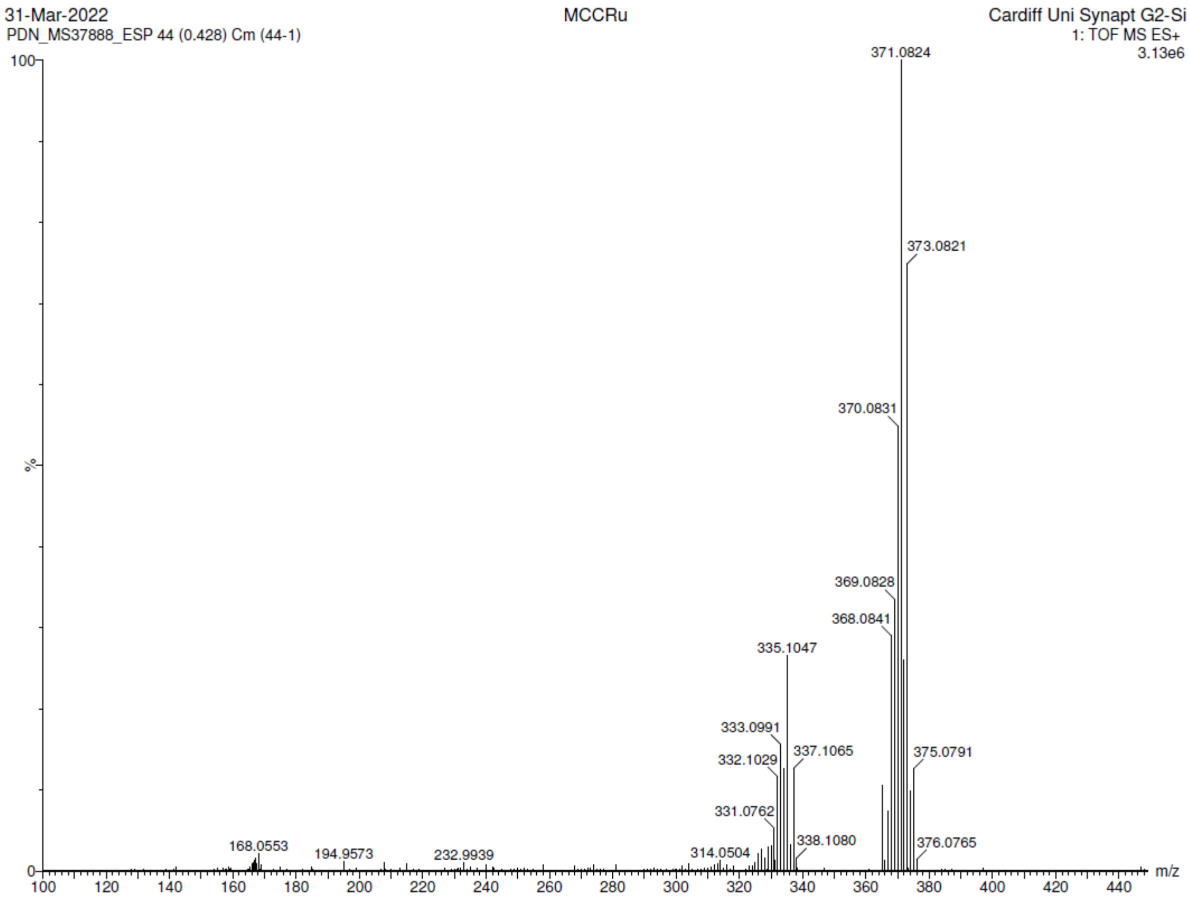

Monoisotopic Mass, Even Electron Ions
87 formula(e) evaluated with 1 results within limits (up to 50 closest results for each mass)
Elements Used:
C: 0-15   H: 0-26   N: 0-2   O: 0-5   Cl: 0-1   Ru: 0-1

31-Mar-2022 MCCRu Cardiff Uni Synapt G2-Si
PDN_MS37888_ESP 44 (0.428) Cm (44-1) 1: TOF MS ES+
3.13e+006

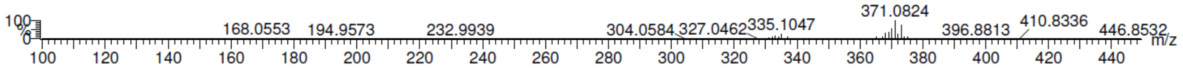

Minimum:				-1.5				
Maximum:		20.0	5.0	50.0				
Mass	Calc. Mass	mDa	PPM	DBE	i-FIT	Norm	Conf(%)	Formula
371.0824	371.0828	-0.4	-1.1	3.5	996.6	n/a	n/a	C15 H26 N2 Cl Ru

Figure S28 HRMS of *R,S*-[(η[6]-cym)Ru(*S*-apip)Cl]PF_6 recorded at 500 MHz in d_6-DMSO.

NMR and mass spectra of [(η[5]-C_5Me_5)Rh(*R*-ahaz)Cl]PF_6.

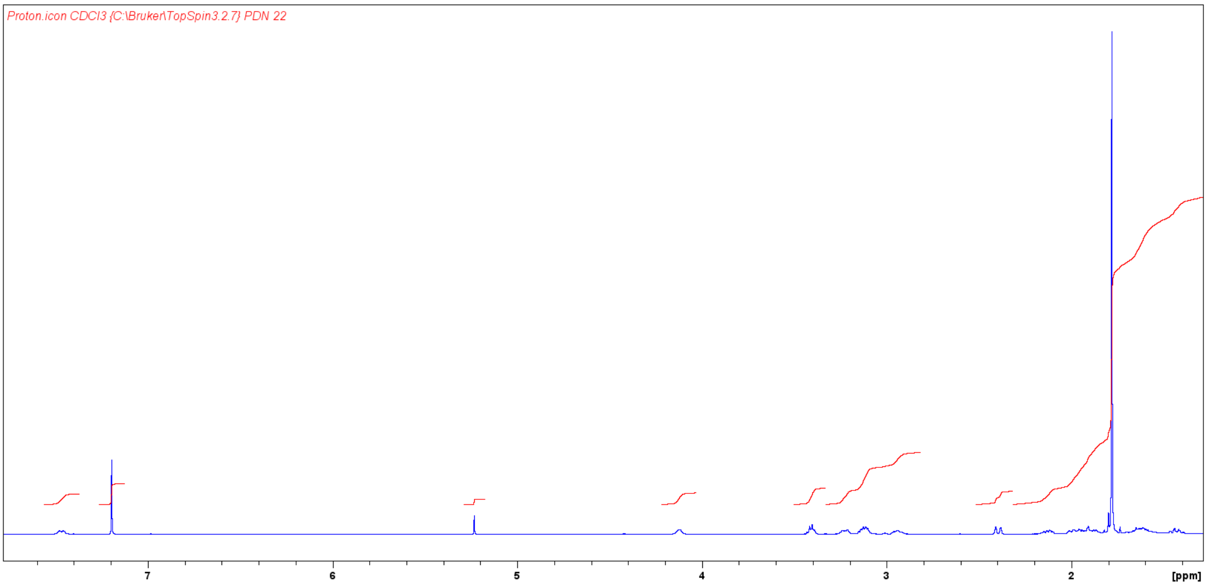

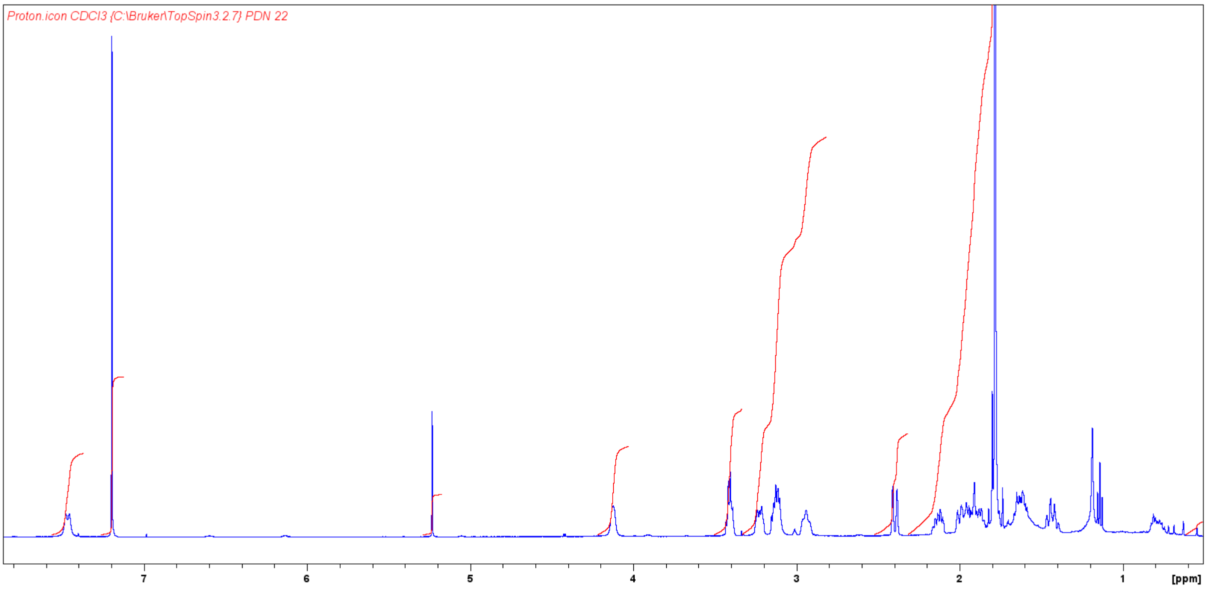

Figure S29 ^{1}H NMR spectrum of [(η[5]-C_5Me_5)Rh(*R*-ahaz)Cl]PF_6 recorded at 500 MHz in $CDCl_3$.

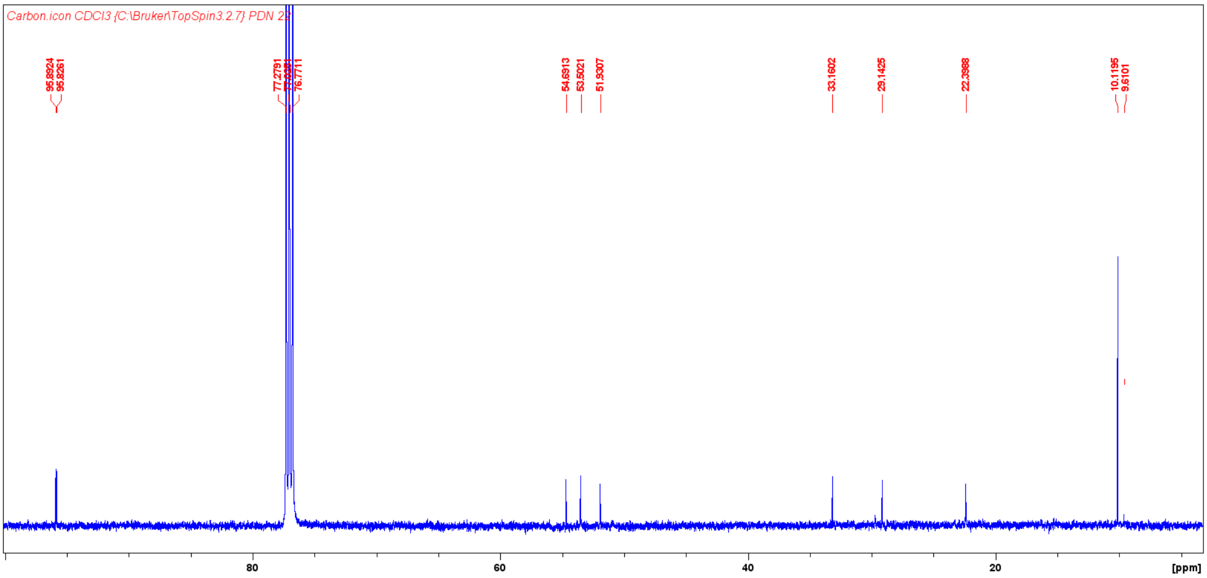

Figure S30 $^{13}C\{^{1}H\}$ NMR spectrum of $[(\eta^5\text{-}C_5Me_5)Rh(R\text{-ahaz})Cl]PF_6$ recorded at 500 MHz in $CDCl_3$.

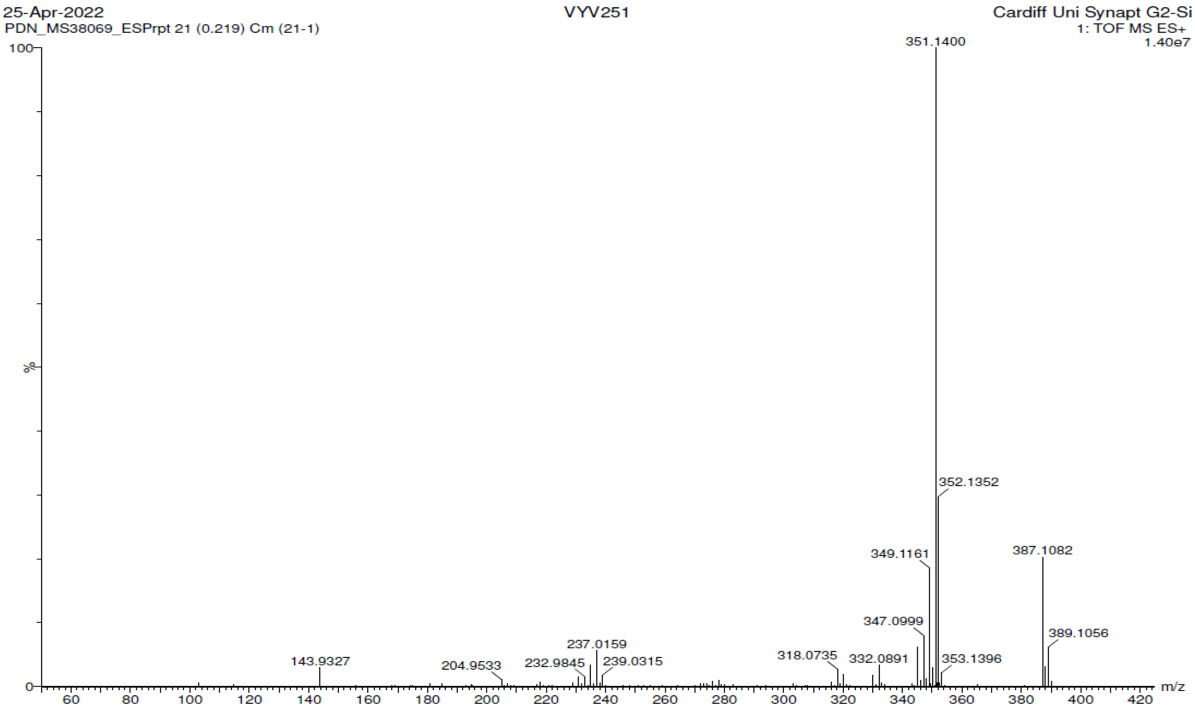

Monoisotopic Mass, Odd and Even Electron Ions
11 formula(e) evaluated with 1 results within limits (up to 50 closest results for each mass)
Elements Used:
C: 0-16   H: 0-29   N: 0-2   Cl: 0-1   103Rh: 0-1

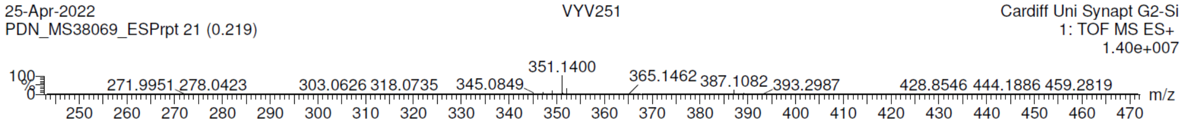

Minimum:				-1.5				
Maximum:		20.0	5.0	50.0				
Mass	Calc. Mass	mDa	PPM	DBE	i-FIT	Norm	Conf(%)	Formula
387.1082	387.1074	0.8	2.1	3.0	950.4	n/a	n/a	C16 H29 N2 Cl 103Rh

Figure S31 HRMS of [(η^5-C_5Me_5)Rh(*R*-ahaz)Cl]PF_6.

NMR and mass spectra of [(η^5-C_5Me_5)Ir(*R*-ahaz)Cl]Cl.

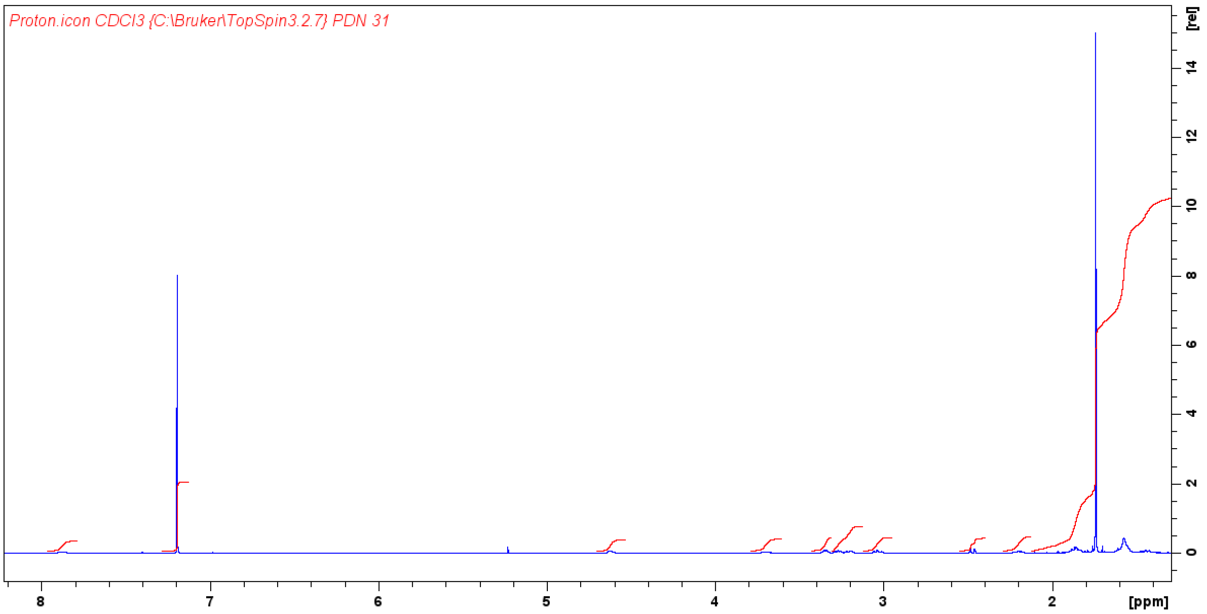

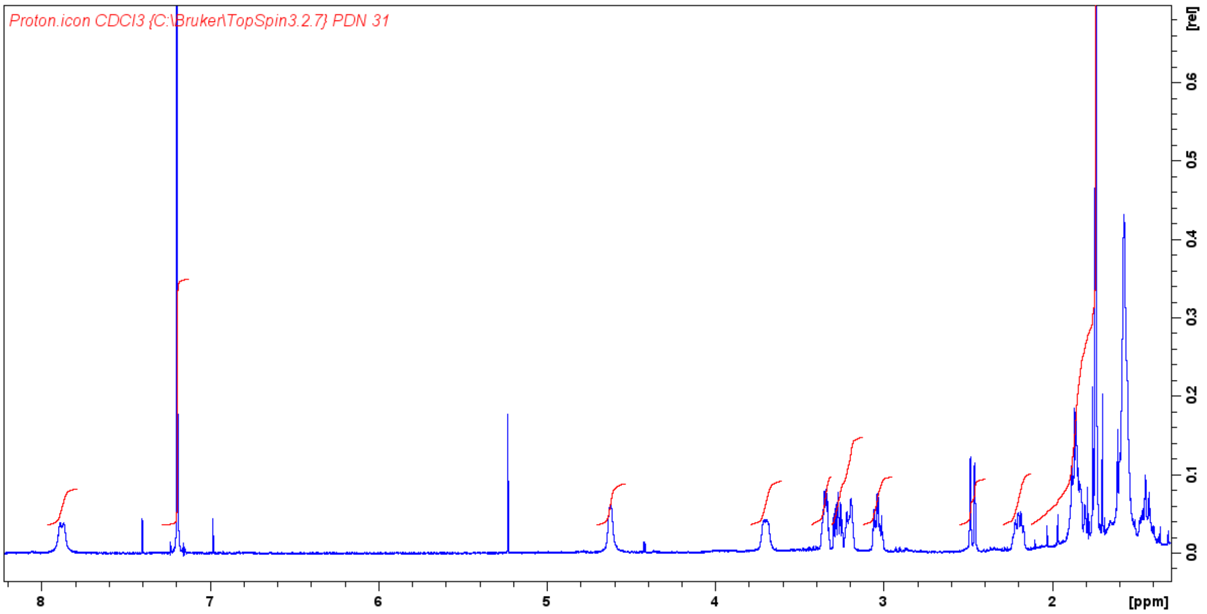

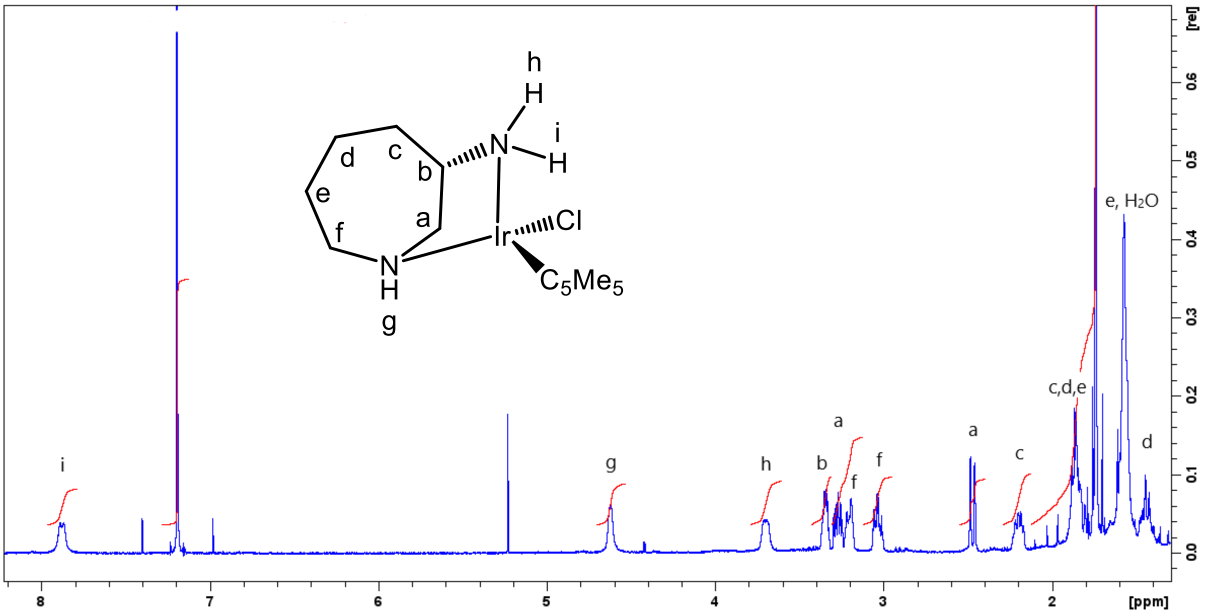

Figure S32 ^{1}H NMR spectra of [(η^5-C_5Me_5)Ir(*R*-ahaz)Cl]Cl recorded at 300 MHz in D_6-dmso.

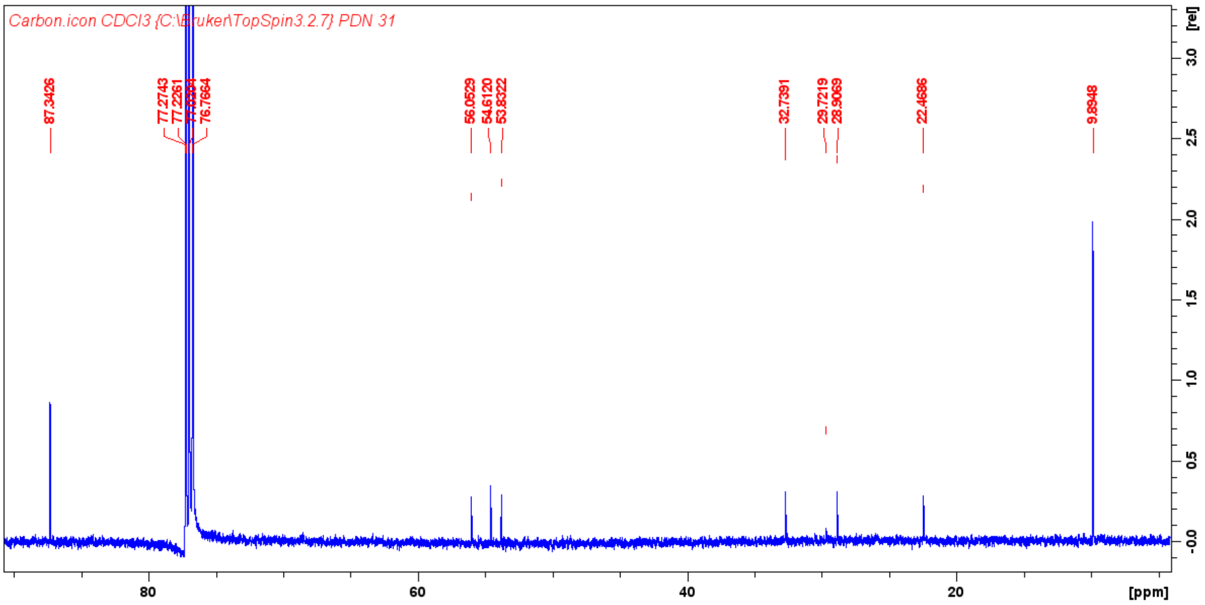

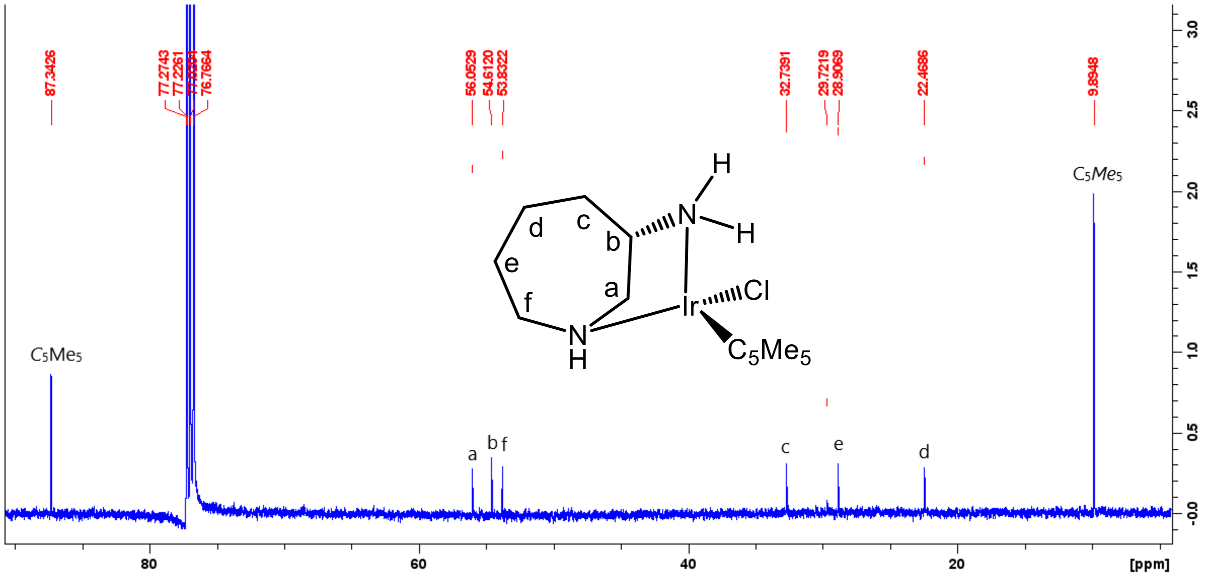

Figure S33 $^{13}C\{^{1}H\}$ NMR spectrum of [(η^5-C_5Me_5)Ir(*R*-ahaz)Cl]Cl recorded at 300 MHz in D_6-dmso.

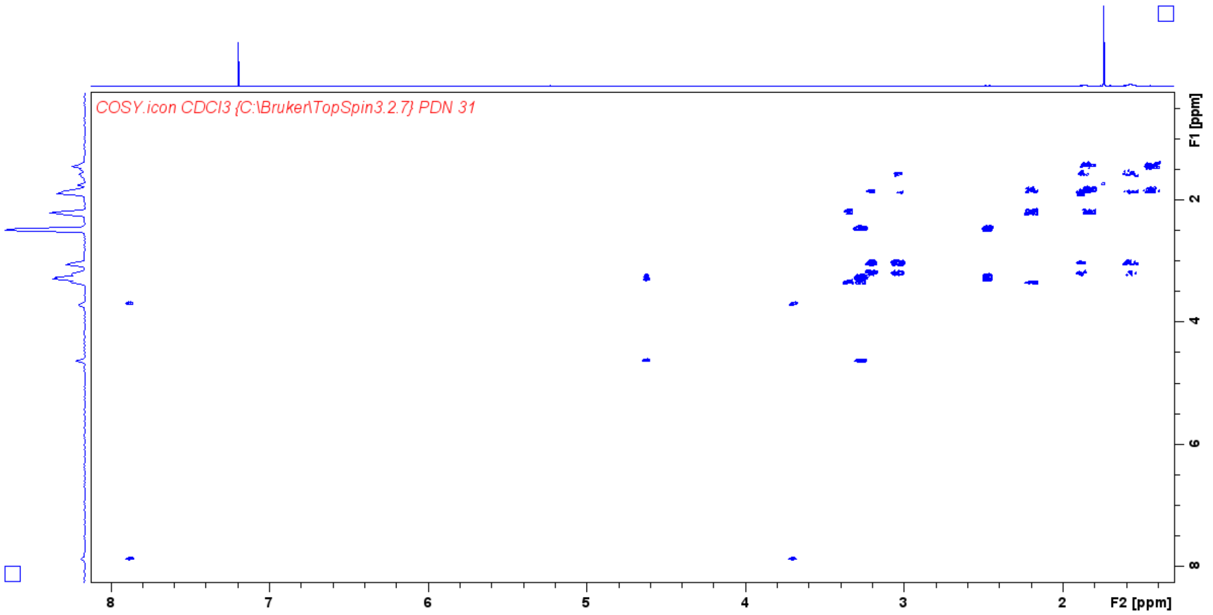

Figure S34 ^{1}H-^{1}H COSY NMR spectrum of [(η^5-C_5Me_5)Ir(*R*-ahaz)Cl]Cl recorded at 300 MHz in D_6-dmso.

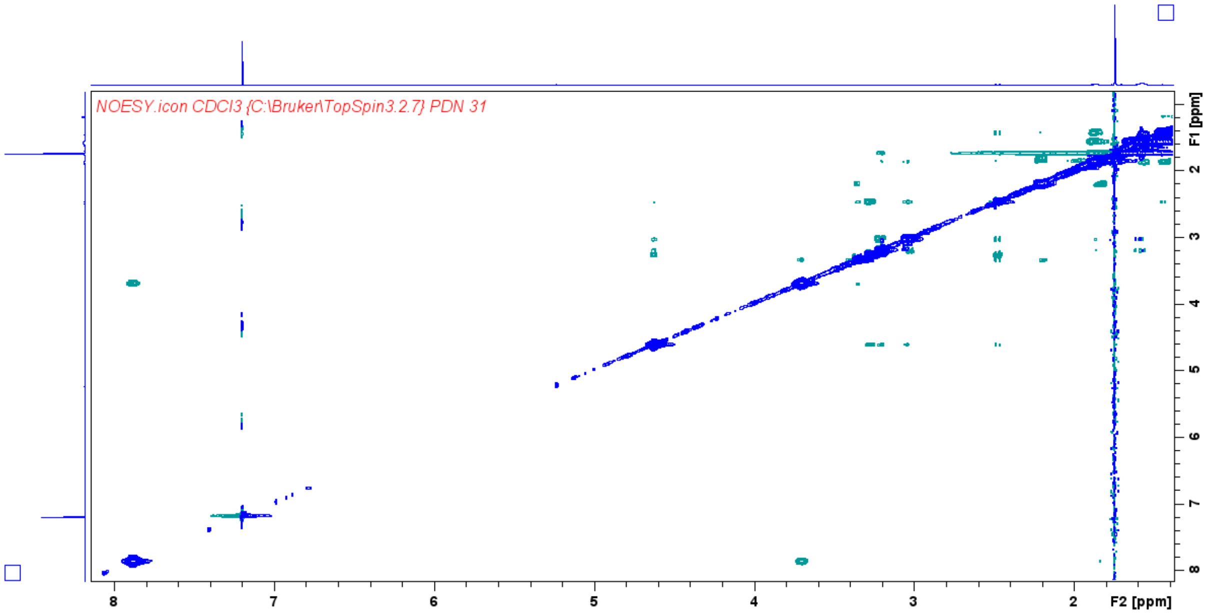

Figure S35 ^{1}H-^{1}H NOESY NMR spectrum of [(η^{5}-C_5Me_5)Ir(*R*-ahaz)Cl]Cl recorded at 300 MHz in D_6-dmso.

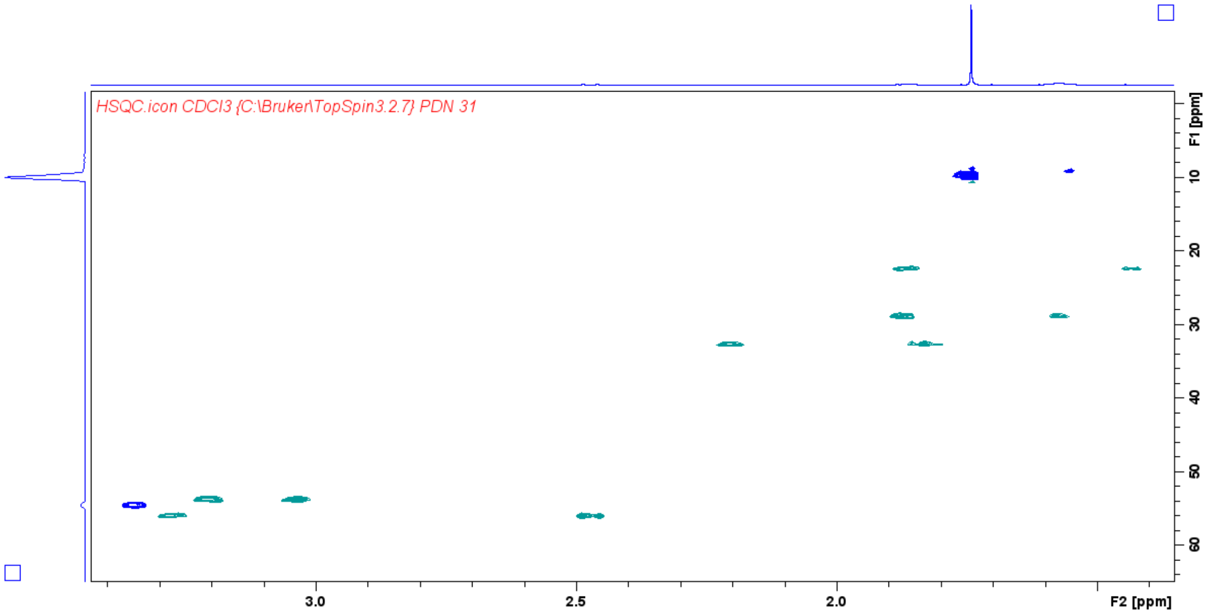

Figure S36 ^{13}C-^{1}H HSQC NMR spectrum of [(η^{5}-C_5Me_5)Ir(*R*-ahaz)Cl]Cl recorded at 300 MHz in D_6-dmso.

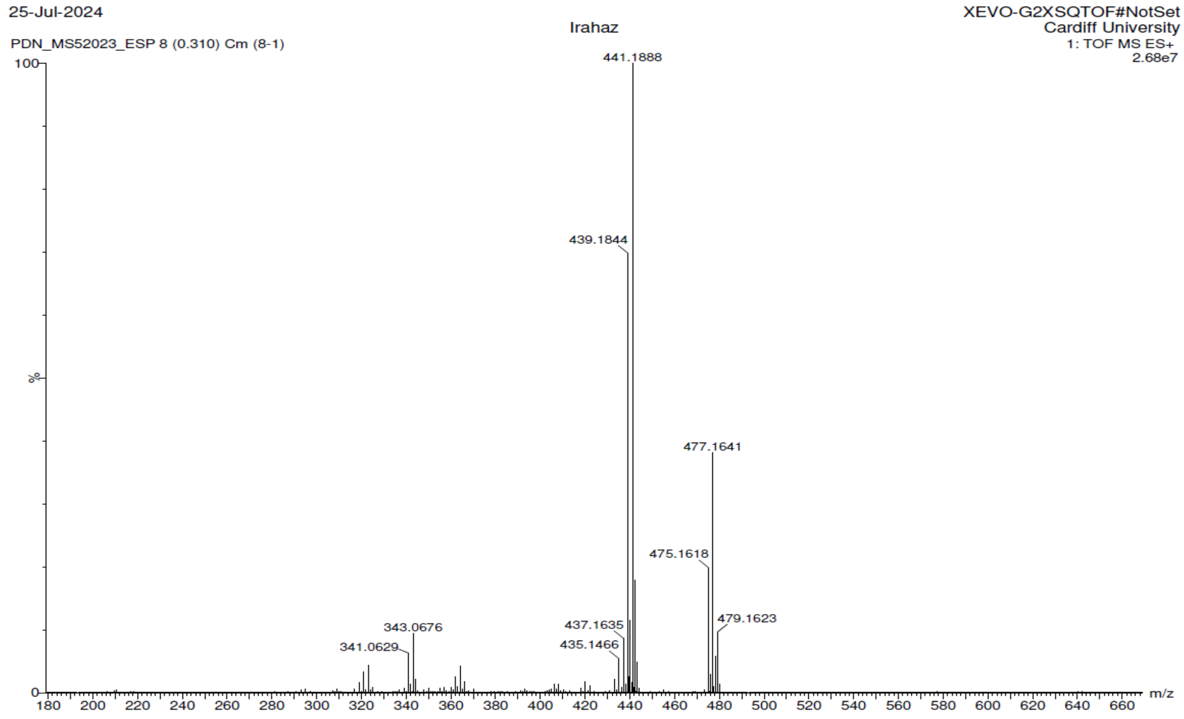

Minimum:				-1.5				
Maximum:		5.0	10.0	50.0				
Mass	Calc. Mass	mDa	PPM	DBE	i-FIT	Norm	Conf(%)	Formula
477.1641	477.1648	-0.7	-1.5	4.0	1018.7	n/a	n/a	C16 H29 N2 Cl Ir

Figure S37 HRMS of [(η^5-C_5Me_5)Ir(*R*-ahaz)Cl]Cl.

NMR and mass spectra of [(η^6-cym)Ru(*R*-ahaz)Cl]Cl.

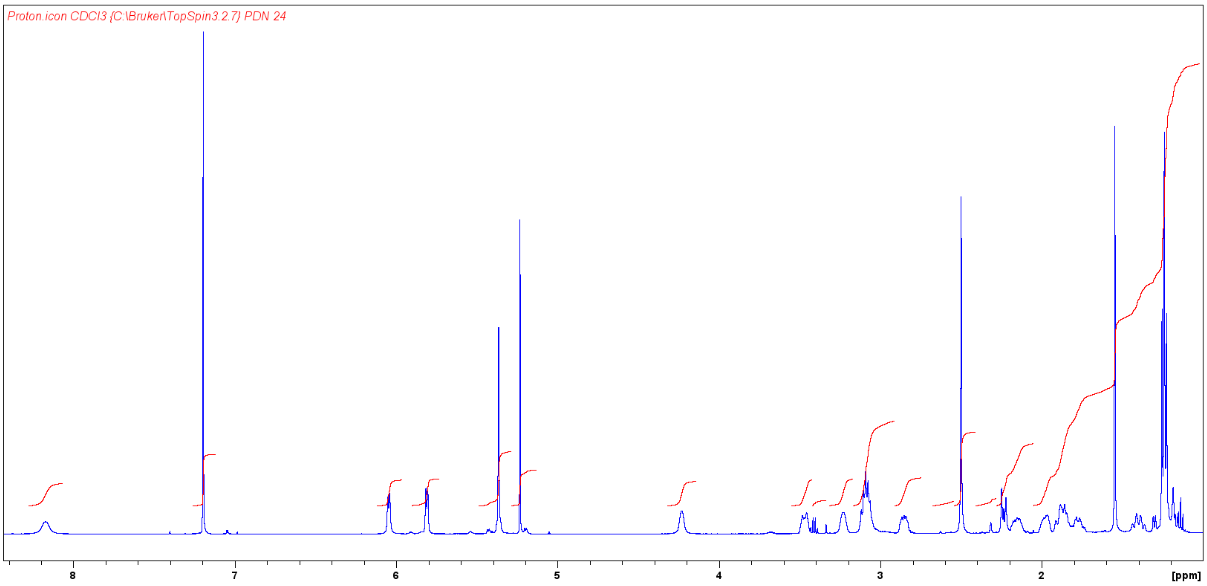

Figure S38 ^{1}H NMR spectrum of [(η^6-cym)Ru(*R*-ahaz)Cl]Cl recorded at 300 MHz in D_6-dmso.

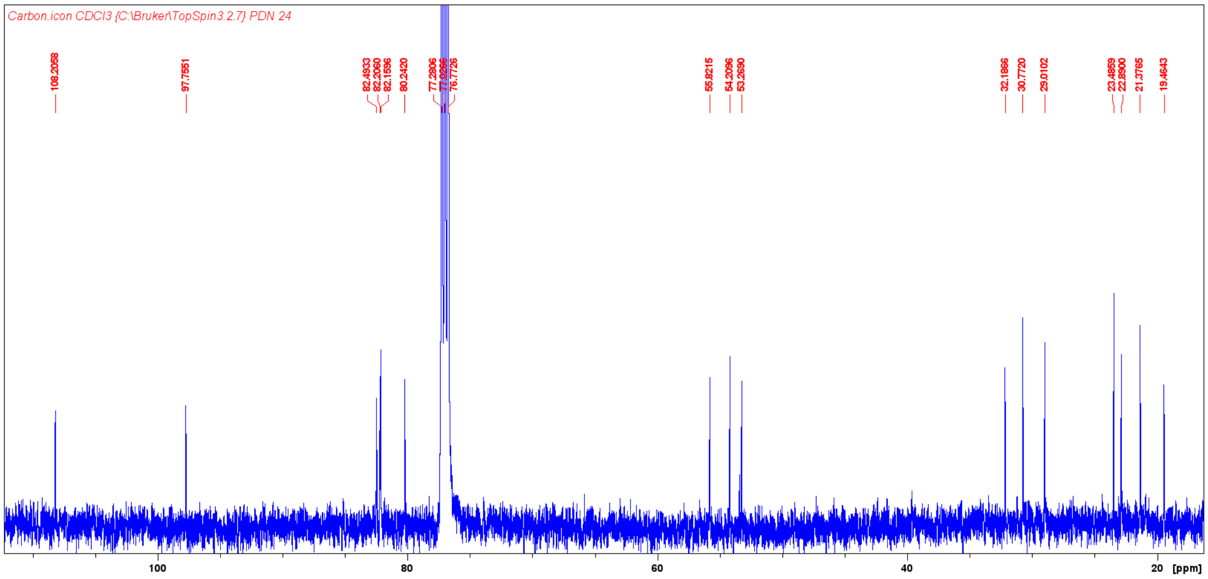

Figure S39 $^{13}C\{^{1}H\}$ NMR spectrum of [(η^6-cym)Ru(*R*-ahaz)Cl]Cl recorded at 300 MHz in D_6-dmso.

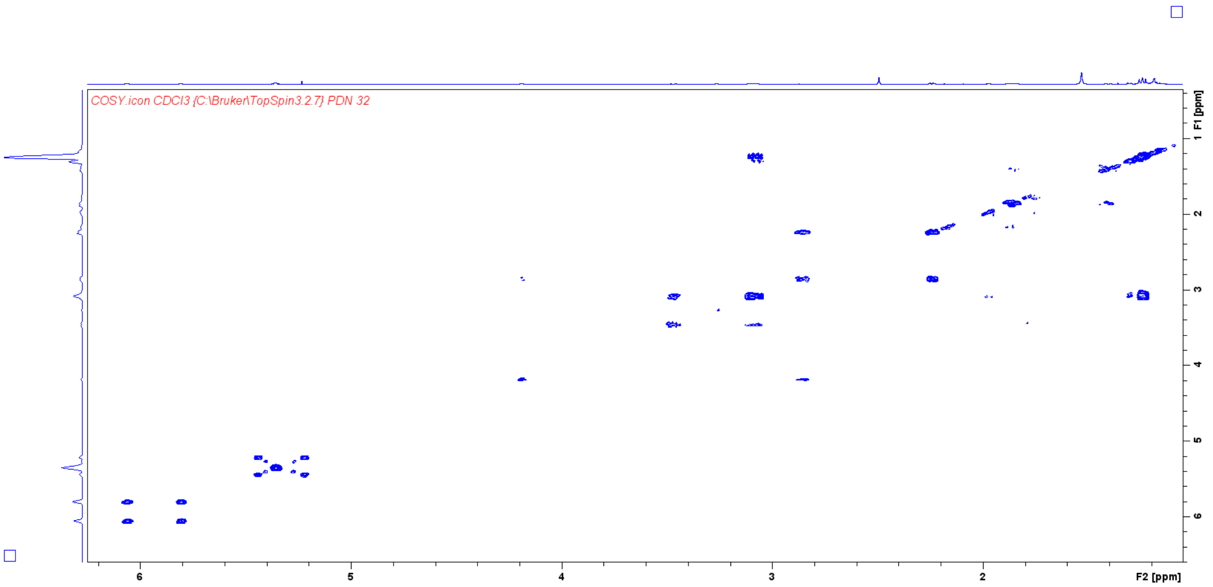

Figure S40 ^{1}H-^{1}H COSY NMR spectrum of [(η^6-cym)Ru(*R*-ahaz)Cl]Cl recorded at 300 MHz in D_6-dmso.

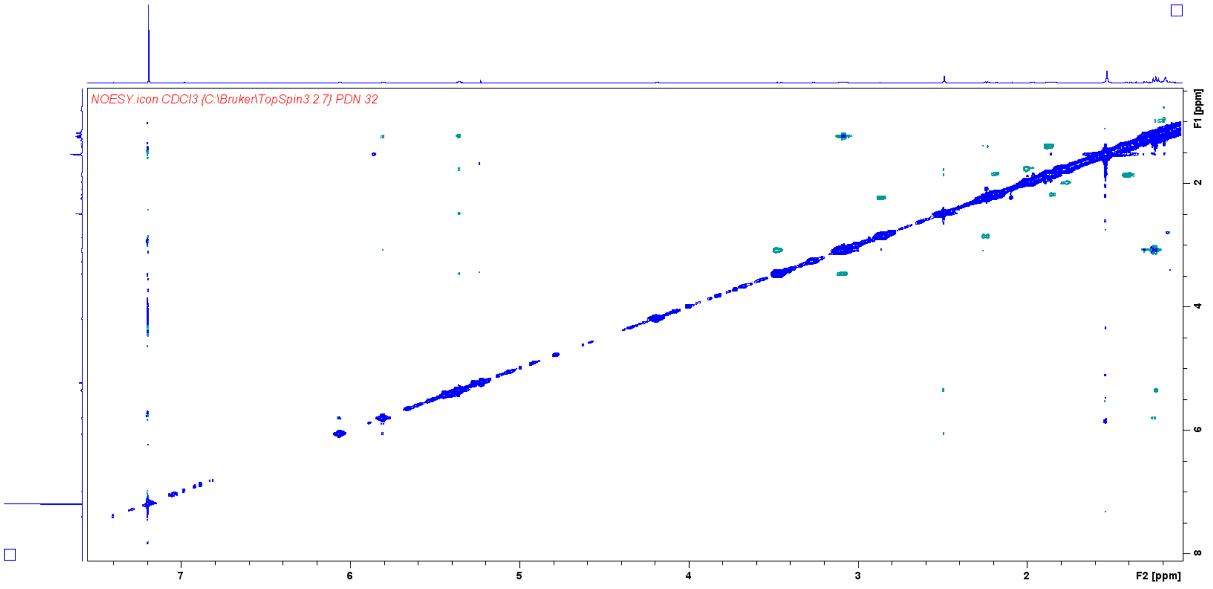

Figure S41 ^{1}H-^{1}H NOESY NMR spectrum of [(η^{6}-cym)Ru(*R*-ahaz)Cl]Cl recorded at 300 MHz in D_6-dmso.

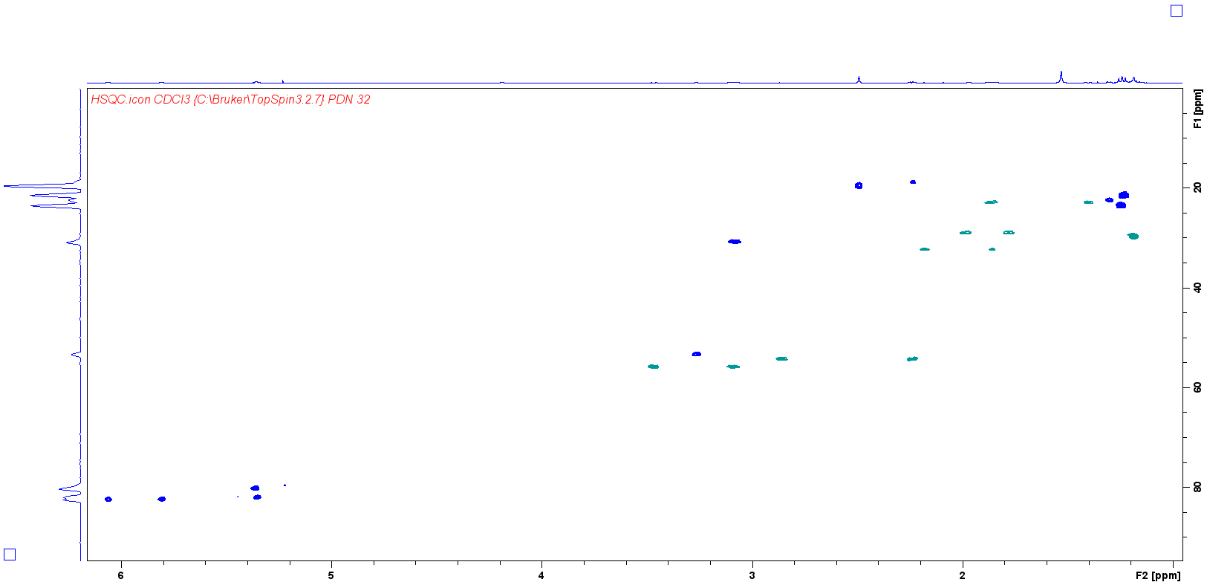

Figure S42 ^{13}C-^{1}H HSQC NMR spectrum of [(η^{6}-cym)Ru(*R*-ahaz)Cl]Cl recorded at 300 MHz in D_6-dmso.

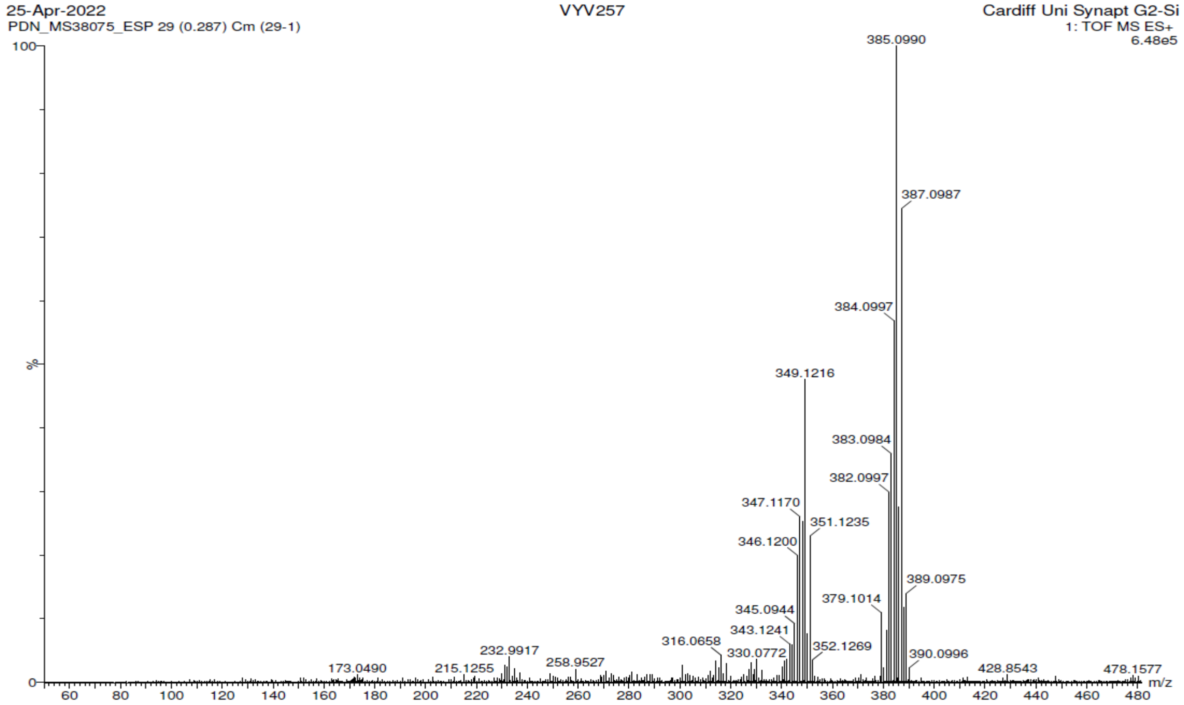

Monoisotopic Mass, Odd and Even Electron Ions
11 formula(e) evaluated with 1 results within limits (up to 50 closest results for each mass)
Elements Used:
C: 0-16   H: 0-28   N: 0-2   Cl: 0-1   102Ru: 0-1

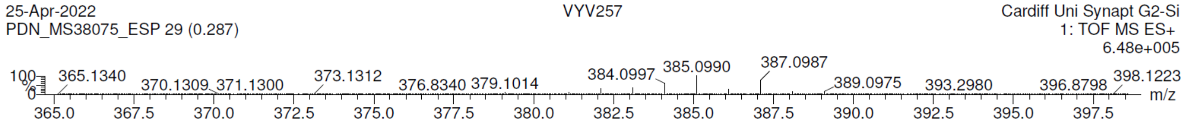

Minimum:				-1.5				
Maximum:		20.0	5.0	50.0				
Mass	Calc. Mass	mDa	PPM	DBE	i-FIT	Norm	Conf(%)	Formula
385.0990	385.0985	0.5	1.3	3.5	872.2	n/a	n/a	C16 H28 N2 Cl 102Ru

Figure S43 HRMS of [(η^6-cym)Ru(*R*-ahaz)Cl]Cl.

NMR and mass spectra of [(η^5-C_5Me_5)Rh(*R*-Bzapip)Cl]Cl.

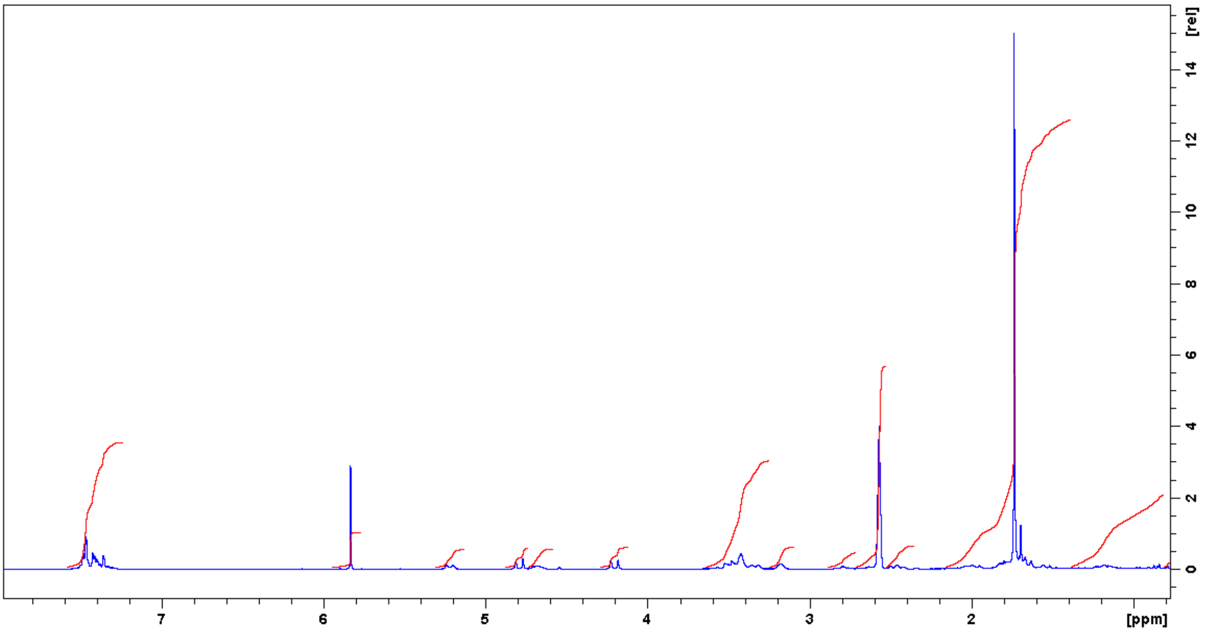

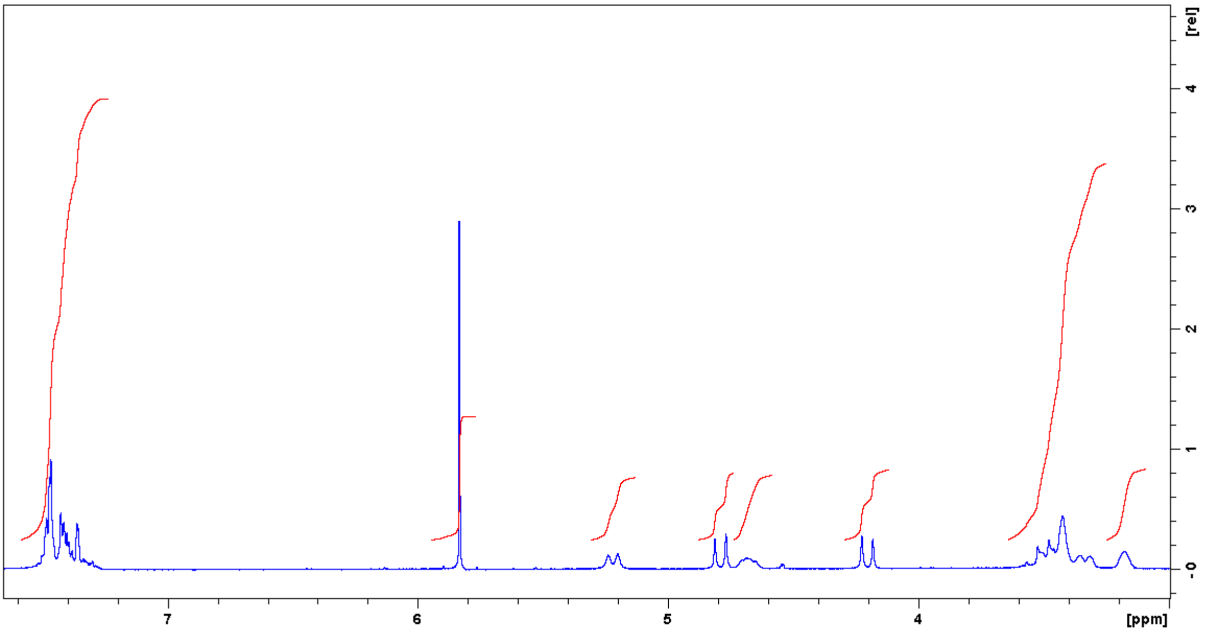

Figure S44 ^{1}H NMR spectra of [(η^5-C_5Me_5)Rh(*R*-Bzapip)Cl]Cl recorded at 300 MHz in D_6-dmso.

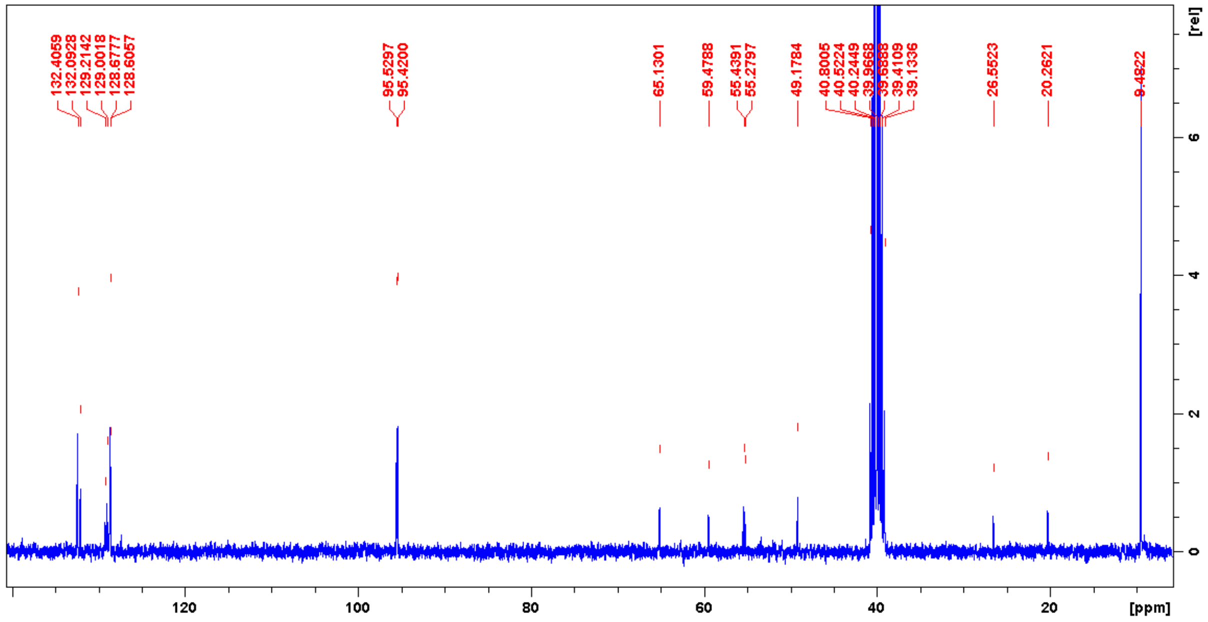

Figure S45 $^{13}C\{^1H\}$ NMR spectra of [(η^5-C_5Me_5)Rh(*R*-Bzapip)Cl]Cl recorded at 300 MHz in D_6-dmso.

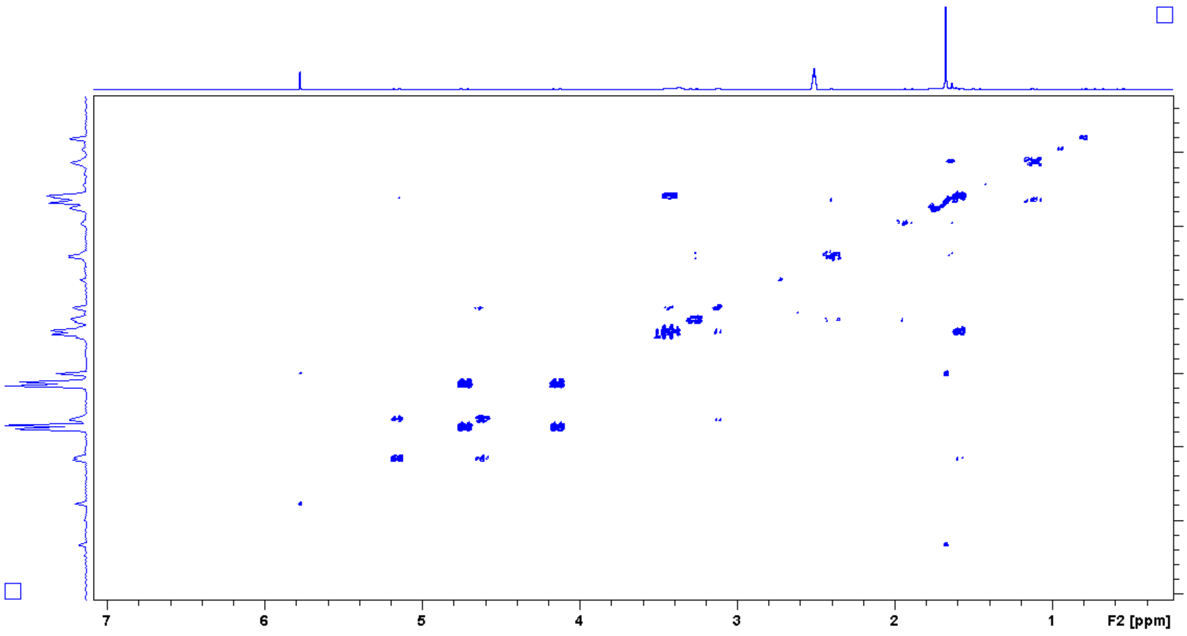

Figure S46 ^1H-^1H COSY NMR spectra of [(η^5-C_5Me_5)Rh(*R*-Bzapip)Cl]Cl recorded at 300 MHz in D_6-dmso.

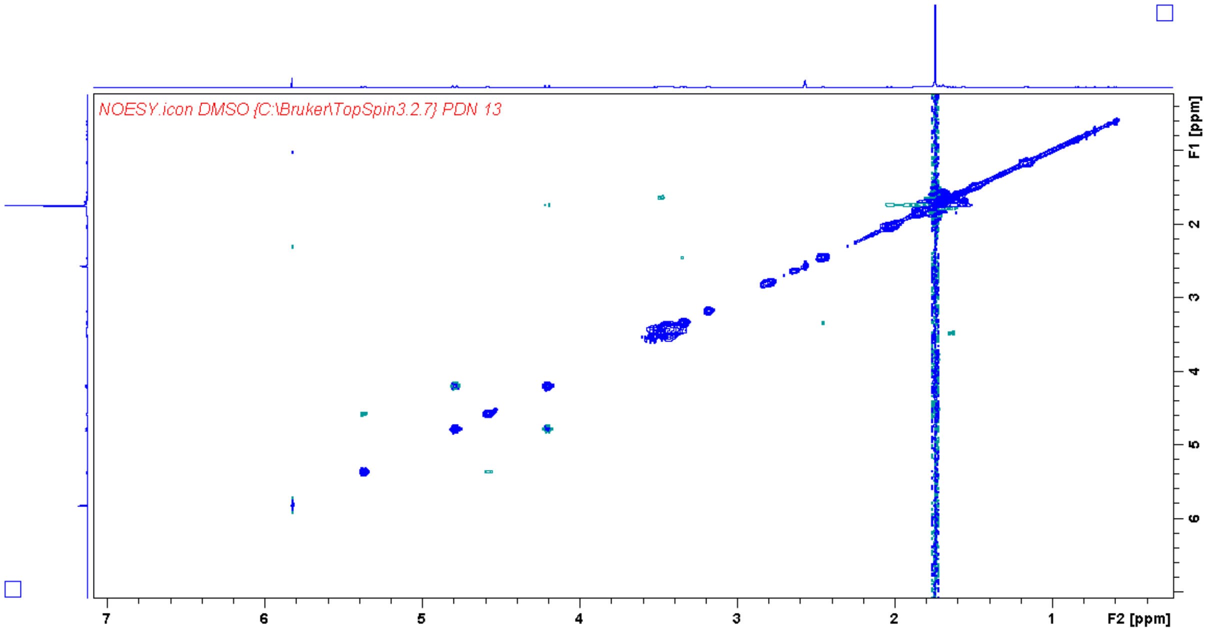

Figure S47 ^{1}H-^{1}H NOESY NMR spectra of [(η^5-C_5Me_5)Rh(*R*-Bzapip)Cl]Cl recorded at 300 MHz in D_6-dmso.

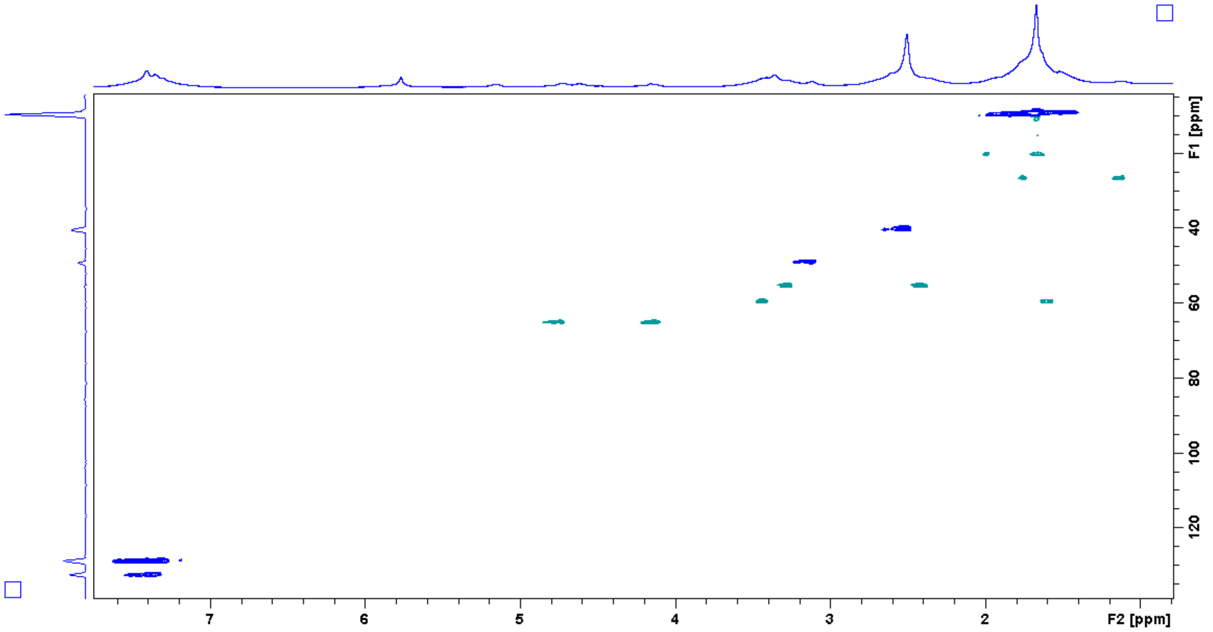

Figure S48 ^{13}C-^{1}H HSQC NMR spectra of [(η^5-C_5Me_5)Rh(*R*-Bzapip)Cl]Cl recorded at 300 MHz in D_6-dmso.

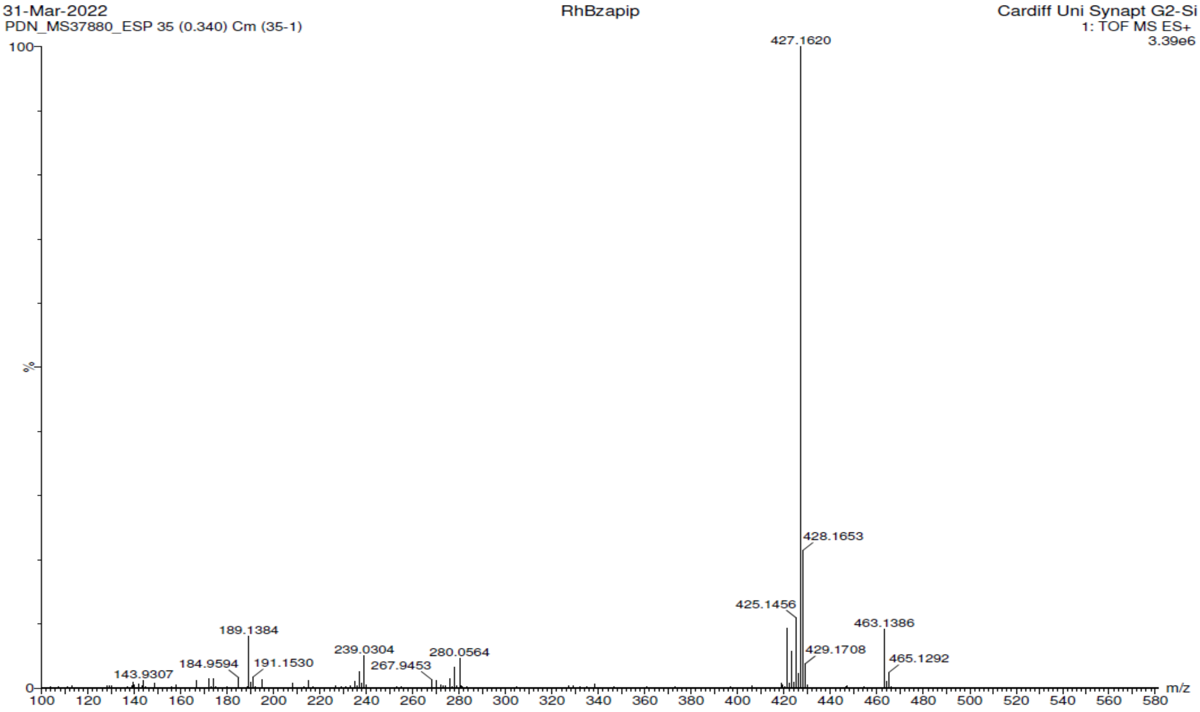

Monoisotopic Mass, Odd and Even Electron Ions
94 formula(e) evaluated with 1 results within limits (up to 50 closest results for each mass)
Elements Used:
C: 0-22 H: 0-33 N: 0-2 O: 0-5 Cl: 0-1 103Rh: 0-1

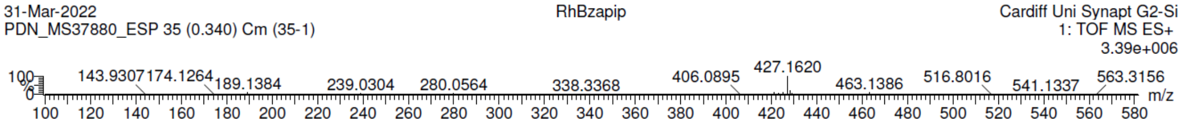

Minimum:				-1.5				
Maximum:		20.0	5.0	50.0				

Mass	Calc. Mass	mDa	PPM	DBE	i-FIT	Norm	Conf(%)	Formula
463.1386	463.1387	-0.1	-0.2	7.0	746.1	n/a	n/a	C22 H33 N2 Cl 103Rh

Figure S49 HRMS of [(η^5-C_5Me_5)Rh(*R*-Bzapip)Cl]Cl.

NMR and mass spectra of *R*-Bzapippyr.

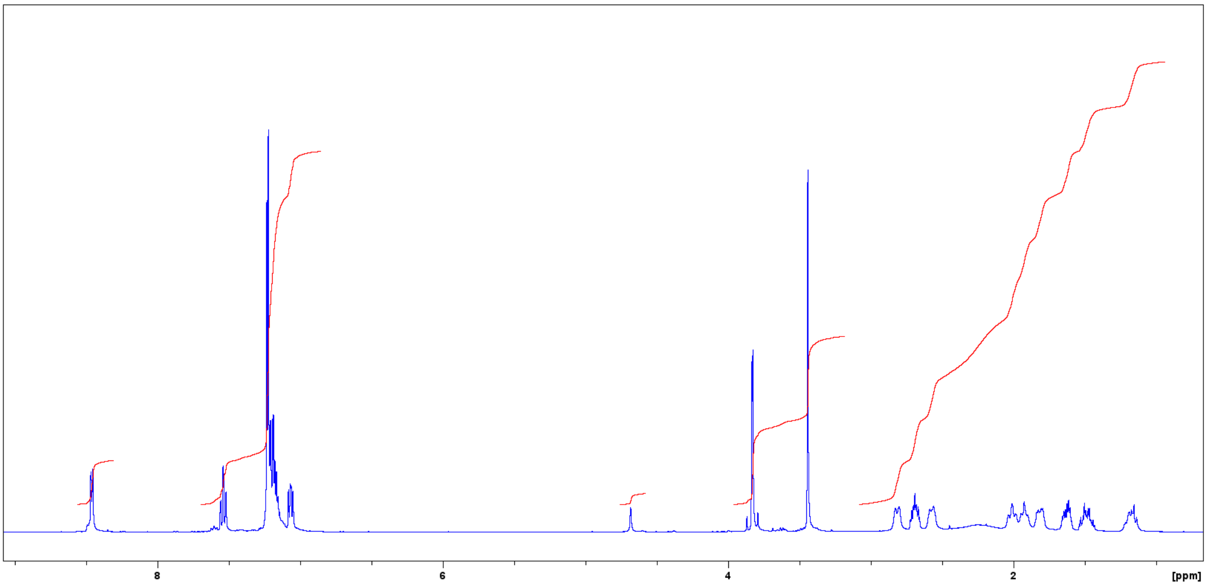

Figure S50 ^{1}H NMR spectrum of *R*-Bzapippyr recorded at 400 MHz in $CDCl_3$.

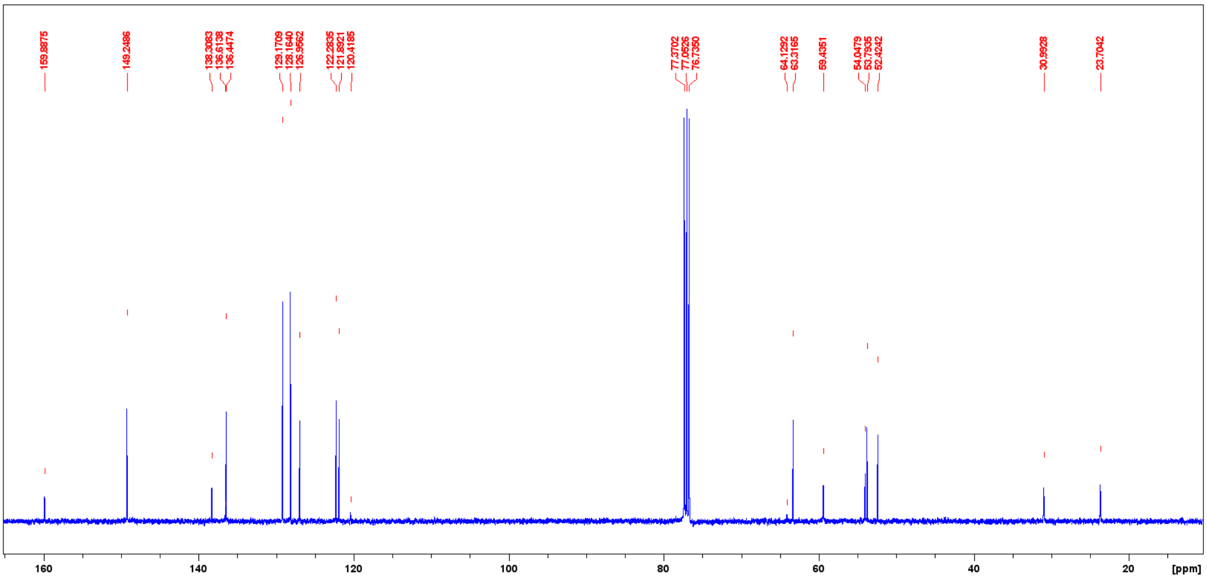

Figure S51 $^{13}C\{^{1}H\}$ NMR spectrum of *R*-Bzapippyr recorded at 400 MHz in $CDCl_3$.

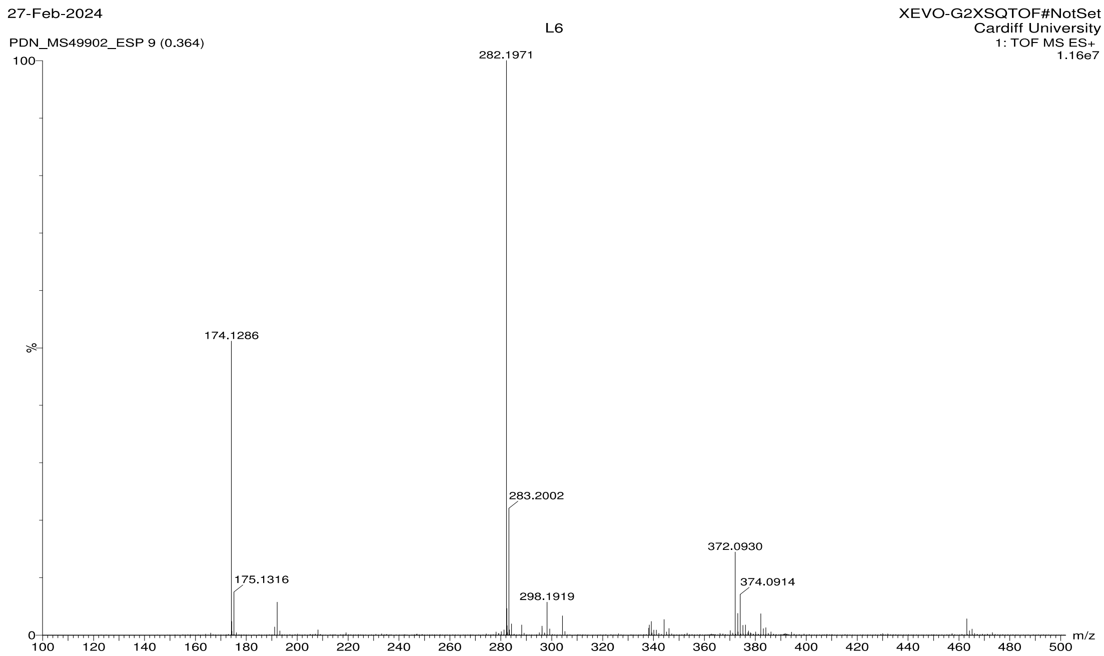

Minimum:				-1.5				
Maximum:		5.0	100.0	50.0				
Mass	Calc. Mass	mDa	PPM	DBE	i-FIT	Norm	Conf(%)	Formula
282.1971	282.1970	0.1	0.4	8.5	2313.5	n/a	n/a	C18 H24 N3

Figure S52 HRMS of *R*-Bzapippyr.

NMR and mass spectra of [(η^5-C_5Me_5)Rh(*R*-Bzapippyr)Cl]PF_6.

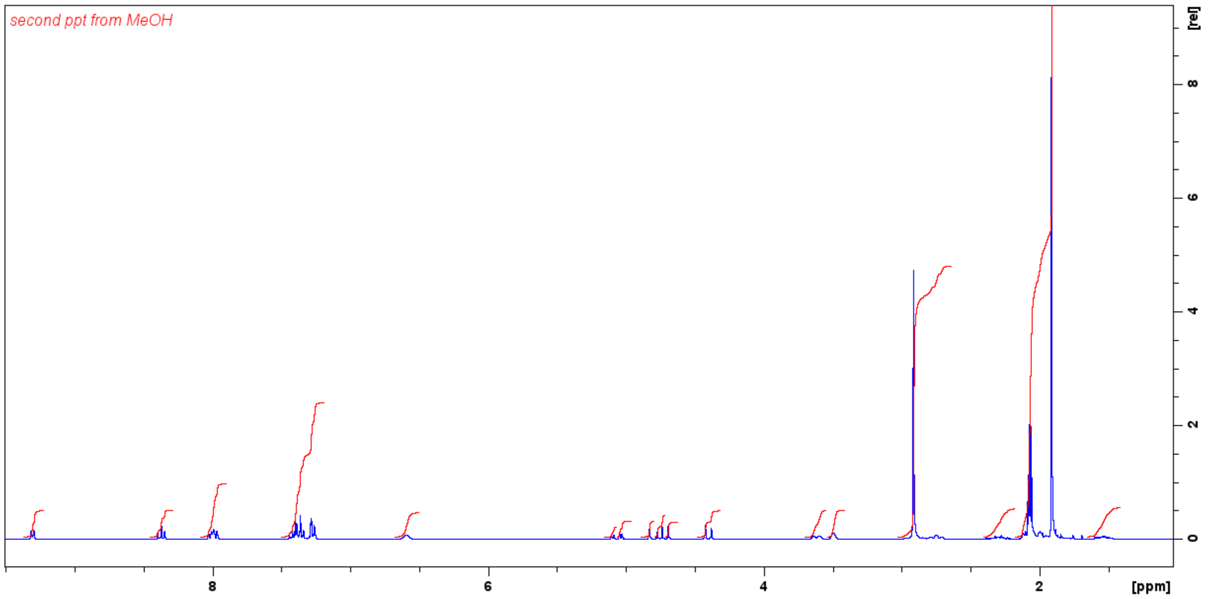

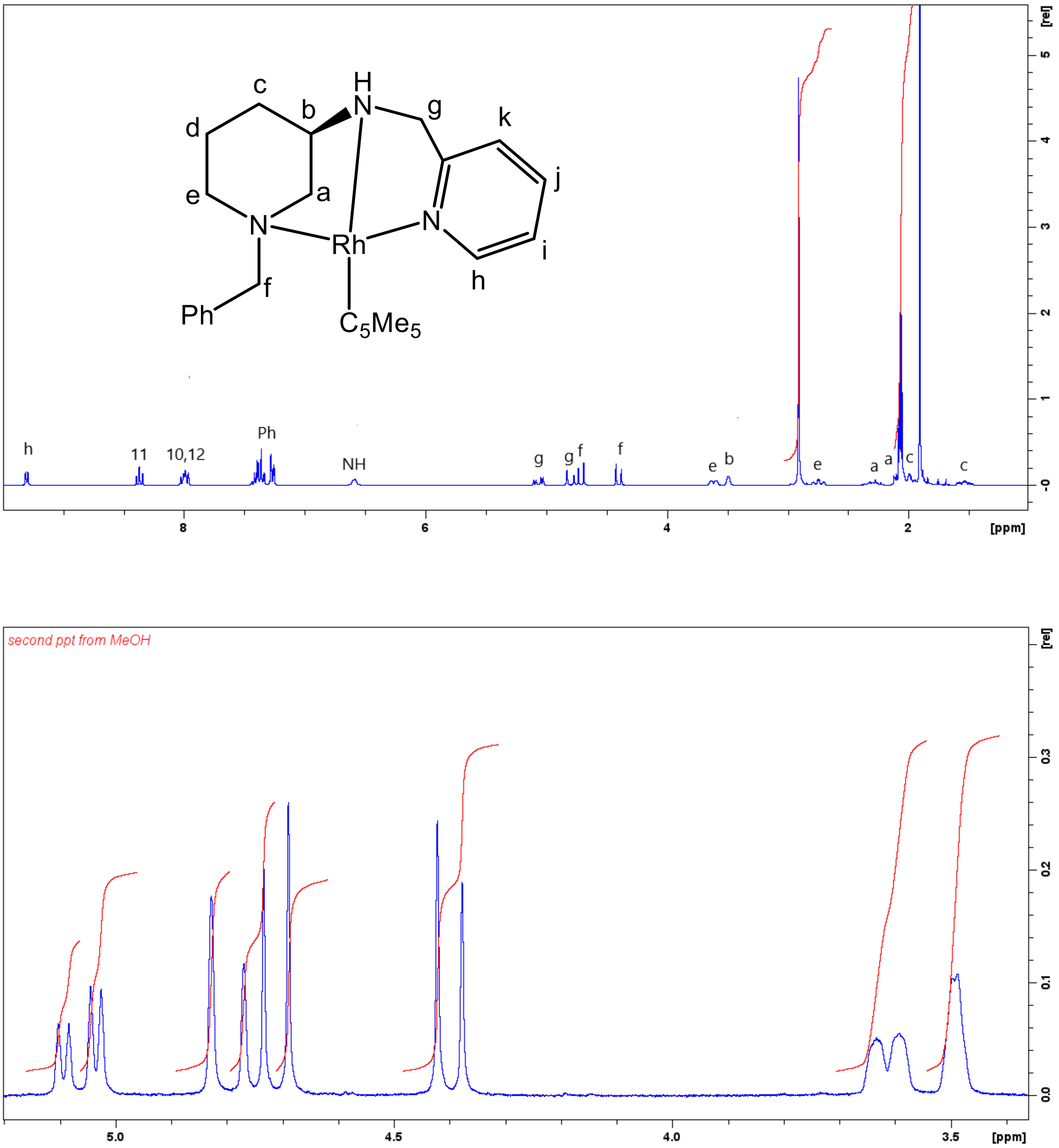

Figure S53 ^{1}H NMR spectra of [(η^5-C_5Me_5)Rh(*R*-Bzapippyr)Cl]PF_6 recorded at 300 MHz in D_6-acetone.

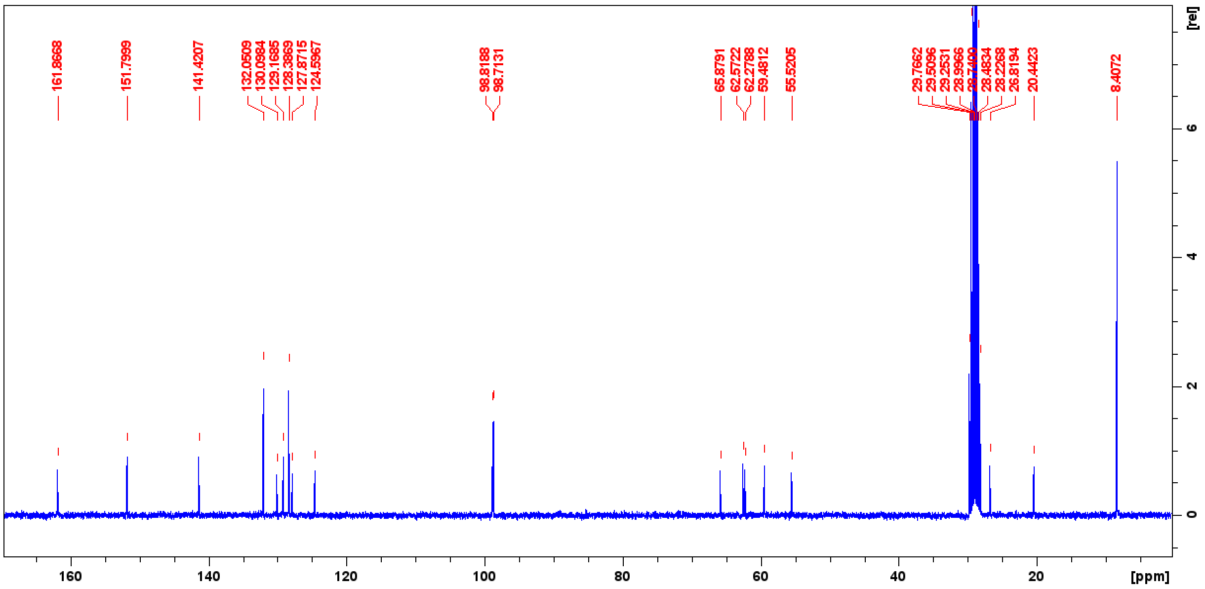
161.8668
151.7999
141.4207
132.0509
130.0984
129.1685
128.3869
127.8715
124.5967
98.8188
98.7131
65.8791
62.5722
62.2788
59.4812
55.5205
29.7662
29.5096
29.2531
28.9966
28.4834
28.2268
26.8194
20.4423
8.4072
[rel]
[ppm]

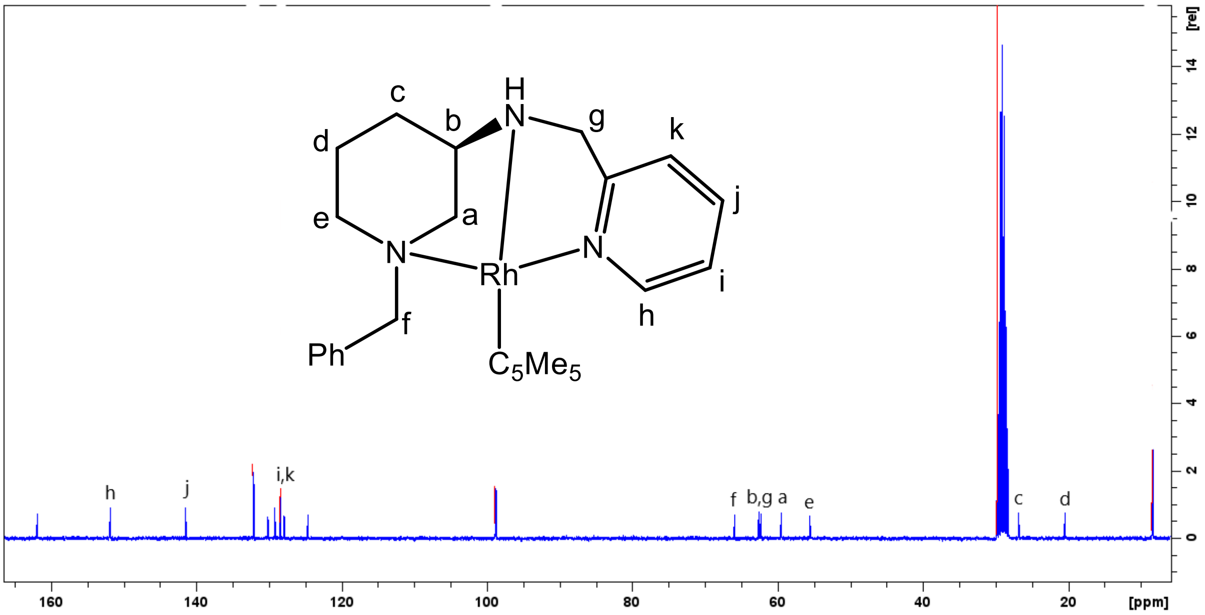
H
N
c
b
g
k
d
j
e
a
N
Rh
N
i
f
h
Ph
C_5Me_5
h
j
i,k
f
b,g
a
e
c
d
[rel]
[ppm]

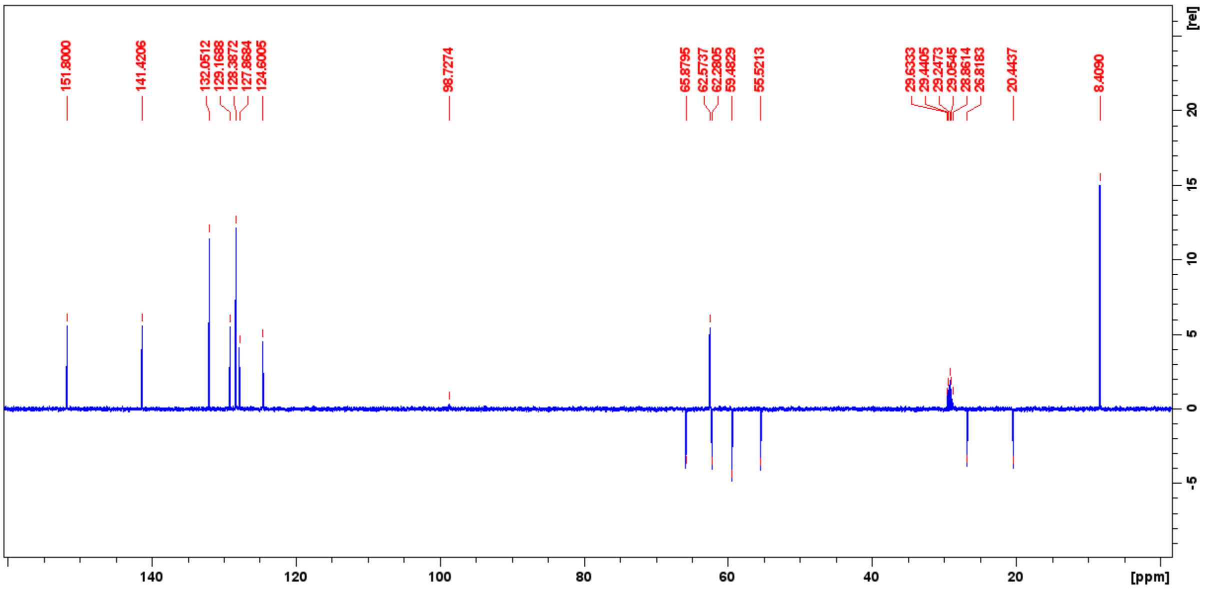
151.8000
141.4206
132.0512
129.1688
128.3872
127.8684
124.6005
98.7274
65.8795
62.5737
62.2805
59.4829
55.5213
29.6333
29.4405
29.2473
29.0545
28.8614
26.8183
20.4437
8.4090
[rel]
[ppm]

Figure S54 $^{13}C\{^{1}H\}$ NMR spectra of $[(\eta^5\text{-}C_5Me_5)Rh(\textit{R}\text{-Bzapippyr})Cl]PF_6$ recorded at 300 MHz in D_6-acetone.

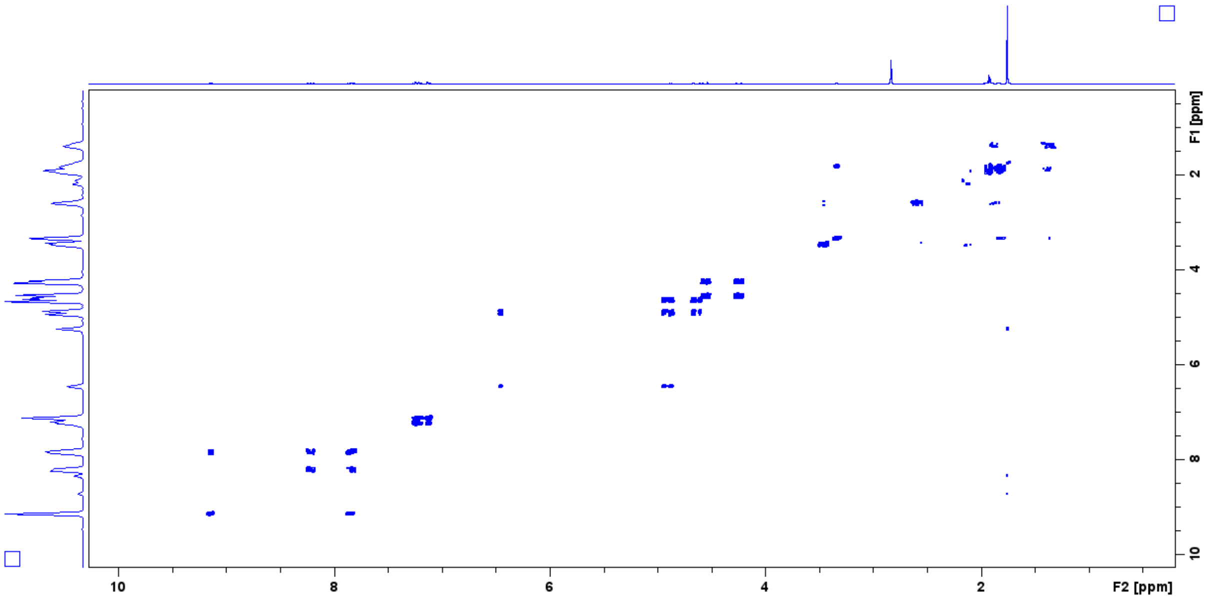

Figure S55 $^{1}H\text{-}^{1}H$ COSY NMR spectra of $[(\eta^5\text{-}C_5Me_5)Rh(\textit{R}\text{-Bzapippyr})Cl]PF_6$ recorded at 300 MHz in D_6-acetone.

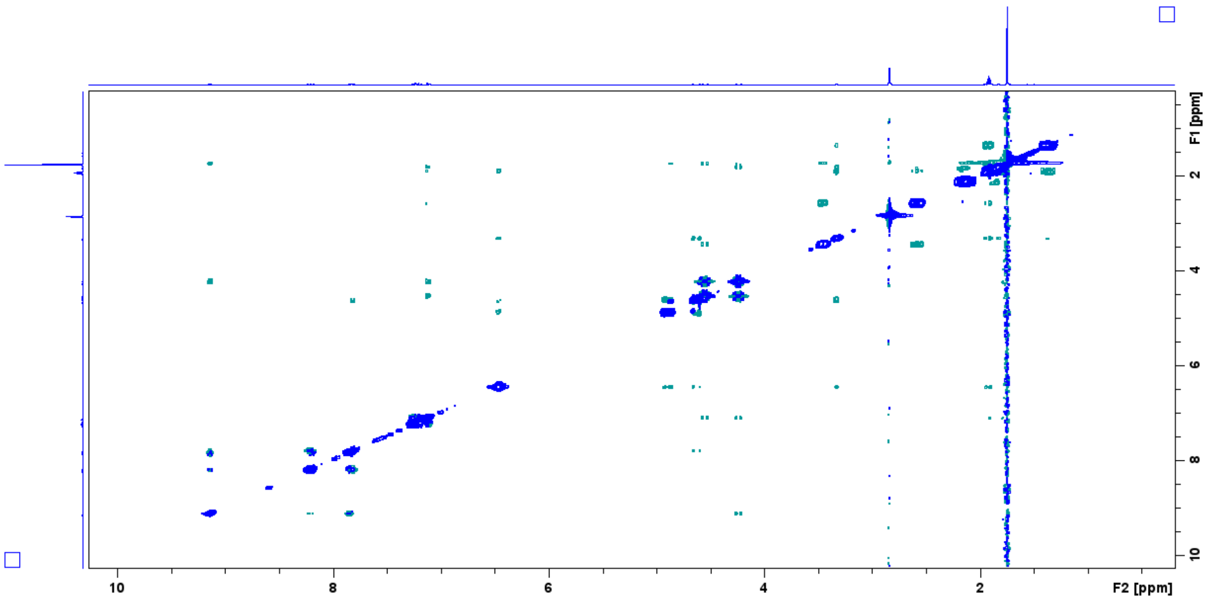

Figure S56 $^{1}H\text{-}^{1}H$ NOESY NMR spectra of $[(\eta^5\text{-}C_5Me_5)Rh(\textit{R}\text{-Bzapippyr})Cl]PF_6$ recorded at 300 MHz in D_6-acetone.

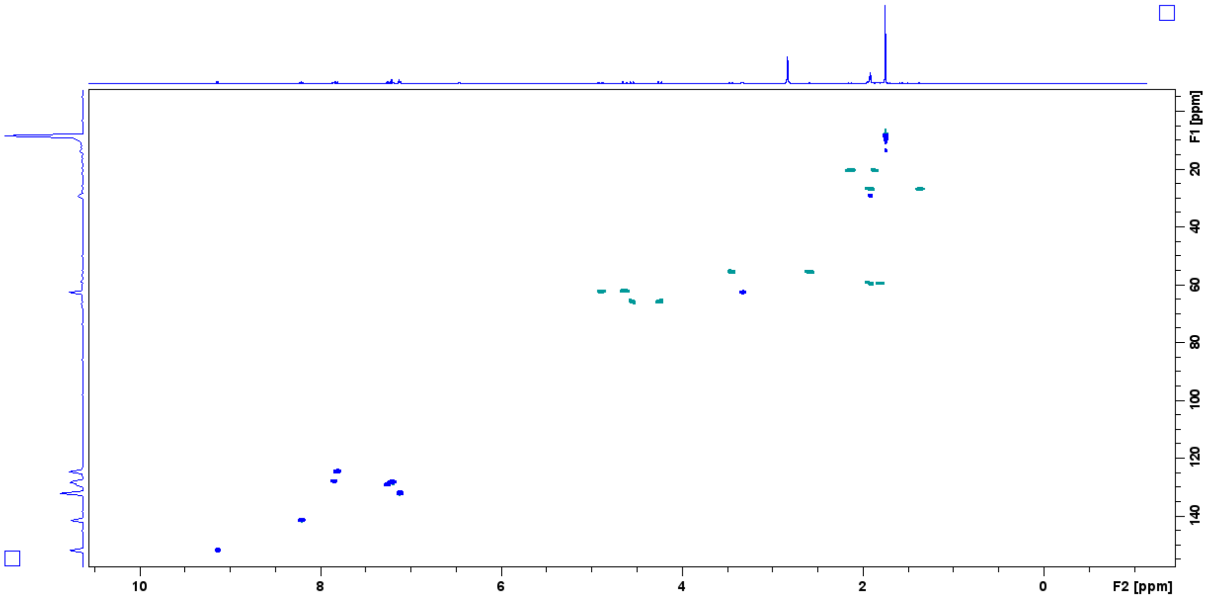

Figure S57 ^{13}C-^{1}H HSQC NMR spectra of [(η^5-C_5Me_5)Rh(*R*-Bzapippyr)Cl]PF_6 recorded at 300 MHz in D_6-acetone.

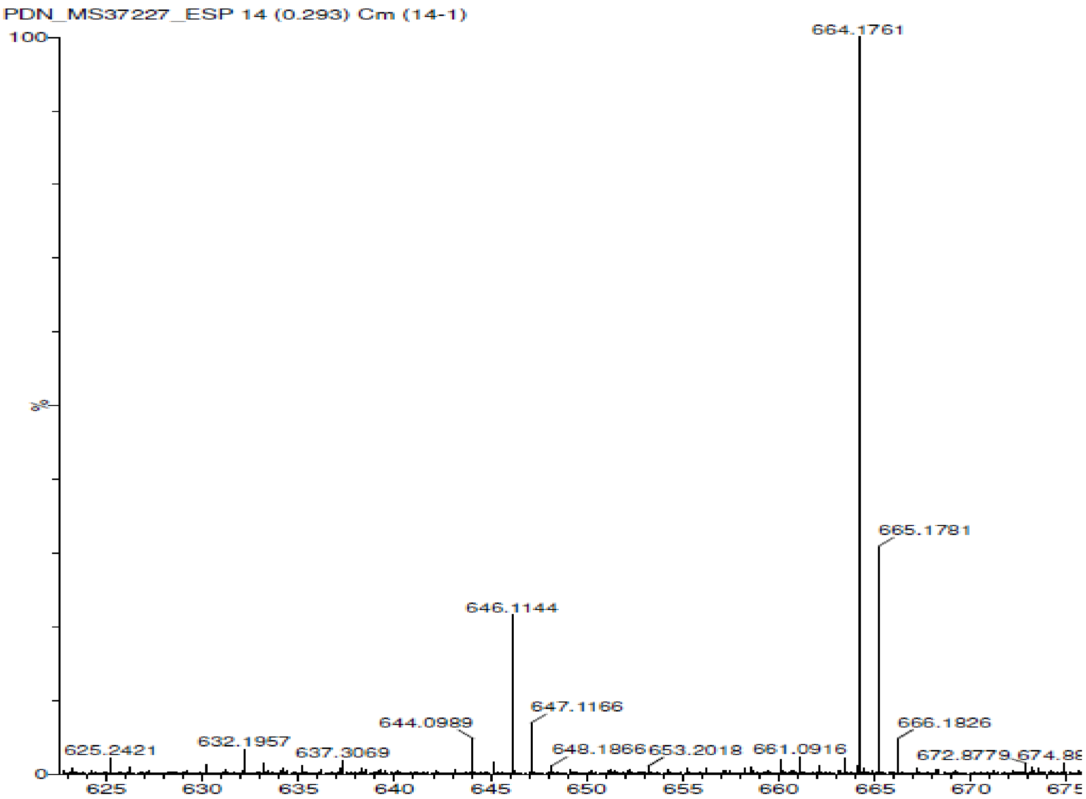

Mass	Calc. Mass	mDa	PPM	DBE	i-FIT	Norm	Conf(%)	Formula
Minimum:				-1.5				
Maximum:		5.0	5.0	100.0				
664.1761	664.1763	-0.2	-0.3	9.0	429.6	n/a	n/a	C28 H38 N3 F6 P 103Rh

Figure S58 HRMS of [(η^5-C_5Me_5)Rh(*R*-Bzapippyr)Cl]PF_6.

Crystallographic data

Compound (Identification code)	**RuL2 (pdn2201)**	**RuL3 (pdn2205c)**
CCDC reference		
Empirical formula	$C_{15}H_{26}ClF_6N_2PRu$	$C_{16}H_{28}ClN_2RuPF_6$
Formula weight	515.87	529.89
Temperature /K	296(2)	298(2)
Wavelength /Å	0.71073	1.54184
Crystal system	Orthorhombic	Orthorhombic
Space group	$P2_12_12_1$	$P2_12_12_1$
a/Å	10.3999(4)	8.5419(2)
b/Å	11.5477(5)	11.5358(3)
c/Å	16.4273(8)	21.8557(7)
α/°	90	90
β/°	90	90
γ/°	90	90
Volume/$Å^3$	1972.84(15)	2153.61(10)
Z	4	4
Density (calculated)/ Mgm^{-3}	1.737	1.634
Absorption coefficient/ mm^{-1}	1.067	8.249
S1Crystal size/ mm^3	0.540x0.150x0.100	0.291x0.11x0.056
Reflections collected	4991	4164
Independent reflections	4333	3879
R(int)	0.0333	0.0351
Data / restraints / parameters	4991 / 357 / 302	4164 / 463 / 312
Goodness-of-fit on F^2	1.042	1.078
R1, wR2 [$I>2\sigma(I)$]	0.0327, 0.0611	0.0399, 0.1057
R1, wR2 (all data)	0.0438, 0.0665	0.0438, 0.1103
Largest diff. peak and hole e.$Å^{-3}$	0.504 and -0.367	1.440 and -0.511
Flack parameter	-0.044(16)	-0.028(9)

Compound (Identification code)	**RhL2 (pdn2204c)**	**RhL2py (pdn2206)**
CCDC reference		
Empirical formula	$C_{15}H_{27}ClF_6N_2PRh$	$C_{28}H_{38}F_{12}N_3P_2Rh$
Formula weight	518.71	809.46
Temperature /K	180.00(10)	293(2)
Wavelength /Å	1.54184	1.54184
Crystal system	Orthorhombic	Orthorhombic
Space group	$P2_12_12_1$	$P2_12_12_1$
a/Å	8.5623(2)	11.4970(3)
b/Å	10.1669(2)	13.7928(3)
c/Å	22.7249(5)	21.0676(6)
α/°	90	90
β/°	90	90
γ/°	90	90
Volume/Å^3	1978.25(7)	3340.81(15)
Z	4	4
Density (calculated)/ Mgm^{-3}	1.742	1.609
Absorption coefficient/ mm^{-1}	9.528	5.878
S1Crystal size/ mm^3	0.436x0.273x0.056	0.170x0.090x0.070
Reflections collected	3841	6497
Independent reflections	3696	5892
R(int)	0.0365	0.0290
Data / restraints / parameters	3841 / 457 / 305	6497 / 750 / 549
Goodness-of-fit on F^2	1.053	1.024
R1, wR2 [I>2σ(I)]	0.0345, 0.0897	0.0312, 0.0784
R1, wR2 (all data)	0.0363, 0.0917	0.0356, 0.0820
Largest diff. peak and hole e.Å^{-3}	0.849 and -0.631	0.404 and -0.401
Flack parameter	-0.024(10)	-0.008(5)

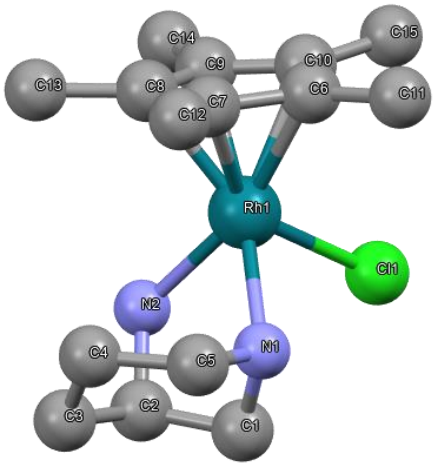
C14
C15
C9
C10
C13
C8
C6
C11
C12
C7
Rh1
Cl1
N2
N1
C4
C5
C3
C2
C1

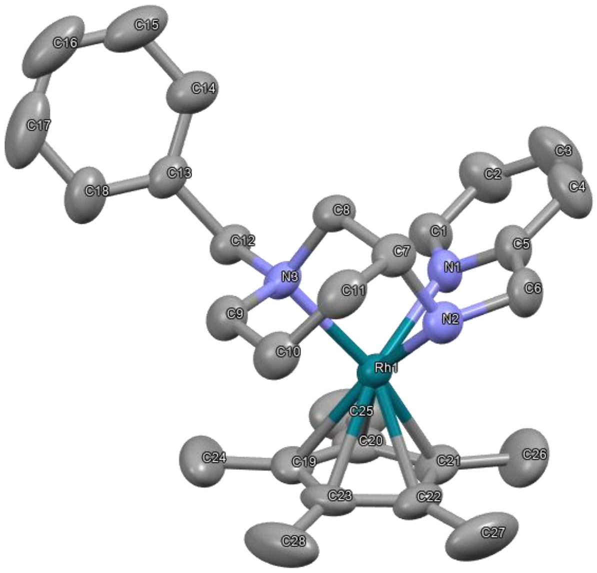
C15
C16
C14
C17
C3
C2
C13
C4
C18
C8
C1
C12
C7
C5
N1
N3
C11
C6
C9
N2
C10
Rh1
C25
C20
C24
C19
C21
C26
C23
C22
C28
C27

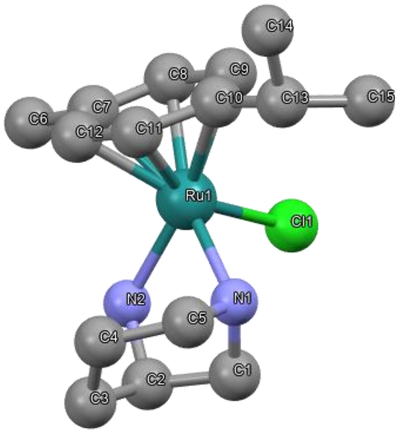
C14
C8
C9
C7
C10
C13
C15
C6
C12
C11
Ru1
Cl1
N2
N1
C5
C4
C2
C1
C3

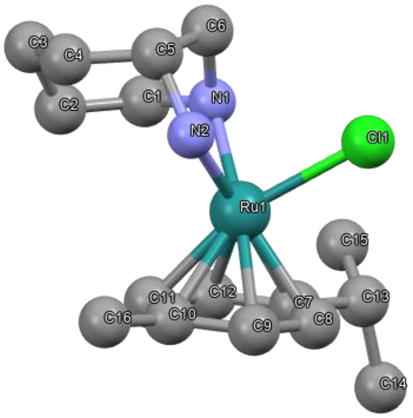
C6
C3
C4
C5
C1
N1
C2
N2
Cl1
Ru1
C15
C12
C11
C13
C7
C16
C10
C9
C8
C14

ORCA input commands

The following commands were used to run step 1 of the calculations:

! r2scan-3c cpcm([solvent]) verytightopt verytightscf freq kdiis defgrid3

%geom enforcestrictconvergence true end

%freq temp [temp] end

%scf convcheckmode 0 end

The following commands were used to run step 2 of the calculations:

! wb97m-v def2-qzvppd cpcm([solvent]) verytightscf kdiis defgrid3

%scf convcheckmode 0 end

%method functional hyb_mgga_xc_wb97m_v end

Where [solvent] / [temp] are "ethanol" / 353.15 for the main catalytic cycles and "ch2cl2" / 273.15 for the epimer selectivity calculations.

Only for the transition state calculations, the following modified "%geom" block was used, where [atoms] is a list of select atoms that were deemed to be "active" for the transition states.

%geom

calc_hess true

ts_active_atoms { [atoms] } end

ts_active_atoms_factor 1.5

enforcestrictconvergence true

end

For the nudged elastic band calculations, the following general input script was used.

! zoom-neb-ci r2scan-3c cpcm(ethanol) kdiis

%maxcore 4000

%pal nprocs 40 end

%neb

product [product geometry xyz file enclosed in ""]

preopt true

free_end true

nimages 20

reparam 10

opt_method fire

printlevel 4

end

The rest of the parameters for the calculations should be takes as those defined by default in ORCA 5.0.4.

Supporting software used for analysis of the DFT results

Avogadro molecular editor (v1.2.0) was used to prepare input structures and visualise calculated geometries. The free version of Mercury by CCDC (v. 2023.3.0, Build 392256) along with POV-Ray for Windows v3.7 was used to create POV-Ray images of select optimised structures. The mechaSVG application was used to prepare the reaction energy profiles.

Cartesian coordinates for the optimised geometries for the Rh(L2) cycle

Charge: 1

Multiplicity: 1

Number of atoms: 46

Rh L2 / pre-catalyst (cycle starting point)

C	-2.40965286946630	1.89867974147964	-0.52766679805816
C	-3.17215199464251	0.73001940962776	0.10628624362632
C	-2.62836895642776	-0.62184899723611	-0.33652423432351
H	-4.23501424397667	0.79091538226025	-0.15206473111419
H	-3.10380621893873	0.79995195252809	1.19451389659639
N	-1.15188045379474	-0.65980384845164	-0.14462000339525
H	-3.07905566210919	-1.43768026156927	0.23249978749589
H	-2.84509414552635	-0.79402744747650	-1.40180517039882
C	-0.52890873320467	0.34805562985579	-1.04911079798897
C	-0.89284296341150	1.70878302153869	-0.48323912028791
H	-0.92217664908301	0.23928234511450	-2.06919229090335
H	0.55122866321111	0.19598189287267	-1.06386336487612
N	-0.36526040392879	1.73927906433616	0.91860375374678
H	-0.40859741024246	2.50020536630680	-1.06997565499256
H	-2.68940335699791	2.84267649729672	-0.04404744359361
H	-2.68250386516622	1.98880459617648	-1.58651965796720
Rh	-0.45316804607851	-0.20290169620651	1.88502894485554
H	-0.83565750896203	2.46300274966299	1.45812239557301
H	-0.80415079572911	-1.58560290763415	-0.38512801632941
H	0.62265313099678	1.98564078768900	0.89852672746292
Cl	1.80235694465942	-0.62801618880504	1.01882205217956
C	-1.97929043303848	-1.15788828800741	3.17217691929058
C	-1.79998418411590	0.20854488772582	3.56736159608731
C	-0.41486128513086	0.37510508364858	3.97114102962106
C	-2.85368951862163	1.24660696354121	3.74222120978117

C	0.23168461774844	-0.89292404236797	3.85081542070960
C	-0.71159308650919	-1.83640027160525	3.29092030602830
C	-0.46651582366948	-3.28781578681148	3.06124482805370
C	-3.26878359601589	-1.81398732752335	2.83230462924638
C	0.17080079090183	1.62721038034044	4.52484220417549
C	1.61860311326615	-1.20590008547624	4.27644774962455
H	-0.27742377278458	2.51079652164297	4.06202883630242
H	1.25203132864914	1.66414332648488	4.37370118333858
H	-0.02299294689412	1.68161975671615	5.60397152685945
H	-3.80805702814349	0.94593719481818	3.30659686045699
H	-2.55474043970152	2.20746827380260	3.31153011029962
H	-3.01058185700836	1.40861732302973	4.81650895422817
H	-3.69843789493412	-2.21720946036463	3.75935074528842
H	-3.12961260868447	-2.65265742069536	2.14656429582309
H	-3.99546586479179	-1.11770302669290	2.40868865898479
H	-1.11214151945415	-3.67807829815027	2.27062895964724
H	-0.67390159851556	-3.85569698911977	3.97764155535922
H	0.57283975768993	-3.47232862251848	2.77928727336783
H	2.05319993084217	-2.01134221726449	3.68279628199420
H	1.58636480190499	-1.53093209391339	5.32531521223153
H	2.26976465583039	-0.33199287060590	4.21295713589256

Charge: 0

Multiplicity: 1

Number of atoms: 45

Rh L2 / pre-catalyst NH_2-deprotonated

C	-2.38877602236452	1.94935869735773	-0.49023663981495
C	-3.19064826833718	0.77947376836623	0.09015268567557
C	-2.66073798757896	-0.57028313684474	-0.37777131644916
H	-4.24947246847425	0.86315810146211	-0.18278642586819
H	-3.13307126469778	0.81413304328144	1.18019991789174
N	-1.19078202758715	-0.63761419980582	-0.16485850836896
H	-3.13452457215167	-1.39194570742816	0.16591261399147
H	-2.86663550939592	-0.71325675417882	-1.45058233913324
C	-0.53447548674552	0.38881058789919	-1.02503832466901
C	-0.86794671473366	1.72139783353852	-0.38049959858920
H	-0.91728301774234	0.32256696896067	-2.05433024311574
H	0.54145992117462	0.20808978086605	-1.02140397596905
N	-0.32483989029337	1.67153387771860	0.98821803342252
H	-0.36628438819012	2.52296077401787	-0.95317577543089
H	-2.66478797375676	2.88133689343066	0.02067792776641
H	-2.63877599534180	2.08095198286800	-1.55326961292187
Rh	-0.48841143821626	-0.20521466472966	1.85806699749823
H	-0.87536776359027	2.31632712535542	1.55549447441477
H	-0.85314053136715	-1.56249730893934	-0.42133225291206
Cl	1.80429443392820	-0.95602727278201	0.89549737601162
C	-1.95457889467998	-1.19315432934882	3.16125415736637
C	-1.79642904395754	0.20240448401951	3.52394896452282
C	-0.42447543199925	0.38698511974698	3.93359829233892
C	-2.87647089140069	1.21634470338927	3.70303039793328
C	0.23384518804631	-0.88443793928587	3.83093724873188
C	-0.70640125320831	-1.86576618979563	3.35521175252582
C	-0.44785214578287	-3.32551663004781	3.18625942117676

C	-3.24067054264626	-1.85403619155633	2.80616809238025
C	0.15784358752837	1.65026664071919	4.46940086441807
C	1.63429542442616	-1.15813915648432	4.24862771295261
H	-0.30232851046406	2.52603739700132	4.00257373969699
H	1.23612984934461	1.69743244110643	4.29685068643717
H	-0.01350835743725	1.72090440473601	5.55164446960587
H	-3.81805164309289	0.90254857250097	3.24798242559096
H	-2.59466455505713	2.18847608388278	3.28582369630625
H	-3.05799429544644	1.36174529614646	4.77602381877389
H	-3.70128884130256	-2.24506104002386	3.72339812468812
H	-3.08890925224945	-2.69809791030288	2.12870703617599
H	-3.95085763439713	-1.15915880964803	2.35247452594599
H	-1.10707802846126	-3.76066266937736	2.43023262399689
H	-0.61977058867214	-3.86323745605069	4.12829154114691
H	0.58552578024401	-3.51165140897046	2.88147620941452
H	2.05438090086499	-2.01527960163375	3.72067861472668
H	1.64084257920539	-1.37806996856065	5.32510001987268
H	2.28180356605731	-0.29518623257642	4.07846054984417

Charge: 0

Multiplicity: 1

Number of atoms: 45

Rh L2 / pre-catalyst ring N-deprotonated

C	-2.48774125955697	1.79289893735541	-0.62478578419853
C	-3.18551959017493	0.61198614146145	0.05941997238245
C	-2.48959733966483	-0.71927621685459	-0.26503583819760

H	-4.24178556006750	0.57510164126424	-0.23961722213435
H	-3.16366672295744	0.76316839508405	1.14295443176561
N	-1.03615907500194	-0.69328986343037	-0.06709175780056
H	-2.92990886032744	-1.52608846685408	0.32954320375662
H	-2.68846114356694	-0.96727953652185	-1.32548320292929
C	-0.50145652197414	0.32471898499143	-0.97962104523867
C	-0.96545891817774	1.70558852643215	-0.51850531284679
H	-0.86236521411751	0.14609056193563	-2.00746078949673
H	0.59159682404006	0.28218589719934	-0.99331332317206
N	-0.50701876731288	1.81801873306118	0.90132307941236
H	-0.50578118636707	2.50796625017595	-1.11458510959176
H	-2.84916780636449	2.74656801930135	-0.21584486720264
H	-2.72802379555644	1.79594649724708	-1.69636056658561
Rh	-0.46449984275123	-0.13256994449793	1.86660933227587
H	-1.06121264006423	2.50388727566465	1.40864503300038
H	0.45825079459161	2.14091565942533	0.92191620820027
Cl	1.97587509504797	-0.22493940717887	1.11828375136222
C	-1.98424355530659	-1.08782252885327	3.13964866521642
C	-1.74587921734347	0.26086295089161	3.57888201524642
C	-0.37072158102330	0.35935290761205	4.03237469124727
C	-2.76844910637831	1.32888535278691	3.76851095262838
C	0.23094952645307	-0.91126434782745	3.84466834655530
C	-0.73733206643469	-1.80111869365423	3.22809952697167
C	-0.53055630007471	-3.25106453445697	2.94361261972674
C	-3.31545967193393	-1.69440806749821	2.85807492384698
C	0.24815080604867	1.57084195141429	4.64589745843421
C	1.60616953639068	-1.30386347663140	4.25140039378830

H	-0.14632751442081	2.48623179705136	4.19469295700820
H	1.33403593756046	1.57041525188023	4.52118836426228
H	0.03308283984212	1.61368042567524	5.72184511629975
H	-3.69639474458000	1.11190068982597	3.23616391303634
H	-2.39947158146444	2.30791956354500	3.44704265730523
H	-3.00611770086730	1.40940097858444	4.83762880203586
H	-3.77171750435887	-1.98982349525579	3.81285635254686
H	-3.23000147077133	-2.59315348658762	2.24356865878322
H	-4.00027122910575	-0.99735942159822	2.36960305721145
H	-1.18505309684635	-3.59161008371344	2.13667397825505
H	-0.74851442707931	-3.85750270727061	3.83313178024690
H	0.50294245448452	-3.45081021464592	2.64833541868399
H	2.03896009003288	-2.03039526481976	3.56094500989516
H	1.55929626675511	-1.76780328702033	5.24625814935700
H	2.27499484074580	-0.44290034469540	4.30866599864969

Charge: 0

Multiplicity: 1

Number of atoms: 43

Rh L2 / bis-amide (active catalyst)

C	-2.49382788836691	1.45700905234260	-0.96158155863440
C	-3.07605347144515	0.16409088459564	-0.37562958475653
C	-2.04661579601953	-0.98143954663489	-0.39512051570360
H	-3.98118875772610	-0.12847260419091	-0.92628839631819
H	-3.37474069777332	0.34236512677635	0.66505134820904
N	-0.71119918525227	-0.62562862412328	0.11399463592830

H	-2.43866088043421	-1.83193274105041	0.17932885709108
H	-1.93476946403648	-1.32988827877413	-1.44130729103177
C	-0.21142793301271	0.48277416603451	-0.72243637976137
C	-1.06454769210822	1.70777738335607	-0.43735680670280
H	-0.25014661997162	0.21121792553426	-1.79317351347462
H	0.83652803259573	0.69274215319667	-0.46229225009803
N	-1.02547181792771	1.83205621515207	1.02592797819838
H	-0.65571012453342	2.61475518758586	-0.91691791104642
H	-3.14296248097037	2.31190799621555	-0.72733383346210
H	-2.44188382384029	1.38459005578234	-2.05772150263558
Rh	-0.88121258543105	0.18148088469098	2.03393737490363
C	-2.15416696707248	-0.91498632360628	3.36948182272377
C	-1.80686018599280	0.34607985534586	4.02499860879497
C	-0.40325495010385	0.36596388879666	4.19028708970759
C	-2.78790983492633	1.38239007881782	4.45974252056551
C	0.13619460552385	-0.88707105423603	3.65778991373173
C	-0.95037071704306	-1.68987902672619	3.21359650547026
C	-0.86430793997040	-3.06935923983904	2.65322190187953
C	-3.54232728227572	-1.38577418995466	3.10559744108056
C	0.41943988459322	1.44144603842880	4.81468039725052
C	1.57506855486330	-1.27455728944149	3.69405980536203
H	-0.11822241240653	2.39206848156140	4.84518919033457
H	1.35370856732241	1.59388331833426	4.26587507818695
H	0.68463254638370	1.16899963436704	5.84487092129396
H	-3.59980127860308	1.48712336105226	3.73399106258919
H	-2.31143368610960	2.35831269671798	4.58085821625988
H	-3.23839856684378	1.10526855302271	5.42198469562597

H	-3.94274152064156	-1.88012609152123	4.00166812193826
H	-3.57391226156130	-2.10741128716214	2.28550720944356
H	-4.20762140273472	-0.55375804056108	2.86062221575720
H	-1.65547263616650	-3.25114383937920	1.92106128817876
H	-0.96929933061069	-3.81578682877822	3.45102639894593
H	0.09646122039561	-3.23913850116485	2.16003362629191
H	1.80204055653286	-2.04047935309851	2.94848132144344
H	1.83387984951472	-1.67945511635733	4.68219863562328
H	2.22382538115293	-0.41329547102846	3.51155247219449
H	-1.47438900696643	2.67840050992065	1.36421288862096

Charge: 0

Multiplicity: 1

Number of atoms: 55

Rh L2 / IPA TS

C	-1.55523918957370	1.32037323090244	-3.06586192088034
C	-2.18370049981740	0.06256716001897	-2.46050132749469
C	-1.19224890018741	-1.11366463367713	-2.45662154082652
H	-3.09355937282045	-0.21091800664645	-3.01215102205072
H	-2.48359742078624	0.27728000647031	-1.43010818687522
N	0.13828123452842	-0.80114225511894	-1.91498964883484
H	-1.62177820433924	-1.95846418064696	-1.90570374564082
H	-1.06284249941083	-1.45691773695515	-3.50229879346462
C	0.69039901636854	0.27912236288544	-2.73678311844248
C	-0.14023134398202	1.54653502513498	-2.54020138644954
H	0.67624169630663	0.00034784400814	-3.80633178193407

H	1.72673890646601	0.48075430806978	-2.45686400962071
N	-0.13156220485663	1.79140006058416	-1.06223141001532
H	0.31355901634482	2.41001570904776	-3.04974788055102
H	-2.18517667766281	2.20036952176628	-2.87488922453377
H	-1.47750201980656	1.21573347379594	-4.15652616525210
Rh	-0.00483445518890	-0.04502066629581	0.03863167334862
H	-0.90424384108402	2.39316296753461	-0.78651971300784
H	0.75026549071172	2.27219626489657	-0.84336518429641
H	1.66034976999164	0.34560346533739	-0.08797890349494
C	-1.85809857712388	-0.94488464691280	0.95416047653182
C	-1.51285861346720	0.26663841108697	1.65246554746220
C	-0.23451854041525	0.10575746173576	2.27160266241994
C	-2.39076164886251	1.46587064806639	1.78398951614017
C	0.25975917859576	-1.19806190688768	1.88943387375718
C	-0.73532635235212	-1.83971365611174	1.08002983944877
C	-0.67781083145504	-3.25129456261675	0.60025440521305
C	-3.21797682890692	-1.30951488141731	0.45833041792057
C	0.36255754389179	1.04168757497521	3.27087360399689
C	1.50281213431714	-1.86003719911603	2.38326557982731
H	0.28569339046910	2.08520417379628	2.95060570292434
H	1.41572563427063	0.82193062011705	3.45292995116593
H	-0.16595931339609	0.95257854474611	4.22979988728148
H	-2.99777709177550	1.61644786427495	0.88615914620983
H	-1.80232975055699	2.37075007831874	1.95898708390069
H	-3.07705708951303	1.34422240572758	2.63249712709230
H	-3.73875925609838	-1.89397635485330	1.22966979268320
H	-3.18571689667361	-1.92373740778911	-0.44521655878318

H	-3.82685082599461	-0.42627623264717	0.25108103781905
H	-1.32775806465027	-3.41024332361828	-0.26369579227124
H	-1.00874526774090	-3.93369979404725	1.39481166585225
H	0.33983952265817	-3.53686036800014	0.31997060152555
H	1.94902199891449	-2.49701607908046	1.61493038322494
H	1.26448851343493	-2.49716100497431	3.24590710217388
H	2.25262763416153	-1.13554506500865	2.70594719568373
C	3.78084964813155	-0.03960644484981	-0.69670398690384
C	3.13207829028603	1.50070782600434	1.23547783159234
C	2.91579663706964	1.13076425398680	-0.22708749269759
O	2.71176526542299	2.09848654875006	-1.03431760326360
H	2.37182273536548	2.21262597950087	1.56472729092052
H	4.11470260412558	1.98793994334545	1.30408328880099
H	3.13527057116078	0.63178546145183	1.89635041528549
H	3.50419250006131	-0.35875831216541	-1.70455947025887
H	3.71626296451898	-0.89293175359836	-0.01446467109141
H	4.82371968092488	0.30658727669784	-0.72125256126736

Charge: 0

Multiplicity: 1

Number of atoms: 45

Rh L2 / metal hydride

C	-2.43141076668337	1.74645696421999	-0.69539830722540
C	-3.11352626149696	0.58736570448498	0.03844716698212
C	-2.40736516029300	-0.74148349758061	-0.27595043089643
H	-4.17419957059711	0.53545572518815	-0.24396609099335

H	-3.07670866971322	0.76327242756066	1.12077718383148
N	-0.95101282517550	-0.72449228914243	-0.09386199954732
H	-2.84060606902412	-1.54297788713410	0.33434165053748
H	-2.62418716286091	-0.99874685638410	-1.33339162858039
C	-0.41214517845709	0.30241595018699	-0.98727683586291
C	-0.90939498423891	1.68492095443112	-0.55892472104328
H	-0.72875935867514	0.12203460409258	-2.03255488330631
H	0.68670121393681	0.28152571831438	-0.96102855705835
N	-0.50388638975991	1.83404075385133	0.87567631345108
H	-0.45167231774140	2.48879997979737	-1.15547479492336
H	-2.80967443164477	2.71521170953963	-0.34042084565531
H	-2.65987494100765	1.68979279816275	-1.76855023285481
Rh	-0.43122903221218	-0.08940548830305	1.85941007415682
H	-1.12190474719135	2.48685793603250	1.35388026293338
H	0.42710594291576	2.23585354912580	0.92606741476813
H	1.03158622451732	-0.18820079099828	1.27568292850547
C	-2.02867564394071	-1.06818889016742	3.24356752200578
C	-1.78875954537709	0.23737848160283	3.71482133118157
C	-0.37231439170663	0.36640212399353	4.02764916287366
C	-2.79657602589196	1.31842100337421	3.92680787011761
C	0.24494050733246	-0.89463242213314	3.76706002531413
C	-0.75541037924169	-1.77173647200478	3.19849312201790
C	-0.58545800774785	-3.22378276012016	2.88739231700034
C	-3.35926285337334	-1.67820243293732	2.94764342547600
C	0.25090250113132	1.55893955000166	4.67932329262621
C	1.64969252769502	-1.27808104701044	4.10613706870850
H	-0.15987530025584	2.49067224447829	4.27707554547585

H	1.33374339727680	1.57578506270078	4.52889693478357
H	0.06367801933195	1.55971857535808	5.76232453997040
H	-3.75192157406199	1.07913498360684	3.45294864946460
H	-2.44634424397812	2.27727405190140	3.52715733972633
H	-2.98120070949704	1.46727516460199	4.99905811483669
H	-3.81781924514450	-2.04332211702009	3.87685813208296
H	-3.26783630741040	-2.53447536653087	2.27484474428272
H	-4.05352585152054	-0.96214897288113	2.49835832979640
H	-1.20692549381457	-3.52277316002083	2.03751108957661
H	-0.87318191882741	-3.84911014085804	3.74434483811164
H	0.45363536430831	-3.45540052742528	2.63863020804820
H	2.03648937916047	-2.03220496684688	3.41627392961382
H	1.68995421521953	-1.69933936327700	5.11972078201182
H	2.32042606573660	-0.41548056783191	4.07606801767777

Charge: 0

Multiplicity: 1

Number of atoms: 62

Rh L2 / pro-R TS

C	-2.38682782163275	1.47333435992662	-2.56624661466973
C	-2.87583407569129	0.12469525624002	-2.03275686048413
C	-1.78842185369592	-0.95786272720878	-2.15000392739946
H	-3.78143140201509	-0.19083395247436	-2.56860561223216
H	-3.14942704313775	0.24044108241111	-0.97981315129601
N	-0.46474139496488	-0.56434167390930	-1.64353296963999
H	-2.11866982515610	-1.86942459752856	-1.63818616741996

H	-1.67957784227105	-1.22092642711524	-3.22078510743172
C	-0.04940569109246	0.61975459898955	-2.40006579230122
C	-0.97796337108180	1.78732076489106	-2.07029309060675
H	-0.08914715269482	0.42243943977074	-3.48693533658737
H	0.98084974174294	0.88220991962030	-2.14986645109121
N	-0.93495159035961	1.91240194758807	-0.57771936339489
H	-0.62249474327417	2.72640828725185	-2.52036892498197
H	-3.08413425848029	2.27632420957362	-2.28937809329357
H	-2.34325879244588	1.45367043852685	-3.66354319157211
Rh	-0.56657169769832	0.02818572528256	0.36170055931386
H	-1.75907747262961	2.39616156823553	-0.22816915252341
H	-0.10415980572086	2.47196209192960	-0.34125676349690
H	1.04443248068322	0.56567480993196	0.17789502150695
C	-2.30922751775929	-1.03663232209331	1.31867887639598
C	-1.95694252295448	0.12539899423407	2.09897525768428
C	-0.63317687337076	-0.03588804778253	2.60762868140966
C	-2.86797531554730	1.26837852979251	2.39829402441844
C	-0.12266266467505	-1.28159591292700	2.07748968789944
C	-1.15156570860520	-1.89146464740197	1.28901146074767
C	-1.07037820245424	-3.25161116138212	0.67970590308608
C	-3.68586328964295	-1.40940715047517	0.87882633988045
C	0.01709835510477	0.83188660152693	3.63412162894657
C	1.16061469624316	-1.94348332868602	2.45134185506552
H	-0.13605833203071	1.89546922828935	3.42713992073447
H	1.09170559754432	0.64869300459335	3.69177056511443
H	-0.40819211259711	0.62506806470997	4.62575161247497
H	-3.53542750155259	1.47733666062055	1.55660383755232

H	-2.30300412258224	2.17644332115478	2.62573000309262
H	-3.49553683764015	1.03722929315085	3.26914351553488
H	-4.13947567658667	-2.07271674943385	1.62853907310937
H	-3.69007618469824	-1.94612372272728	-0.07362944905775
H	-4.33312651394636	-0.53433125946327	0.78234386834127
H	-1.86225840403117	-3.41196176839749	-0.05533074808821
H	-1.17610154828995	-4.02014575043420	1.45692420001062
H	-0.10712747728655	-3.41132565867449	0.18580384466323
H	1.53439888093747	-2.57801114371212	1.64338381779918
H	0.99941924943172	-2.58614610555033	3.32792747746720
H	1.93576590475851	-1.22050084038494	2.71372647732666
C	3.05488708700980	0.67064506322248	-0.90027984653455
C	2.68410636221221	1.57006806341873	1.46335560268504
C	2.22885256208590	1.56014451686553	0.00919911128856
O	1.77874578545078	2.65132081781885	-0.46257497693518
C	4.13490393735710	-1.43572108667249	-1.41901583619691
C	4.66305289423357	-0.88776934711650	-2.58856649166913
C	4.38673010723137	0.43905576171936	-2.91180216176816
C	3.58504550647799	1.21135440891704	-2.07515173018435
C	3.34049294305606	-0.66071510117053	-0.58106024393174
H	2.91718947255276	-1.10064713308045	0.31757935169623
H	3.36067690179653	2.24477149262930	-2.32278971699667
H	4.79657757362257	0.87457468702999	-3.81946950484973
H	4.33935105079687	-2.47200662978742	-1.16291031282636
H	5.28408259120481	-1.49292120565595	-3.24340953924689
H	1.95720664169614	2.11255549550662	2.07021503165294
H	3.64064164921571	2.10953812466016	1.49580416169933

H 2.84344466784738 0.57359882121579 1.87890636011001

Charge: 0

Multiplicity: 1

Number of atoms: 62

Rh L2 / pro-S TS

C -2.36548817315122 2.55456062618804 -2.13512588394393

C -3.22241731441773 1.41236660418537 -1.57619547104376

C -2.80971299113443 0.05229317307516 -2.16296520972216

H -4.28476763714309 1.60809244933187 -1.77646556561533

H -3.10169507366916 1.37534901591810 -0.48970554889019

N -1.36374949877647 -0.20414671765867 -2.11315232709794

H -3.34662975839050 -0.75284790944243 -1.65157141870179

H -3.12878119749413 0.01821805047066 -3.22258766868910

C -0.72852978266728 0.83792888655939 -2.92807802340281

C -0.88645213643257 2.18535135869931 -2.23042622656024

H -1.19196500474239 0.88309695738453 -3.92943724135442

H 0.33557675277567 0.63444303391151 -3.05958875457057

N -0.25908284340029 1.97955641271054 -0.88831041912988

H -0.34184798909455 2.98042787556883 -2.76304821393709

H -2.49829012179827 3.46615838960443 -1.53584358532305

H -2.69192844159574 2.79706790712899 -3.15546065263740

Rh -0.60438655445039 -0.01142909081554 -0.17011920509612

H -0.52855305821491 2.71228813262518 -0.23625396272296

H 0.76174809305752 2.01923135954341 -1.03027348018405

H 1.02614775258373 -0.20878531700441 -0.85706844831022

C	-2.25430154837215	-0.88127450663778	1.08670083592041
C	-1.73959256671086	0.29556203423255	1.71932509218298
C	-0.34968016478250	0.06913519217856	2.05182607140474
C	-2.51727738245284	1.49199543253680	2.15307988051336
C	-0.00871350094069	-1.23406634852339	1.59096174353331
C	-1.16413817293665	-1.80954072165488	0.93418562976779
C	-1.26628774612664	-3.20246584891705	0.40692095055286
C	-3.69265973981401	-1.18239421827626	0.83536685046656
C	0.48503204822973	0.99994054509623	2.86701677211205
C	1.27042409994632	-1.95485549854729	1.82937268994202
H	0.36270993193938	2.03735545239144	2.53862537049096
H	1.54488762966501	0.74762191185707	2.80242518786461
H	0.18716003936477	0.95273313381602	3.92317599634806
H	-3.50890684753108	1.52598753988058	1.69710365969431
H	-1.99033367443912	2.42170123950645	1.91431972418547
H	-2.64935030866454	1.46575753368688	3.24308389250101
H	-4.14684869846884	-1.52459015356183	1.77561192610852
H	-3.81797367569104	-1.98254285961596	0.10271232268916
H	-4.25612925492898	-0.30796008414681	0.50089302977347
H	-1.97886286622328	-3.26031500256910	-0.42101808086420
H	-1.60617689347764	-3.89368605746345	1.19029032600446
H	-0.29802615801990	-3.55839415587193	0.04465305593632
H	1.56969846973283	-2.54750254601357	0.96105127513779
H	1.13881248847448	-2.64593243032695	2.67341192489233
H	2.08611318912931	-1.27139746528487	2.07015476583947
H	3.22649387113017	-1.23428212203798	-2.90238066434257
C	2.24308637668412	-1.15896563528212	-2.42023371618097

C	4.15839990769308	0.80631717192028	1.75810854284205
C	4.95250677445744	-0.32883388623100	1.93119825063718
C	3.28541053438787	0.88924438884626	0.68041900893451
C	3.16931272188392	-0.16065196063476	-0.23595446039258
C	4.85724916716797	-1.37371482929356	1.01756817017056
C	2.25553452315740	0.02226250478511	-1.44389639193282
C	3.96897978747881	-1.29243372956162	-0.05525411294435
O	2.27786912511569	1.21248028007014	-1.95335661766917
H	2.01961501385405	-2.10827777671447	-1.92316498443094
H	4.22238412356382	1.62800037200111	2.46719383684352
H	5.64051593545447	-0.39364956034988	2.76982081868864
H	2.67407887447853	1.77687743498698	0.54019658914958
H	5.47487002583402	-2.26016721022825	1.13731256110248
H	3.90999991781882	-2.12391810526902	-0.75014793133115
H	1.49491960109499	-0.99038065276298	-3.19700248520877

Cartesian coordinates for the optimised geometries for the Ru(L2) cycle

Charge: 1

Multiplicity: 1

Number of atoms: 45

Ru L2 / pre-catalyst (cycle starting point)

Ru	6.75725535136339	4.82516975512763	11.34920492377226
Cl	7.63286458655363	6.65459730947355	12.72340391414523
N	5.54648155750041	6.33532195818023	10.39873489054227
H	4.62603233169665	5.99238109379813	10.13196606024084
H	5.39478286114091	7.04589326561009	11.11361668209509
N	8.06447012657122	5.57532850767456	9.77200447148419
H	9.01818093577173	5.54963031330097	10.12851196867314
C	7.35792936518547	3.42532320872289	12.94074683275028
H	8.10122300158767	3.63878491092810	13.70034440158000
C	7.75060644231232	2.86215637524559	11.71159188519175
C	6.00882712604557	3.84123791028755	13.17280575488538
H	5.76618061128441	4.35513577476526	14.09737925558502
C	5.41440400895742	3.14726068240654	10.91613118808052

H	4.68955623298381	3.09202440729276	10.10893736165096
C	9.16007507732424	2.39846757863262	11.43148791661810
H	9.33825729432480	2.54689169529553	10.35759227580253
C	6.74443933829870	2.73341285594315	10.69465402740950
H	7.02596687917729	2.35017117726624	9.71807189128645
C	5.02243261253800	3.71173101285473	12.17595430066564
C	7.69075876111198	6.99552414100724	9.50669560436604
H	8.25266889115384	7.38319031027357	8.64670111311285
H	7.92182180367249	7.59701709061359	10.38606564280521
C	9.24287876684603	0.88962070138447	11.71672604017537
H	9.06198298237946	0.69400089860982	12.77989050947211
H	10.23944634624849	0.51519740898360	11.46158719015301
H	8.50422883944242	0.33094870846680	11.13255811213944
C	3.61954706514159	4.18037638497240	12.40266733983974
H	3.58664075315403	4.98279377271214	13.14376384303149
H	3.01870694251549	3.34314847077714	12.77827188733168
H	3.15848286570114	4.52474357633039	11.47273688731692
C	6.59672259869736	4.81129831634353	7.91959784961526
H	5.98411513567796	4.11803623894924	8.50105386067020
H	6.62966906834486	4.41904103728034	6.89733704822460
C	8.01622797175856	4.82991742928227	8.47583531443881
H	8.39182430134977	3.81635822102066	8.63684935310559
H	8.69660696597661	5.33126996206398	7.77179558456557
C	6.20479825383501	6.97819607812376	9.21020831337262
H	5.82526751207370	8.00197267388513	9.10323575762792
C	10.22893108062405	3.16668501548204	12.20735655875676
H	10.12446894444183	4.24854746059750	12.07003061113270

H 11.22079561581454 2.86860480992213 11.85445871912116

H 10.18164403632471 2.94820660764344 13.27971970925491

C 5.94476770264876 6.19949779961053 7.92113444873216

H 6.35825127099132 6.80579177310554 7.10581402093489

H 4.86687978345573 6.11229531975231 7.73876867824284

Charge: 0

Multiplicity: 1

Number of atoms: 44

Ru L2 / pre-catalyst NH_2-deprotonated

Ru 6.73681956699006 4.82349189961086 11.30908087266437

Cl 7.86783774599553 6.64880028668813 12.77720867701717

N 5.56117875153495 6.27642163009908 10.44880035960825

H 4.67392691791338 5.87040768234532 10.15542402690892

N 8.05451787171461 5.57873420016748 9.74541133100642

H 9.00805611837474 5.55869518287046 10.10188034202746

C 7.35034188544293 3.40171943835414 12.95543312997879

H 8.08028823047702 3.59633218346022 13.73219540110125

C 7.75240868008822 2.84489402612303 11.73084828744319

C 6.00811269850846 3.86557075566835 13.14754972984619

H 5.76392974040300 4.39533558736318 14.06351865164567

C 5.42324681314025 3.17523694995334 10.89088438199737

H 4.70924729164774 3.11634836089242 10.07427047924213

C 9.16231722517617 2.37381922105562 11.45698067055469

H 9.34034935407958 2.51807107489707 10.38176127914233

C 6.76634964902304 2.75902310884031 10.69174327329534

H	7.06434102733912	2.39032731321008	9.71376835701927
C	5.01931357524700	3.73298436326934	12.14966207605421
C	7.64452249445582	6.99418715317149	9.51303942773708
H	8.18732511564448	7.41103899329575	8.65242827358581
H	7.87319415613711	7.57402078202217	10.40773402406905
C	9.25853020011214	0.86831648303640	11.75071446463829
H	9.08481602308173	0.67846224259707	12.81641144799332
H	10.25463893286103	0.49332342511926	11.49245413673623
H	8.51731684567201	0.30247913972937	11.17635130338678
C	3.62089660019075	4.22250658553622	12.36455108567257
H	3.59678299945460	5.02441696494456	13.10739001492074
H	2.99178958647362	3.40104975847814	12.72873471391923
H	3.17990330090257	4.58782308301495	11.43254344772491
C	6.59061197334185	4.82218821800031	7.88978572567620
H	5.99709084275600	4.11181372931843	8.47049766813153
H	6.62502306993492	4.44284173862365	6.86152673080188
C	8.01291463014026	4.85497382983865	8.44120445201619
H	8.40384179349228	3.84332713521170	8.58621799647407
H	8.68235970108539	5.37488785322182	7.73830354335111
C	6.15085858611385	6.92673746232963	9.26506942895660
H	5.76024351079614	7.95520047532022	9.16442035212074
C	10.23030342972830	3.15388288167597	12.22310498433795
H	10.12095393912226	4.23278103354102	12.06771390529808
H	11.22522280334775	2.85225223705411	11.88051507574198
H	10.17805027176501	2.95477233892708	13.29925980482263
C	5.91429704002654	6.19834139938161	7.92736132468936
H	6.31150284789148	6.83186441346539	7.12100851090935

H 4.83726616237588 6.08817737827628 7.74518682973464

Charge: 0

Multiplicity: 1

Number of atoms: 44

Ru L2 / pre-catalyst ring N-deprotonated

Ru 6.76501118935632 4.82860792429055 11.31892770608815

Cl 7.52979898188631 6.76798734811806 12.80474703619282

N 5.52691019929603 6.32603125691990 10.34594751795583

H 4.62008146587504 5.96488155292032 10.05961337294248

H 5.35247176725617 7.02933888261962 11.06205197616837

N 8.04938474016187 5.51419515691618 9.83458169996158

C 7.36174500750274 3.45299759977808 12.93566836050509

H 8.11338030402486 3.67394516575528 13.68533221193493

C 7.75209892311579 2.88774562807823 11.70033405187479

C 6.00905141535674 3.82019572947557 13.20837876022451

H 5.77461408186327 4.31457650617978 14.14550835312462

C 5.40962377742971 3.19054343875876 10.93546521222896

H 4.67635634598023 3.14875943489742 10.13402006236502

C 9.16240565398519 2.43243195191877 11.40942293466304

H 9.34211018760045 2.62127251265618 10.34196173566559

C 6.73667080222964 2.75746540317740 10.69398277574980

H 6.99979020784392 2.36805102878862 9.71547678696225

C 5.02300666283454 3.72701209486548 12.21125258146677

C 7.70490243071357 6.90955596815640 9.53969224645794

H 8.30182613639422 7.28674667283102 8.69221565086500

H	7.91075432669590	7.54340957868277	10.40637754461786
C	9.26108654745598	0.91664775435372	11.64411012285383
H	9.07869963679252	0.68314144281532	12.69974917624215
H	10.26089420998584	0.55539382244405	11.38036547175730
H	8.52754510893775	0.37127811857611	11.04085825442348
C	3.61611243481724	4.18375391786886	12.45221225553516
H	3.57674201158352	4.93752070757336	13.24303055945260
H	2.99648605073347	3.33245931090476	12.76039891024761
H	3.17197364000366	4.59835416783679	11.54224881597634
C	6.59144028522640	4.79023753610766	7.90870659695102
H	5.93348576117147	4.11657048925253	8.46691662441886
H	6.65275187281000	4.39648188410967	6.88532392243724
C	7.98602647562078	4.78715198016424	8.55798572615167
H	8.33178647461768	3.75581917863580	8.69166480157600
H	8.69847063948613	5.26197137690736	7.85769709757693
C	6.22236361801949	6.96712066982166	9.18365173479938
H	5.86305432637866	8.00069371949157	9.07498829244061
C	10.22938422712454	3.18013059059243	12.20853615088069
H	10.12187420464695	4.26450257842923	12.09906404640840
H	11.22376658572027	2.89420846733941	11.85133448645664
H	10.17926614518788	2.93199596513813	13.27470428024128
C	5.97899871302964	6.19736079500899	7.88559702709787
H	6.44107744004387	6.78872988178926	7.08400041307471
H	4.90456898320337	6.14778480905442	7.66173665498424

Charge: 0

Multiplicity: 1

Number of atoms: 42

Ru L2 / bis-amide (active catalyst)

Ru	7.04410418878267	4.25046085307965	10.66915774561618
N	7.34818108292192	6.14882715530867	10.77816404806169
H	7.08581465330820	6.75146942749914	11.55387304246659
N	7.24713976265417	4.65341657897160	8.63062218826951
C	7.08786532551883	3.45665881081285	12.77173957503503
H	7.48630475792547	3.96317716328571	13.64426066781899
C	7.95553338203434	2.70280984987965	11.91992115934685
C	5.76782860689495	3.72250883608451	12.36257022613352
H	5.14864487344682	4.40140608159472	12.94324726092747
C	6.06661281035422	2.26349563721757	10.40553768914159
H	5.69798512210109	1.86464344831888	9.46467567593352
C	9.41632240221208	2.50676949197434	12.26141656121698
H	9.91357676911040	2.17663282472410	11.33925466202205
C	7.40593669910710	2.03938294970069	10.78035536603321
H	8.06131729731893	1.46407768648592	10.13377819532632
C	5.23858666913688	3.15212868382112	11.15068973448711
C	8.09618282776414	5.85839192948048	8.56375780438511
H	8.14555418946980	6.24990381988979	7.53164149235682
H	9.11915926703545	5.60425063499751	8.87993128605586
C	9.54215493065774	1.38394254920244	13.30370895086940
H	9.05596229384115	1.67700662845174	14.24167885934463
H	10.59712016233121	1.17948502152995	13.51544268445974
H	9.07483050181340	0.45906558268781	12.94959087643596
C	3.82130914143010	3.41581995041035	10.74150716291135

H	3.48348774572091	4.39282409122398	11.09773818333148
H	3.16399489231025	2.65265074764971	11.17827264317262
H	3.70845411281011	3.37493051871430	9.65465950732341
C	5.21642126762018	6.17192260023111	8.69260760223171
H	4.81313894546743	5.81838320513050	9.64891844716999
H	4.36044593058780	6.48934096555158	8.08042005916028
C	5.95529653940317	5.00176446401031	8.01440068081876
H	5.30579933600286	4.11512787991943	8.00880801411434
H	6.13588027647573	5.27128307389055	6.95499888818114
C	7.50650733235802	6.88617881906791	9.51242283955314
H	8.17367728870034	7.75345676687928	9.65329810062547
C	10.10924862502176	3.78272355721480	12.74353403398800
H	9.97155761423056	4.59729438122001	12.02397158057540
H	11.18207341636743	3.59943756012987	12.86351893585348
H	9.72068157164497	4.11069435421476	13.71388109858192
C	6.15010691110538	7.36209862701368	8.95503347690899
H	6.34555146549579	7.90516402366764	8.01933525619017
H	5.67783901150561	8.07369276886103	9.64612773756326

Charge: 0

Multiplicity: 1

Number of atoms: 54

Ru L2 / IPA TS

Ru	-0.40994924891155	-0.09515712441026	0.07297187293605
N	-1.63843482788211	1.43262838809536	-0.81026931462959
H	-2.60003428741477	1.15402589363600	-0.99004816296842

N	0.84169155324167	0.54276614830294	-1.48291787913632
C	0.20973380985927	-1.32371547498081	1.77865876178346
H	0.92352698958763	-1.02554417540882	2.53950958827580
C	0.66764166797291	-1.95442376553421	0.59442498250015
C	-1.16954084583700	-1.01850399139464	1.97885041461826
H	-1.47986544979384	-0.49797260948987	2.87772278577643
C	-1.66741463254314	-1.92275662423750	-0.24028692196940
H	-2.37876595536322	-2.11586544904026	-1.03827264844425
C	2.11193093512403	-2.33136875548535	0.35924728810793
H	2.29571301339805	-2.20342111275221	-0.71761274777609
C	-0.31491358085687	-2.26876521526308	-0.40381098627246
H	0.00643084892738	-2.72837832185807	-1.33417834286648
C	-2.10598975981150	-1.24267861610156	0.94657218231710
C	0.48485352322664	1.93119635170164	-1.78256325884137
H	1.63628995106459	-4.45413570259880	0.13323695869633
H	3.34649441655926	-4.12112152475348	0.47614603972342
C	2.31937537608427	-3.81467275537964	0.70259470380301
H	2.13762821814335	-3.98589303355898	1.77042378613100
H	-3.97584318058626	-0.55740800379327	0.13388305014858
H	-4.13120839611340	-1.66946987632510	1.49757004589911
C	-3.54107468737974	-0.83469146996800	1.09915790812342
H	-3.63793492118272	0.00950572819884	1.78773161186722
H	-0.76726686041010	-0.63488178869130	-4.30193980627552
H	-1.37064501098048	-0.87471560799669	-2.66378652173776
C	-0.75388842299010	-0.21849114855882	-3.28531670456773
H	1.34609909242149	0.23764396152965	-3.49692956388487
H	1.02965924345965	-1.24475108705480	-2.59586222838148

C	0.68220857270418	-0.21363746237751	-2.73415038617529
H	3.05360205490452	-1.62300709258186	2.19604131463873
H	-1.36114331034837	3.03923979854260	-2.17639467127825
C	-1.01549692999968	2.00221735759489	-2.04760344061639
H	4.12812497161543	-1.69050056090289	0.79593625918278
C	3.10822914368987	-1.45250933924195	1.11474843402146
H	2.92376085406047	-0.39038084825758	0.92209936387703
H	-2.44891441074678	1.14430375592485	-3.43668398474804
H	-0.95928626563366	1.75706955534022	-4.15045210249739
C	-1.35980827841346	1.19224587713930	-3.29762642354533
H	1.03322309379926	2.29951933289645	-2.66808075904401
H	0.73195049575760	2.58209713889727	-0.94011714678885
H	0.18081888288506	1.29319281241506	0.89917570026458
H	-1.65057384539348	2.18213008233771	-0.10561333073571
C	1.68532658248007	2.79676527139832	1.62666704092925
C	-0.38356639529549	2.13601118800044	2.96768058705621
C	0.16710262766473	2.61855782630822	1.63026047882190
O	-0.54507366738148	3.43396832548736	0.96317632690874
H	-1.44554379096850	1.89483614076188	2.87788214538260
H	-0.27600232837519	2.96661175680974	3.67922186592678
H	0.16098740135531	1.27343185073135	3.36109542204267
H	2.06253876293887	2.98720071601419	0.61911272448883
H	2.19884310300211	1.92837418020630	2.04996346148789
H	1.91439410468518	3.67327909972682	2.24875422744428

Charge: 0

Multiplicity: 1

Number of atoms: 44

Ru L2 / metal hydride

Ru	6.77846095139638	4.82283604592278	11.30906195322014
H	7.44491203438631	6.06831111276258	12.08621174160287
N	5.56952405031011	6.38741861078219	10.41248537178517
H	4.65491473126983	6.05407279265458	10.11671494466265
H	5.39548328033536	7.07952098457862	11.13503340891843
N	8.08055353253721	5.49534884057378	9.78436258064402
C	7.36613895629513	3.51250317562950	12.95018296536261
H	8.10667107058575	3.74669212994970	13.70711032659096
C	7.76987802571624	2.89765679111092	11.73674426704182
C	6.00655318542545	3.88930048133320	13.17498934962667
H	5.73002653446327	4.40706195482144	14.08807297063842
C	5.42275988677470	2.99249100284845	10.96087241699897
H	4.68420404356903	2.82701751009983	10.18100950598674
C	9.18904914540597	2.44927260998307	11.46698428408632
H	9.37360875688290	2.62733378840627	10.39717082512126
C	6.75099138937179	2.60999176556379	10.76076277650157
H	7.03699688864181	2.14022522794863	9.82300535347240
C	5.04867743153922	3.71837479638608	12.14603852664186
C	7.76351474877860	6.90530547531149	9.56007796797206
H	8.33677197513637	7.31385563188402	8.70579041698633
H	8.02011106674803	7.49535315135797	10.45074545090916
C	9.30929412054578	0.93855509254170	11.72276294768799
H	9.12541214906913	0.71765001813990	12.78116075360735
H	10.31417679571984	0.58355460946588	11.46842605217462

H	8.58477557761779	0.37400961688873	11.12610470283108
C	3.63366306207324	4.18686507380448	12.32482831084580
H	3.58248497022489	5.02217251377892	13.02893482410478
H	3.00946219846103	3.37318801629573	12.71575594531524
H	3.19926232378173	4.50237351017257	11.37091429213662
C	6.46447790691673	4.88182594948322	7.93516037653065
H	5.80333311262370	4.25146227820804	8.54083241182099
H	6.43765460735072	4.48589721318871	6.91038821479368
C	7.89163827446289	4.80009376020761	8.50367459744115
H	8.18536463708891	3.74780392307135	8.60975719522851
H	8.58197704684178	5.24452974321609	7.75777474866190
C	6.27148666475254	7.04329379619717	9.25974280100447
H	5.95476060855940	8.09521795423065	9.19239416269455
C	10.24647330747915	3.21574458750504	12.26049439476472
H	10.13395699133080	4.29726388224527	12.13253239299172
H	11.24613903090634	2.92950584296857	11.91771329526494
H	10.18788907283293	2.98557876576792	13.33052628522374
C	5.95044148163660	6.32682949337155	7.94674443903605
H	6.43906777681862	6.89965948308245	7.14656873190570
H	4.87062659733535	6.35987099625918	7.74497071916243

Charge: 0

Multiplicity: 1

Number of atoms: 61

Ru L2 / pro-R TS

Ru	-0.81524533060941	-0.54714916818117	-0.01388379315628

N	-2.15844897382422	0.91079619158924	-0.83783770657594
H	-3.09360776036191	0.55788728306551	-1.02673507325796
N	0.35861315242997	0.20326800349010	-1.57074256565585
C	-0.01191301131278	-1.82870464476529	1.58131193589651
H	0.76368619795974	-1.55106400963837	2.28724837893730
C	0.33951133869905	-2.40894879511042	0.33915621550374
C	-1.37366013174755	-1.55281684855806	1.91505118576699
H	-1.60595073104700	-1.07180908856774	2.85848713827290
C	-2.05960699148797	-2.37539005956369	-0.28023943003330
H	-2.83647367107142	-2.53709207544363	-1.02299589979235
C	1.75785755958165	-2.77699570355818	-0.03162091862356
H	1.84485704150924	-2.63832588976420	-1.11861221028414
C	-0.72446249387658	-2.70053988866739	-0.58082227299109
H	-0.48391756870622	-3.11975071256211	-1.55368452745164
C	-2.40269616703480	-1.76715994300141	0.97464679049433
C	-0.08187618900566	1.57741161180553	-1.81815136448880
H	0.42727560766560	2.00559540424862	-2.70023843461899
H	0.15379491795645	2.21145957352738	-0.95873101689060
C	1.99019310951801	-4.26521423987662	0.27496098522972
H	1.90864381747136	-4.44751344450375	1.35321773509807
H	2.99064828354521	-4.57099861301228	-0.05002638608318
H	1.25474316544653	-4.89678157732891	-0.23480082855688
C	-3.82458975714899	-1.40011930222073	1.27938232427165
H	-3.87153343844362	-0.59617200821352	2.01945910321079
H	-4.36245191663442	-2.26718950505022	1.68278727335604
H	-4.35082793502156	-1.07955832917943	0.37496961133052
C	-1.22642274144089	-0.59377940682941	-3.36436693040291

H	-1.79676819077130	-1.29544788448165	-2.74705195593724
H	-1.23784415274652	-0.98727787858668	-4.39004685179511
C	0.21833570841421	-0.52675276533681	-2.83948563564391
H	0.62037594144397	-1.54169166572235	-2.73433520829775
H	0.84269846300688	-0.02121504740224	-3.60149035452824
C	-1.58876474909619	1.56782733282372	-2.05716830912540
H	-1.99830319132248	2.58583899725909	-2.14070161281373
C	2.82214377013169	-1.91016057830855	0.63916748910044
H	2.64210309452644	-0.84689929392452	0.45229516715044
H	3.81018795403867	-2.16719869054520	0.24357969155924
H	2.85005765316240	-2.07223903726948	1.72283643604708
C	-1.90289024517167	0.78362754284395	-3.33141116756546
H	-1.53837718193914	1.39349938117654	-4.16913198231062
H	-2.98932896030361	0.68447204481399	-3.46380762277897
H	-2.23176898258215	1.62962449306309	-0.10532651023699
H	-0.29946694774678	0.85260390289948	0.83351291613271
C	1.01619602103332	2.61683653226444	1.35947253907858
C	-0.70237319644349	1.62428136166267	2.98105815717740
C	-0.42461614600172	2.21151718883010	1.60190428245006
O	-1.34426080056053	2.89435843582677	1.05937451270352
C	3.40044300501702	2.21159962891972	1.51383757717310
C	3.65862038496669	3.45881153024001	0.94401358193422
C	2.59497793649870	4.28359936928466	0.58381766239716
C	1.28295481357297	3.86353378553124	0.78696138587901
C	2.08986934616684	1.79680639101035	1.72271988603391
H	1.89885423385390	0.81578714901631	2.14917261086658
H	0.44804912135446	4.49856790760506	0.50525593881518

H	2.78750845254625	5.25807359472596	0.14245745124354
H	4.22388823760125	1.55859716403477	1.79142740793944
H	4.68273601530832	3.78348544984233	0.78118370063951
H	-1.73015377357268	1.25647325541954	3.01850681165813
H	-0.59572876421260	2.44198826624843	3.70686185015531
H	-0.01549425318096	0.82372732210537	3.26335083639381

Charge: 0

Multiplicity: 1

Number of atoms: 61

Ru L2 / pro-S TS

Ru	-0.22500331822034	-0.24942340648585	-0.50237703151502
N	-1.42480519209374	1.27641285694126	-1.42523054417263
H	-2.38470616049828	1.00122873068023	-1.61865806996169
N	1.05233016055984	0.35542228612716	-2.03672944944553
C	0.35884696315703	-1.45806099302697	1.23293518949798
H	1.06687801594294	-1.15255007875309	1.99605219755754
C	0.82031474867778	-2.11471696582407	0.06471103278354
C	-1.01627309239934	-1.12803869344065	1.41507222023991
H	-1.31970733591726	-0.57369949286815	2.29642512628796
C	-1.50445037217326	-2.04667508275814	-0.79809564247446
H	-2.20927544870650	-2.23336136452026	-1.60350631508759
C	2.26216646968781	-2.51401876727990	-0.14648038657848
H	2.46322758350568	-2.40047786050560	-1.22179585238163
C	-0.15459104343523	-2.42029532325266	-0.94333344163411
H	0.16881924102364	-2.89397666081504	-1.86585864950118

C	-1.94768307991072	-1.35501385710131	0.38042346691505
C	0.71982943674357	1.74448851048073	-2.36015560449137
H	1.29158126153534	2.09632399863417	-3.23700854325955
H	0.95652079878723	2.40181808091442	-1.51981643053949
C	2.44360210962422	-3.99602528166498	0.21653854546416
H	2.24276370801886	-4.15240021498814	1.28321253368162
H	3.46987623272701	-4.31871578579962	0.00985118854989
H	1.76080856923641	-4.63248118274670	-0.35646420707098
C	-3.37645643160360	-0.92088115113249	0.51595058092223
H	-3.46057560335807	-0.06168560781877	1.18778609198708
H	-3.98186801365354	-1.73723306460334	0.92926210497843
H	-3.80228640001234	-0.65623218081289	-0.45682840547838
C	-0.50460238813552	-0.41358293395936	-3.86350924550773
H	-1.13881893487129	-1.05871468009958	-3.24776826001430
H	-0.50020676121312	-0.84095861097617	-4.87553601588448
C	0.91974132922496	-0.41464711598840	-3.28252319202414
H	1.25495221036305	-1.44669638822271	-3.12374233910437
H	1.60474273325795	0.02259895471882	-4.03415066552014
C	-0.77410872853016	1.82493125881931	-2.65727615129000
H	-1.10737580940592	2.86319739693879	-2.80573414533629
C	3.25884551659486	-1.64036317148721	0.61486769469120
H	3.09168778341670	-0.57770734382681	0.40924614449480
H	4.28003024205123	-1.89487498906109	0.31352640928083
H	3.18607774664197	-1.79912810713153	1.69689889209806
C	-1.09921229689478	1.00166954505517	-3.90379178396584
H	-0.67812096659819	1.55311425860254	-4.75535075157406
H	-2.18566363656870	0.95957403801909	-4.06349591923459

C	-0.30334110358262	1.96742189874721	2.33389299055297
C	-1.66958499645566	2.16506930543864	2.55438102373000
C	-2.27426967165776	1.72343629482084	3.72649502605282
H	-3.33941794422035	1.87979851179718	3.87734455564152
C	-1.51758784764517	1.08297004799949	4.70747329682603
H	-1.98750537150113	0.73751935096128	5.62430335281757
C	-0.15232880359070	0.89376408360825	4.50382498120034
H	0.44824792329741	0.40103446141017	5.26402859265348
H	-2.25218081481542	2.66559045366421	1.78667862998502
C	0.45047544558132	1.33281904031727	3.32667663945801
H	1.51425158532511	1.16618445105093	3.18803206227175
C	0.30515678129359	2.46999643450672	1.03332003240811
H	0.35139564455182	1.16659213277899	0.34041606023123
O	-0.39992566423906	3.28003618679641	0.34218992543042
C	1.81857156558070	2.69788567121373	1.06008375056774
H	2.38563990146304	1.81478918233459	1.36410846146390
H	2.02903755643928	3.50991847277837	1.76884016787828
H	2.15812036947807	3.00963990924239	0.07032888204681
H	-1.44260640188064	2.03739055155311	-0.72996080759823

Geometries of small molecules used in the calculations

Charge: 0

Multiplicity: 1

Number of atoms: 10

Acetone

C	-1.53199231257785	0.56981829310391	-0.09250308063316
C	-0.24704675195897	1.35297897959610	-0.14227297014728

C	1.01492523653642	0.58800866062902	0.15630514588980
H	1.02158453766429	0.32671616929075	1.22220296129708
H	1.89810511427305	1.18562626199849	-0.07613511221832
H	1.03237575554958	-0.35665886365916	-0.39802896990451
O	-0.22936686951558	2.54553793381170	-0.41044024457336
H	-2.39636350733920	1.23407484019427	-0.14539087393208
H	-1.57306920968972	-0.04425038785431	0.81373598656934
H	-1.55228199294203	-0.12363188711079	-0.94292284234749

Charge: 0

Multiplicity: 1

Number of atoms: 17

Acetophenone

C	-1.55710436670092	0.57658062478363	-0.07390027752903
C	-0.26058318186238	1.31366051887342	-0.13829939975134
C	1.01542495077064	0.56316447623699	0.14017555152716
H	0.99337691071387	0.12559340307229	1.14468158758951
H	1.86286754612631	1.24455801483211	0.05548287268424
H	1.13664932888719	-0.26434649337636	-0.56803730322220
O	-0.23740610277282	2.50910440993894	-0.41298754409040
C	-1.61799947389096	-0.78944437418136	0.23219457091965
C	-2.84520927339767	-1.44255205626640	0.28409706881249
C	-4.02134105390643	-0.74015461565452	0.03164487131357
C	-3.96945096265192	0.62048144948707	-0.27440467324502
C	-2.74654888532243	1.27407207103211	-0.32684886948577
H	-0.71079917860477	-1.35101241339174	0.43131988181537

H	-2.88362869797878	-2.50159565687579	0.52228728582421
H	-4.97907050064264	-1.25171315105014	0.07302112388758
H	-4.88635155784710	1.16871384995094	-0.47153061437385
H	-2.69447550091919	2.33225994258885	-0.56375613267617

Charge: 0

Multiplicity: 1

Number of atoms: 12

2-propanol

C	-1.51999571980329	0.57808178124363	-0.14972459719060
C	-0.24689279376904	1.38471442673423	-0.36600395383720
C	0.98766388737156	0.65714068271998	0.13347882331871
H	0.89266212658003	0.44096163785107	1.20388769592907
H	1.88571544342009	1.26317397446944	-0.02257982138617
H	1.11118709802697	-0.28962485867774	-0.40126147456882
O	-0.30458335829621	2.63783070798463	0.35022914503694
H	-2.39986588732839	1.13957729357344	-0.48421379437296
H	-1.64157181451450	0.33826725531309	0.91287119560791
H	-1.48086331871660	-0.35886432150384	-0.71619839891992
H	-1.08435431972799	3.11587444954812	0.04086490157735
H	-0.13075134324261	1.59462697074393	-1.44236972119432

Charge: 0

Multiplicity: 1

Number of atoms: 15

***t*-butanol**

C -1.52852205755637 0.54172440334506 -0.03683192582792

C -0.25554297012650 1.35438986685988 -0.27332278975786

C 0.96780155512004 0.61434174864473 0.25223924111430

H 0.85759046904933 0.40442277395963 1.32138330654655

H 1.87104031205140 1.21559019057778 0.10418509178905

H 1.09198344283934 -0.33529668834416 -0.27709275841808

O -0.31878265588499 2.58288871015349 0.49503657491954

H -2.40879699331934 1.10137218071748 -0.37507004793564

H -1.64508447840637 0.31760407181284 1.02869889931376

H -1.49326174435977 -0.40317498106701 -0.58984810653185

H -1.08466964641831 3.08100380254060 0.18038712883296

C -0.09506626159727 1.69055865895719 -1.75585241092952

H -0.02099853987068 0.77613381601837 -2.35446515881495

H 0.81006721206140 2.28573800795656 -1.91608382085456

H -0.95863764358191 2.26300343786753 -2.11507322344577

Charge: 0

Multiplicity: 1

Number of atoms: 19

***(R)*-1-phenylmethanol**

C -1.66083823211438 -0.43583472536195 -0.14797928131020

C -2.30983001733907 -0.32097806178567 1.22352358816825

O -2.31374315943675 0.52773966458251 -0.99465444687969

C -0.16549115980521 -0.20234091324805 -0.08494729613914

C 0.34667256632393 1.08269731150035 0.12025404706936

C	0.72169764363134	-1.27223243918031	-0.21186440138057
C	1.72023726116090	1.28970940362365	0.20158137307961
H	-0.33657305444455	1.92312704108798	0.21230117993384
C	2.09811888276386	-1.06769494262219	-0.12623024055263
H	2.10684271813305	2.29307530564907	0.35995906875161
C	2.60077557794614	0.21446871205357	0.07974020638877
H	3.67328937077535	0.37760014837654	0.14156398462440
H	0.33160269023250	-2.27386990089012	-0.38133105313396
H	2.77755211689465	-1.90967705991220	-0.22867305964634
H	-1.87229917041357	-1.05268971439192	1.90907636616559
H	-2.14945068929053	0.68164884306791	1.63459016580911
H	-3.38624245166384	-0.50685961299795	1.15056157746256
H	-1.88977364890378	0.48836765685037	-1.86189622654440
H	-1.83477724445003	-1.44964671640157	-0.54312555186615

Charge: 0

Multiplicity: 1

Number of atoms: 19

***(S)*-1-phenylmethanol**

C	1.66092797592296	-0.43566575938987	-0.14874260078096
O	2.31361200913190	0.52891717385026	-0.99445178526416
C	0.16558350198137	-0.20231077448177	-0.08517361806669
C	-0.72160330207448	-1.27201611885786	-0.21364673977716
C	-0.34658068637229	1.08240964797916	0.12202304857936
C	-2.09802241527100	-1.06762635040368	-0.12759590182638
H	-0.33150893746923	-2.27339037869233	-0.38466285075842

C	-1.72013919582606	1.28928032011661	0.20377696435399
H	-2.77745233906952	-1.90946118343801	-0.23126324141729
C	-2.60067618268630	0.21421599858461	0.08036760317296
H	-3.67318662959174	0.37724195356295	0.14252671296142
H	0.33665704489516	1.92271067057759	0.21529400437289
H	-2.10674460332225	2.29239935575620	0.36371179823075
H	1.88945625424286	0.49051686263167	-1.86164631772372
C	2.31019573127205	-0.32235944276773	1.22275667999326
H	1.87283780688299	-1.05488133217848	1.90755453495608
H	2.14986636556859	0.67978331802173	1.63501889001343
H	3.38660043117881	-0.50811277066115	1.14935818499645
H	1.83483717060617	-1.44901119020990	-0.54509536601583

Charge: -1

Multiplicity: 1

Number of atoms: 14

***t*-butoxide anion**

C	-1.51831318412010	0.54838651492235	-0.03337728482611
C	-0.25581853165154	1.43210353737745	-0.22122659135187
C	0.96986408312385	0.60076885260567	0.24416948450090
H	0.87721940358976	0.37212467363689	1.31367857657814
H	1.88646729103884	1.18785212762763	0.10296907894536
H	1.08300661258160	-0.34824254689240	-0.30040896367013
O	-0.36319414237407	2.60882436335486	0.47595008650212
H	-2.40484079598676	1.10439544246084	-0.36481529047789
H	-1.64803192052872	0.31372996777938	1.03104305202571

H	-1.47732350789349	-0.39870454636659	-0.59093579029850
C	-0.09116414663050	1.68890721554268	-1.74413305984254
H	-0.01634081633993	0.76602827202617	-2.33772541741081
H	0.81375298956556	2.28444500271408	-1.92112954323467
H	-0.94971333437449	2.26523112321098	-2.11244833743967

Cartesian coordinates for the diastereomers for the Ir complexes

Charge: 1

Multiplicity: 1

Number of atoms: 43

S_C,R_N,R_{Ir} - [(η^5-C_5Me_5)Ir(L1)Cl]$^+$

Ir -0.55736737872828 -0.59254898890000 1.92013328097574

Cl 1.28991111776805 -1.76152162433729 0.79581722907884

C -1.93086968290285 -1.44685739992125 3.37618838080496

C	-1.89754620960219	-0.00346694319927	3.54060944833524
C	-0.53480943107609	0.35122782720604	3.89710033138893
C	-3.09445366172316	0.88256217130015	3.59897688115624
C	0.25665799910850	-0.84249560057359	3.92783925942397
C	-0.61727257539842	-1.96790883385342	3.60946836656678
C	-0.21788769036322	-3.40176753903009	3.62250763293623
C	-3.14898987447155	-2.24510024084920	3.06641089161081
C	-0.04409737188567	1.71403724295047	4.24552128725721
C	1.68840641376137	-0.94667747675463	4.32190276196460
H	-0.68480924496807	2.49927402143790	3.84000941806987
H	0.97664648339559	1.87559054745413	3.89013868127308
H	-0.03968052898244	1.82174821611607	5.33752149785254
H	-3.86058998527533	0.57319475766453	2.88409015091712
H	-2.84510152262627	1.92837716635417	3.40830687661837
H	-3.53501612560688	0.82825261887804	4.60306412384098
H	-3.64750461686261	-2.52103032482790	4.00412110969452
H	-2.90165971137784	-3.16581070678715	2.53287615320013
H	-3.86330128636952	-1.67238665625006	2.46886330749833
H	-0.86484927231088	-4.00373638959292	2.98034121498523
H	-0.29325057967822	-3.79258137818788	4.64518203270557
H	0.81343918670059	-3.52710378149776	3.28584101474984
H	2.19353965628911	-1.74043437768156	3.76690025148602
H	1.75724108290208	-1.18191210047664	5.39165729032451
H	2.22091304285595	-0.00940080047713	4.14553827894455
C	-2.36136407646020	1.40342677978798	-0.08671423365903
C	-1.23657582322050	2.11217812975318	0.70266723756947
N	-0.11003923943298	1.12882950328994	0.63253319404669

C	-1.64820675149083	0.22874444998645	-0.78472036122597
C	-0.19733887040899	0.68009024960313	-0.78917708962092
N	-1.69011091103609	-0.97659428915723	0.09983489715392
H	-2.04769423892508	-0.00180389925211	-1.77678279869230
H	0.52419924815202	-0.10769556863008	-1.00586357074727
H	-0.05115408374415	1.52935442139697	-1.46920163794953
H	0.78684485484810	1.55570153097340	0.85121962322281
H	-2.79783216774031	2.07422605186545	-0.83034637564900
H	-3.16674978048921	1.05065575837481	0.56395044262287
H	-1.49171216785495	2.33491528988232	1.73676762178090
H	-0.93162067960269	3.04160766290724	0.20468504120548
H	-1.26369743999408	-1.76956566945867	-0.37936796099646
H	-2.65450610517174	-1.23173380748653	0.29978881727792

Charge: 1

Multiplicity: 1

Number of atoms: 43

S_C,R_N,S_{Ir} - [(η^5- C_5Me_5)Ir(L1)Cl]$^+$

Ir	-0.44778447882508	-0.47252044477039	2.00261381766287
Cl	1.70991957846639	-1.05424064457025	0.99843605540042
C	-1.92644453749560	-1.31943991996217	3.34482904411344
C	-1.78926914422343	0.09303355333350	3.60909822840666
C	-0.41265616477846	0.32366989414612	4.03554427131123
C	-2.87677263477361	1.11142211309022	3.62524121677112
C	0.27250192072916	-0.93142956054402	4.02752249866900
C	-0.65016406345074	-1.95983735208938	3.57022987926486

C	-0.36176509559138	-3.42100413708535	3.51118771112463
C	-3.18108343375579	-2.00681764049917	2.93272051644268
C	0.13723578023660	1.63375572800403	4.48255075621657
C	1.67639750328336	-1.16924522942144	4.45640943272136
H	-0.30228399034159	2.46322459910308	3.92195331532791
H	1.22263665297141	1.67293806765701	4.36923853408526
H	-0.10071414778286	1.78688632071679	5.54284156086219
H	-3.72764104309041	0.80528509265001	3.01251434535545
H	-2.52225314581327	2.08225469922890	3.26711622762541
H	-3.23538843941693	1.24807537213737	4.65342457960248
H	-3.74157952348059	-2.30137141458438	3.82857228144826
H	-2.97184998955611	-2.91363087022779	2.35943839537314
H	-3.82449596741332	-1.35262468415099	2.33790771836816
H	-1.01848943159385	-3.92930130792342	2.80135049781732
H	-0.51303584834081	-3.87475552884722	4.49892016715058
H	0.67248387214750	-3.60536411100161	3.21051413031034
H	2.13322583758806	-1.98291630592006	3.89017929112380
H	1.67930751782041	-1.44633401551462	5.51841457303979
H	2.29188005382809	-0.27571869654469	4.33468020297523
C	-0.28823332286493	0.29143200528593	-1.43820216512589
C	-0.75892686842334	-1.13652732367809	-1.08238044649520
N	-1.55851272803923	-0.92796568968886	0.16675070307034
C	-1.06460150897755	1.20326944320282	-0.46947142128350
C	-2.27645108733379	0.34999710120875	-0.13005612634183
N	-0.30712519491032	1.36340869029024	0.81200609171128
H	-1.31280656959399	2.17919596103484	-0.89702114605978
H	-2.84932520800824	0.69627835195048	0.73347065015246

H	-2.93636182550927	0.23589612027018	-1.00074333677185
H	-2.20180862333947	-1.69935439093764	0.32432929234382
H	-0.55461941889051	0.54402938822757	-2.46735503150246
H	0.79241320141908	0.40712937458670	-1.32488042343757
H	0.05681357049673	-1.83474185735619	-0.89886129054420
H	-1.42378279176510	-1.53907145849088	-1.85647309306694
H	-0.69321133919369	2.14049159308222	1.34433342950412
H	0.66792207758653	1.58803911460188	0.62077506527687

Charge: 1

Multiplicity: 1

Number of atoms: 46

R_C,S_N,S_{Ir} - [(η^5- C_5Me_5)Ir(L2)Cl]$^+$

Ir	-0.42054624362992	-0.41223684892304	1.95167193453949
Cl	1.81768431663300	-0.92086763272282	1.07725823614648
C	-1.98071892053547	-1.20687661982658	3.29150070228884
C	-1.75462256261931	0.18262811805668	3.58517552285334
C	-0.36406205374675	0.32809143185585	3.99437777694990
C	-2.77296670287157	1.26752553067667	3.67258392915150
C	0.23780756442287	-0.97164712042634	3.97056143219761
C	-0.73714650092750	-1.92623090735794	3.48329123109783
C	-0.55007191321742	-3.40249077832664	3.39197595669592
C	-3.29507264984748	-1.84067663604218	3.00026215965286
C	0.26246699762340	1.59365772890450	4.47012195796178
C	1.62118118785326	-1.29621113955890	4.40642761015787
H	-0.14333600623817	2.45887637413092	3.93869613104046

H	1.34597484200134	1.57940300939304	4.33238962579678
H	0.05709033111214	1.73142666014358	5.53924340774024
H	-3.71863730620700	0.98634475937183	3.20592323982573
H	-2.41746717524208	2.19553270880560	3.21448739904676
H	-2.97202192677774	1.48056698377449	4.73063372571896
H	-3.74992748173550	-2.13361285723238	3.95575576503820
H	-3.18520589163517	-2.74624033242425	2.39954004265810
H	-3.98772654254098	-1.16069559755035	2.49984803878375
H	-1.20192847127844	-3.83782628105756	2.63056285693042
H	-0.78878799288412	-3.87573883983664	4.35305628010211
H	0.48295009731782	-3.65335809293556	3.13948156108573
H	2.02219557446685	-2.15761520208899	3.87030147767943
H	1.59520552707350	-1.53911507173597	5.47677157238483
H	2.29854101472245	-0.45189998569724	4.26518431727193
C	-2.32109665136586	1.52442225953033	-0.66586467246957
C	-3.11139216193434	0.42576062286038	0.05518648762964
C	-2.58638671206832	-0.96928409329110	-0.25808694093186
H	-4.16892239966950	0.48020895669499	-0.22550092270976
H	-3.05717172865386	0.58885178173940	1.13411225919859
N	-1.10751722809711	-1.01157115788049	-0.05763789990387
H	-2.79936346933631	-1.23850000446019	-1.30301164680241
H	-3.04886461127776	-1.72108020190230	0.38460134992541
C	-0.80858025933185	1.30766894856010	-0.60414279934910
H	-2.58321333026635	2.50924954604530	-0.26043947523751
H	-2.58847807871618	1.53710084158825	-1.72981049787019
C	-0.47444610386638	-0.10087509768243	-1.05661679386406
N	-0.27321331764362	1.43507600513243	0.79317013761688

H -0.30584864371351 2.04045256779719 -1.24744017368641

H 0.60180373829374 -0.27657663864133 -1.07037995053424

H -0.88495108162792 -0.29222799877644 -2.05644417799317

H -0.77546310002290 -1.95976081309666 -0.22054554552825

H 0.71930789385864 1.66044770297698 0.74497368721014

H -0.72407386585255 2.21181341143585 1.27231368450277

Charge: 1

Multiplicity: 1

Number of atoms: 46

R_C,S_N,R_{Ir} - [(η^5- C_5Me_5)Ir(L2)Cl]$^+$

Ir -0.45229646575517 -0.42108429598164 1.98691696192176

Cl 1.76226241045657 -0.81248553288424 1.04076829103024

C -1.94918028295551 -1.21219015430693 3.34587727039985

C -1.72495866671259 0.17376987497770 3.65275035581114

C -0.32476935740637 0.33090760032589 4.02608725073348

C -2.75478341991024 1.24700983160057 3.72680005714273

C 0.28116896243578 -0.97204011971904 3.98924654888045

C -0.69602176470731 -1.93072599601966 3.53320855393516

C -0.50534409297366 -3.40549006062845 3.43015929468959

C -3.25483027233841 -1.82899368880438 2.98179984365214

C 0.30494764159611 1.59207957983369 4.51038400990305

C 1.68009370769675 -1.28409161965096 4.38278746905557

H -0.11979687943234 2.46496476062542 4.00732924986505

H 1.38387714571186 1.58854368131863 4.34058886928221

H 0.13014857069442 1.70790713072924 5.58764552923711

H	-3.65962147753220	0.98655081562878	3.17421578805523
H	-2.37684545610108	2.20423644436127	3.35552314946806
H	-3.03473437509479	1.39582865564010	4.77746818081159
H	-3.78417923775214	-2.12084305041119	3.89761890675544
H	-3.11947288088174	-2.73130409497440	2.37982124021271
H	-3.89238335876233	-1.13228540425740	2.43159650225906
H	-1.15277121102223	-3.83713415163112	2.66268147537099
H	-0.75010704455758	-3.88503236707605	4.38650002000600
H	0.52922515439598	-3.65354458404591	3.18226266082672
H	2.06738230313341	-2.15074541738559	3.84489413900067
H	1.69335759348124	-1.51147525720902	5.45665635961253
H	2.34674732197600	-0.43821907316974	4.20519720765364
C	-1.11195467831848	0.23275279280622	-1.91977641381202
C	0.22731563327857	0.82636569829131	-1.47220240117898
C	0.09704998539721	1.82951135018691	-0.32791723537687
H	0.70716982612375	1.33709843597593	-2.31424310761737
H	0.90120460878190	0.02333598229407	-1.16700669527058
N	-0.83583163151744	1.36089971194320	0.74970308592034
H	-0.30031949876010	2.78452131090953	-0.70240299438854
H	1.07305664663488	2.01459147771821	0.12984773561141
C	-1.97073909409324	-0.16195331614183	-0.72101561895538
H	-0.94284943467481	-0.63166109555678	-2.57277833041299
H	-1.68831428863305	0.96490304161388	-2.49852909157324
C	-2.17085121384510	1.06201116250066	0.15863795955984
N	-1.29027473646465	-1.21474912637544	0.12484486907394
H	-2.93921476607844	-0.54780256620684	-1.05732930373355
H	-2.88125395030222	0.87038009662009	0.96656554810324

H	-2.53362852521125	1.90772081794737	-0.44230310064578
H	-0.93452260762824	2.13007837286453	1.40818087399689
H	-1.95614981521819	-1.94667475948176	0.35508582194382
H	-0.54838702715346	-1.66559289479481	-0.40932678681652

Charge: 1

Multiplicity: 1

Number of atoms: 49

R_C,S_N,S_{Ir} - [(η^5- C_5Me_5)Ir(L3)Cl]$^+$

Ir	-0.58091995675554	-0.68026450061606	1.81641148449770
Cl	0.97641574280587	-2.29843829657528	0.79822929719210
C	-2.00440560001121	-1.43319623092498	3.31784830629094
C	-2.02567091890830	0.02293484892706	3.32332465465258
C	-0.69550909412563	0.47197957323054	3.65705784889354
C	-3.25656745243128	0.86177778106535	3.28120753121261
C	0.14661673590138	-0.68437331448970	3.85961097270558
C	-0.68627068757267	-1.85889800986775	3.66872841699970
C	-0.25058103703774	-3.26784833887819	3.85975725625727
C	-3.18835940004584	-2.32310286080873	3.14896798230375
C	-0.27370763545470	1.89018152771597	3.82386806332517
C	1.56568440043425	-0.68505343842643	4.31461348703048
H	-0.84596021420521	2.56129550597568	3.17810574417265
H	0.79029419059379	2.01946426964049	3.60935727458717
H	-0.44324324616027	2.19770373097588	4.86330905656744
H	-3.95208275117716	0.55007580760689	2.49776214722693
H	-3.02846659899197	1.91994679292253	3.14182899387062

H	-3.77940503163508	0.75643791366501	4.24086376143134
H	-3.59125907476726	-2.57868961609676	4.13739762689619
H	-2.92352975268565	-3.25499131586889	2.64368916576883
H	-3.98765732558706	-1.83640768339789	2.58631987874966
H	-0.81805128134677	-3.95364730757646	3.22706666795664
H	-0.42260497032842	-3.54769626825046	4.90725682151528
H	0.81141067821152	-3.39320531589120	3.64244642151669
H	2.10784970685830	-1.54427811453257	3.91259359870391
H	1.60942129240410	-0.73843083487486	5.40975059425076
H	2.08501723540837	0.22344740236191	4.00028208904991
H	-3.53344140988148	-1.07536811427067	-0.96449262984460
H	-3.49196949961942	-0.69563879158421	0.75690592425321
C	-3.04096986735015	-0.45100489427388	-0.20651527610640
H	-4.31127981201991	1.23389236420113	-0.24676166660686
C	-3.28119549684954	1.01844471807489	-0.55473354756273
H	-1.58997765720733	-1.87651805551357	-0.24984331779667
N	-1.60430279308535	-0.86615042511291	-0.12168104296425
H	-3.26174942537525	1.14834126363966	-1.64425929964781
C	-2.33170483169742	2.04519371344855	0.07302717793830
H	-2.11497242721303	1.78425534831665	1.11293463467426
C	-0.74799372459081	-0.29310883936404	-1.20573656093131
H	-1.33381474726588	-0.13168615036722	-2.11819012053534
H	-2.83658333430474	3.01798342761910	0.09372048077572
H	0.03051954884895	-1.02798330713711	-1.42501930597855
C	-1.02841924416422	2.21491814776265	-0.70854856985104
C	-0.09997514734654	1.00544379144166	-0.74427681944095
H	-1.26975901776011	2.46356770265045	-1.75024115203730

N 0.50122152464689 0.74373294525269 0.60818772811452

H 0.63618306304441 1.62670862669824 1.09635365740424

H -0.46428766373460 3.06966096897661 -0.31096179220417

H 0.71830190217443 1.23394976368909 -1.44054216640775

H 1.42805210736144 0.33757208884113 0.48490852112979

Charge: 1

Multiplicity: 1

Number of atoms: 49

R_C,S_N,R_{Ir} - [(η^5- C_5Me_5)Ir(L3)Cl]$^+$

Ir -0.30651317280291 -0.35467981921570 2.04160754021388

Cl 2.03068761823598 -0.16955679070348 1.34615720577288

C -1.95040516364357 -1.07492642802085 3.25467093497326

C -1.63279279809871 0.27789567985092 3.64283054486460

C -0.26452316344204 0.31971448361328 4.11007025241533

C -2.57248236020622 1.43211912937689 3.60626137823362

C 0.24689849535987 -1.03949458853193 4.05673178762897

C -0.77041706609589 -1.89136232365694 3.52095077491109

C -0.69148694106019 -3.36987323487532 3.35556552273473

C -3.27838793903325 -1.59193921766753 2.81940127871326

C 0.44233505946491 1.49453137553662 4.69494509810327

C 1.59044773703713 -1.47093918657348 4.52664982115924

H 0.06669937662089 2.43273947076190 4.27973717363996

H 1.51637963243631 1.44325502782308 4.50052917783858

H 0.29605821040457 1.52153299913047 5.78225787792641

H -3.30270811769982 1.33746209029628 2.79727530816223

H	-2.04278769457815	2.38277644030513	3.50095848021094
H	-3.12971230211824	1.46904998236912	4.55052456323561
H	-3.80879733876554	-2.01919971350015	3.67992068301353
H	-3.17306005438796	-2.38049082384207	2.06881890235051
H	-3.90127682467186	-0.79815010866841	2.40107510775336
H	-1.31602688809648	-3.71496028186296	2.52742923895862
H	-1.05421900000613	-3.85849610757150	4.26880359094731
H	0.33434826585584	-3.70049384800384	3.17915052883952
H	1.93822798744876	-2.35889146710568	3.99498503879039
H	1.52865591114024	-1.71331518978629	5.59538357260968
H	2.33099547024609	-0.67945233776711	4.39920714407314
H	-0.36047755604890	3.16063441538735	0.07767340629983
H	0.96600903589795	2.04925989307456	0.46072509809108
C	0.01053084867246	2.13759293448522	-0.06077882313842
H	1.01485619491489	2.59418467969289	-1.83883254746157
C	0.21650388355105	1.89804681107740	-1.55265836401221
H	-1.48910206793654	1.81853709287539	1.26822211506374
N	-0.96048959042055	1.21520462632332	0.64521084023436
H	-0.66888375541010	2.21109840457561	-2.12135003640357
C	0.61898868415735	0.47932219413774	-1.96875137172800
H	1.35776460334740	0.08379246288423	-1.26136121349018
C	-1.96944729437802	0.54558765326057	-0.22619369381321
H	-2.34851894983232	1.22488030277346	-0.99934301345993
H	1.12107083050620	0.52955410866656	-2.94214181126063
H	-2.80834389368608	0.27838432567310	0.42474591853527
C	-0.56683131836179	-0.47568958300395	-2.11749211284124
C	-1.39370202459698	-0.71967882274075	-0.85762697970445

H	-1.25242157709313	-0.07590633987513	-2.87643804297200
N	-0.59530118471145	-1.45435817623471	0.19368552258258
H	0.32789325736813	-1.68373122254792	-0.17318434830985
H	-0.21841070153562	-1.44649857574244	-2.49438734781246
H	-2.24181384236303	-1.35788799810037	-1.13400138869665
H	-1.04477052158445	-2.34174439835260	0.40093966622344

<u>Cartesian coordinates for the diastereomers for the Rh complexes</u>

Charge: 1

Multiplicity: 1

Number of atoms: 43

S_C,R_N,R_{Rh} - [(η^5-C_5Me_5)Rh(L1)Cl]$^+$

Rh	-0.56424508347230	-0.62541150110204	1.90149846830991
Cl	1.21211479694881	-1.92155479251254	0.82345986858357
C	-1.92607245476419	-1.48037894251841	3.36718454878647
C	-1.91688901534859	-0.03463099158465	3.50226060499753
C	-0.57280389338603	0.35154635302769	3.86529544588140
C	-3.12734794923934	0.83340953008206	3.49996802536904
C	0.24292514518328	-0.82216259739886	3.90676935642114
C	-0.60976410663124	-1.96688444551862	3.61781691309709
C	-0.18603449968719	-3.39083410388003	3.66232281697767
C	-3.12514635000610	-2.31048584772475	3.07247555951229
C	-0.10016912069093	1.72415517956838	4.19720565852898
C	1.67555694324797	-0.88500828066684	4.30206637798461
H	-0.83811689953711	2.48781958553953	3.94738439260807
H	0.84074246083818	1.95942944660629	3.69096575404026
H	0.08605169901930	1.78158444644131	5.27701675082835
H	-3.88042883533020	0.46975279373014	2.79680432877726
H	-2.89444108679735	1.87117608321309	3.25216743852574
H	-3.57885648929095	0.82735098025477	4.50081941344865
H	-3.61943383216913	-2.57740818657813	4.01553416568388
H	-2.85633068133381	-3.23763082791236	2.56108513903106
H	-3.85290250141038	-1.76895785146187	2.46286061601664
H	-0.79973302039470	-4.01496341114311	3.00883282543905
H	-0.29470743833761	-3.76489427735952	4.68886433397893
H	0.85893448052820	-3.50277200802922	3.36739476943505
H	2.19959167057062	-1.67643989425365	3.76127356474043
H	1.75158357537321	-1.09957315205526	5.37609731748801

H	2.18341797113743	0.06318264541689	4.11038001663408
C	-2.25804853333729	1.48629717461414	-0.06281067855028
C	-1.08896764377849	2.11590184637102	0.72986600096325
N	-0.02454753837782	1.07084256694800	0.64673413583583
C	-1.62705942153127	0.26625634040024	-0.76239234629995
C	-0.14854351210197	0.61927704662110	-0.76877431432357
N	-1.75643386953645	-0.93257880193953	0.11558217029497
H	-2.04032837468899	0.07338924376572	-1.75773975307984
H	0.51626363926688	-0.21879430915927	-0.97991198515843
H	0.05370416417194	1.44977807696782	-1.45888802288740
H	0.89880161811124	1.43980449230583	0.86097156305929
H	-2.73567131396255	-1.11576411535346	0.32198125158946
H	-1.38682983533881	-1.75565783559349	-0.35872731354690
H	-2.64544495627057	2.18742639672527	-0.80564569770460
H	-3.08871962776416	1.18964860610455	0.58401447243711
H	-1.32626382421708	2.34620172870203	1.76727392342742
H	-0.73382645566432	3.03242561033980	0.24049212281803

Charge: 1

Multiplicity: 1

Number of atoms: 43

S_C,R_N,S_{Rh} - [(η^5- C_5Me_5)Rh(L1)Cl]$^+$

Rh	-0.42332948610642	-0.47244286156733	1.99442560039292
Cl	1.74523269488202	-1.09587693594065	1.06290551529457
C	-1.91917637404489	-1.30801262801887	3.32841276533351
C	-1.77286750040071	0.09942165675820	3.58756912425197

C	-0.40275510903115	0.32396497244259	4.02231242175530
C	-2.85118044659214	1.12638473302522	3.58800196694927
C	0.27258936678132	-0.93186931382820	4.02537755989275
C	-0.65039952404560	-1.95014585240280	3.55677684588880
C	-0.36204689222526	-3.40925813941246	3.47843443782271
C	-3.17412419702309	-1.98701217511597	2.90914258905427
C	0.15314694128702	1.63161085437551	4.46462597961441
C	1.66473207049202	-1.17886081103510	4.47962276717825
H	-0.28510122748139	2.46298231339288	3.90590177119275
H	1.23872004412849	1.66566464782674	4.35060409231245
H	-0.08142627723039	1.78763362069610	5.52562206955047
H	-3.71352666619162	0.81264955039430	2.99590479358600
H	-2.49402887020746	2.08717100766308	3.20580324020076
H	-3.19435202351934	1.29091166563209	4.61763869644471
H	-3.74470689854913	-2.26911630625746	3.80312987552171
H	-2.96931310548971	-2.90150705527925	2.34683992660541
H	-3.80940081819786	-1.33224576225790	2.30618533260178
H	-1.01847533409989	-3.90977333134149	2.76285253396805
H	-0.51436663578790	-3.87500500252787	4.46071403878007
H	0.67324721672182	-3.58886782101154	3.17861317606697
H	2.12374366401771	-2.00397927722799	3.93252766576784
H	1.64352078517725	-1.44375279071646	5.54513666756425
H	2.29120688873598	-0.29242539200268	4.36254178997359
C	-0.24639584643175	0.27490002745157	-1.40473823952466
C	-0.72871320644476	-1.15081872823516	-1.05606051621196
N	-1.54073422027992	-0.94151822914943	0.18092355709227
C	-1.03112909804070	1.19230112824665	-0.44720100944782

C	-2.24578832891935	0.34074313228915	-0.10955972973882
N	-0.28247863382585	1.36621618259755	0.83320279546754
H	-1.28165585778562	2.16205314649221	-0.88903349111445
H	-2.81605114063645	0.69053160617461	0.75468251614717
H	-2.91103696088956	0.23794149613875	-0.97851237877012
H	-2.18650166453252	-1.71158098012596	0.33591724958800
H	0.69255526887483	1.59580987915763	0.65069973104294
H	-0.67913282986236	2.13938885231059	1.36330324607579
H	-0.49387647215624	0.52786122112342	-2.43848926302856
H	0.83291207603029	0.38461069312802	-1.27312158589352
H	0.08238558267185	-1.85277312155740	-0.86372102107095
H	-1.38300095377146	-1.54904987230483	-1.84229510417857

Charge: 1

Multiplicity: 1

Number of atoms: 46

R_C,S_N,S_{Rh} - [(η^5- C_5Me_5)Rh(L2)Cl]$^+$

Rh	-0.40397078689019	-0.39976460333590	1.94677148257804
Cl	1.85200133024487	-0.91171190620248	1.15361787408772
C	-1.97963582839760	-1.18969314112866	3.28626816624362
C	-1.74299470764005	0.19216593476271	3.58095827485366
C	-0.35408762891420	0.32961559814967	3.98274627970381
C	-2.75159420816027	1.28493921699221	3.66132790898268
C	0.23665761459027	-0.97205151289826	3.96531476183739
C	-0.74158488315050	-1.91186787556021	3.46667342160514
C	-0.55733256437138	-3.38528419770582	3.34470162049779

C	-3.29629013480748	-1.81417948143924	2.99214259587966
C	0.28420379249799	1.59181606368403	4.44878641632394
C	1.60562315353125	-1.31066219547641	4.42713942581338
H	-0.12302396819733	2.45872870306207	3.92119307918560
H	1.36634762872520	1.57035343329234	4.30087417373246
H	0.09141930603824	1.73306461220602	5.52015062084978
H	-3.70539934267012	1.00362112898891	3.21189161749772
H	-2.39497255419182	2.20485438944548	3.18775770826100
H	-2.93437902155214	1.51649982018124	4.71868779280114
H	-3.75702956024664	-2.09934793101822	3.94762169791575
H	-3.19161150037359	-2.72444345976512	2.39788043406992
H	-3.98358237413242	-1.13180683100236	2.48765898535041
H	-1.20811332151081	-3.80398457078157	2.57305461483287
H	-0.80141222108052	-3.87750281895304	4.29514466329446
H	0.47656691224027	-3.63330136765091	3.09323660566758
H	2.00912790193667	-2.17788275034735	3.90276738232725
H	1.55177939714243	-1.55144036595733	5.49765446699672
H	2.29484560949868	-0.47350851510815	4.30278425005552
C	-2.29673382921165	1.51334546905330	-0.66354348468810
C	-3.08232685703865	0.41325406556600	0.05882103856653
C	-2.55242249309499	-0.97847316312696	-0.26131354649286
H	-4.14140750541653	0.46604268844190	-0.21633052950161
H	-3.02412462131668	0.57479816832973	1.13824897052346
N	-1.07907195748387	-1.01845696784512	-0.04844171009520
H	-2.76051674090389	-1.23723089676290	-1.31074416468133
H	-3.02076664770310	-1.73720183322529	0.36945925813868
C	-0.78315523780188	1.30939216548011	-0.58328578107664

H	-2.57049131810617	2.49901200332563	-0.26768606214709
H	-2.55593806495238	1.51496360617091	-1.72961203362638
C	-0.43143869214466	-0.09920494968897	-1.02659985589152
N	-0.27183324186770	1.45275024003946	0.81755456964145
H	-0.28147920413963	2.04139754649422	-1.22963630420079
H	0.64650749653099	-0.26640058109558	-1.01397947422523
H	-0.81409329480212	-0.28878374820168	-2.03886690265106
H	-0.73923146158357	-1.96490531317603	-0.20496009322754
H	0.72205498243610	1.67352599847644	0.79010021208924
H	-0.73243935155833	2.23108012531120	1.28472957230079

Charge: 1

Multiplicity: 1

Number of atoms: 46

R_C,S_N,R_{Rh} - [(η^5- C_5Me_5)Rh(L2)Cl]$^+$

Rh	-0.42269434051569	-0.41214541631542	1.97907308642595
Cl	1.82249266878521	-0.78966766288182	1.12844604252874
C	-1.94361021937328	-1.18438879797465	3.32789854783582
C	-1.70262395304797	0.19194452300682	3.63988530814224
C	-0.30654327650930	0.33096458077750	4.01353437908059
C	-2.71624856823223	1.27920721726615	3.70478912448450
C	0.28091855528536	-0.97818804085282	3.98689555027361
C	-0.70252387625221	-1.91512947687344	3.51394880735104
C	-0.52558232025338	-3.38815946226017	3.38093881431305
C	-3.25386163983418	-1.78557621778428	2.95961497873340
C	0.34276335896332	1.58548839373178	4.48637639524195

C	1.66300745213616	-1.31229373873027	4.41158254615624
H	-0.07834495567013	2.46215502024245	3.98682830657438
H	1.41986113244966	1.56807204086331	4.30540366013748
H	0.18230325456472	1.70658131136091	5.56558297567760
H	-3.63293779667807	1.02109675608379	3.17134635297030
H	-2.33227830804467	2.22723116489207	3.31629811810705
H	-2.97846944268645	1.44978672699604	4.75705007402705
H	-3.78838028631121	-2.06831213151929	3.87572774105668
H	-3.12910533532689	-2.69269076412753	2.36284613871063
H	-3.88493044974997	-1.08378695298478	2.40852472978389
H	-1.16377400355186	-3.79629463840805	2.59298461074883
H	-0.79455205241142	-3.88604132822155	4.32157681677676
H	0.51118843500446	-3.64229728219405	3.14917060377272
H	2.05137700865423	-2.18341867569717	3.88202627643077
H	1.64306222178751	-1.54399839598833	5.48513354651400
H	2.34668166295392	-0.47562285890242	4.25792927003630
C	-1.05305337643194	0.20523141495118	-1.89670256540122
C	0.27551947547819	0.80872062637988	-1.43246466180630
C	0.11310088332246	1.83087947456925	-0.31006651728109
H	0.77317969826205	1.30460826331916	-2.27311351611999
H	0.94382362099233	0.01369454046476	-1.09448522260275
N	-0.82193272888914	1.36199771883443	0.76014260356229
H	-0.29432557586589	2.77212825253363	-0.71029402892815
H	1.08001638452745	2.04234459696766	0.15618146976193
C	-1.92567112810911	-0.19276691491799	-0.70843719498750
H	-0.87023138474200	-0.66037171988655	-2.54457757731418
H	-1.62490557896907	0.93282332946693	-2.48585844089439

C	-2.14574277741854	1.03179975649452	0.16738677727468
N	-1.25419372328542	-1.24023589531599	0.14240998812763
H	-2.88939580364890	-0.57848953593622	-1.06022038843529
H	-2.85658168749244	0.82881633119250	0.97242977643536
H	-2.52875010861416	1.86714719198652	-0.43690985538733
H	-0.93396783209898	2.13034952045932	1.41753565263199
H	-1.92171718372816	-1.96736341746531	0.38289848145090
H	-0.50942609942431	-1.69808942760245	-0.38026758197828

Charge: 1

Multiplicity: 1

Number of atoms: 49

R_C,S_N,S_{Rh} - [(η^5- C_5Me_5)Rh(L3)Cl]$^+$

Rh	-0.57389664759024	-0.70087054100672	1.80443764428787
Cl	0.95602991735716	-2.37395058742299	0.86156442345114
C	-1.99386842895309	-1.43617886532996	3.31271789817517
C	-2.02477512881993	0.01448990952469	3.30941251040208
C	-0.70312883397679	0.47756397768016	3.63218489067911
C	-3.26067706596714	0.84262908112395	3.24509280704528
C	0.14667003342173	-0.66809480499020	3.83647840102996
C	-0.67695866138827	-1.84891519255554	3.67227223099580
C	-0.24155992841559	-3.25002361362896	3.89969994470511
C	-3.16854357011379	-2.33563251362472	3.13851720285598
C	-0.29094575689277	1.89877655792129	3.78589913846276
C	1.57174905296616	-0.65223359704472	4.26890487037898
H	-0.86778353812991	2.56368314321129	3.13798569385703

H	0.77266739783682	2.03409062459650	3.57376840648052
H	-0.46278912237057	2.21191362323300	4.82374345235462
H	-3.93310279780487	0.53660410646248	2.43933667853115
H	-3.03970247506020	1.90516151800995	3.13103786090916
H	-3.80934349837144	0.71421879022760	4.18760708735805
H	-3.56802069835891	-2.60554666859313	4.12485935552723
H	-2.89385369301745	-3.26125618594640	2.62642956489642
H	-3.97333555798898	-1.85216650809861	2.58099072471711
H	-0.78330564750661	-3.94977886973363	3.25969886742146
H	-0.45454546473879	-3.51218210411993	4.94490750347040
H	0.82837245507686	-3.37451009521903	3.72752178667173
H	2.11272026664185	-1.51502899318972	3.87306607631937
H	1.63227255527892	-0.68802810696432	5.36426862196166
H	2.08037682906778	0.25501628716723	3.93404255144678
H	-3.51286182205532	-1.06860416190338	-1.00570864735898
H	-3.50321896423694	-0.70965789759471	0.71902091728186
C	-3.02706072470618	-0.45781976326617	-0.23154696438955
H	-4.27320043161625	1.24907282568012	-0.26702083195441
C	-3.24434313024890	1.01821270598660	-0.56768519729936
H	-1.58822045234406	-1.89610538710891	-0.22729564370560
N	-1.59988924034546	-0.88324892858591	-0.12092129538342
H	-3.21204983146707	1.15744645405723	-1.65571531465717
C	-2.28140346391762	2.02182807642810	0.07683265195352
H	-2.07231391576221	1.74418423606693	1.11410766996353
C	-0.71515860675377	-0.32958041581993	-1.18917229255220
H	-1.27782078812316	-0.16573078534493	-2.11672338025296
H	-2.77141902544168	3.00181934640783	0.11081780602791

H 0.05787421935800 -1.07653244685150 -1.38730416799444

C -0.97261969795199 2.18170517894682 -0.69770445790412

C -0.05442230650147 0.96389493063838 -0.72769254156113

H -1.20694762175160 2.43467525051933 -1.73991804584791

N 0.53830868596745 0.70492829937841 0.62472219839777

H 1.45163688497175 0.26681217706394 0.51505217437065

H -0.40341943855564 3.03085196245975 -0.29520324137764

H 0.76509050894537 1.18372293600714 -1.42671651283702

H 0.69278717035508 1.58723503514531 1.10828892268841

Charge: 1

Multiplicity: 1

Number of atoms: 49

R_C,S_N,R_{Rh} - [(η^5- C_5Me_5)Rh(L3)Cl]$^+$

Rh -0.27770001867768 -0.34220499556430 2.03871278610314

Cl 2.07483549115067 -0.16034074168919 1.42877370979340

C -1.94002836936875 -1.03988271643654 3.23276974790735

C -1.61179347616977 0.30069347933154 3.63720630732662

C -0.25013438299611 0.31982705780908 4.10515075194665

C -2.53487027846469 1.46631125512529 3.60179448538588

C 0.24279490563959 -1.04363204679405 4.05414129860423

C -0.77879663725128 -1.87322206415152 3.50319237586506

C -0.71620777346863 -3.34909175005147 3.31953564912602

C -3.26706943922921 -1.53605702157145 2.77335695897674

C 0.47721776404586 1.48541823548538 4.68061874545735

C 1.56801356368383 -1.49928337499479 4.54662748431035

H	0.11087098382190	2.42857434499446	4.26845162100723
H	1.54914596377377	1.41765329802163	4.47996327281663
H	0.33934633377630	1.51555465169894	5.76920736839589
H	-3.26425312203169	1.38662324744747	2.79040255615876
H	-1.99398479867704	2.41186561353946	3.50932230049915
H	-3.09744873681728	1.50199375666163	4.54342862297944
H	-3.79467421335038	-2.00412952168144	3.61441365728927
H	-3.16315760294602	-2.29163279957087	1.98902362156715
H	-3.89339294672148	-0.72546181047238	2.39497468342966
H	-1.35683688073311	-3.68034366259493	2.49797912003958
H	-1.07086513977918	-3.84460993720214	4.23261195202129
H	0.30434291697512	-3.68799428362128	3.12781566952639
H	1.91324932265546	-2.38838147085500	4.01515757385405
H	1.47750560251762	-1.75233934845991	5.61140323515204
H	2.32341706148588	-0.71836257086144	4.44577429026199
H	-0.32869151884068	3.16877375178797	0.09656661164571
H	0.99183988450361	2.05589325686746	0.49590138051709
C	0.03980882619617	2.14322014932349	-0.03378189440215
H	1.05860944199390	2.59314364681640	-1.80668050509342
C	0.26029083205813	1.89641246023052	-1.52211607474513
H	-1.46440991574020	1.82350006668152	1.29005343387310
N	-0.93457971737894	1.22501684060857	0.66342603415216
H	-0.62193613237766	2.20177694571303	-2.10007233018726
C	0.66966014443469	0.47520019191918	-1.92294116320851
H	1.39565932808758	0.08365002953934	-1.19974724927269
C	-1.93443574882708	0.54232844457004	-0.20360387446235
H	-2.32389595101542	1.21329122030183	-0.98005184627642

H	1.18830069446153	0.51852646611001	-2.88797898236034
H	-2.77166391626406	0.26994468557924	0.44779757291961
C	-0.51576352951217	-0.47806707739520	-2.08472118886257
C	-1.35352610092442	-0.72351417765012	-0.83195048863031
H	-1.19288122706099	-0.07571114447083	-2.84995731321933
N	-0.56728721217761	-1.46089295441409	0.22054293078040
H	-1.02002640854016	-2.34590770010869	0.43105720783500
H	-0.16530045785017	-1.44907489019418	-2.45921977732232
H	-2.20217973721383	-1.35672811872688	-1.11983643046373
H	0.36025232914417	-1.69112691663076	-0.13260589901799

Cartesian coordinates for the diastereomers for the Ru complexes

Charge: 1

Multiplicity: 1

Number of atoms: 42

S_C,R_N,R_{Ru} - [(η^6-cymene)Ru(L1)Cl]$^+$

Ru	6.69087280044121	4.82991553704497	11.36596309919183
Cl	6.68510618941340	6.88928118477191	12.67174167683419
C	7.40476554653503	3.42552280371435	12.92700708041695
H	8.18815647607731	3.61202351188000	13.65123272322167
C	7.71410852914436	2.88856567544083	11.64801400890688
C	6.07922800707887	3.83728326296529	13.22118411740174
H	5.89340697683486	4.36114472928376	14.15409753539332
C	5.34486841801587	3.15813979550720	11.01798130461982
H	4.57175753191390	3.12069482817837	10.25492407074523
C	9.10985406347846	2.45166152450429	11.27062810224142
H	9.25395367507794	2.70889251835683	10.21091063090636
C	6.66030070653839	2.73699448046880	10.69568078728069
H	6.88309121962627	2.36062686965724	9.70162034729632
C	5.01475065095762	3.70066832894446	12.29382350838596
C	9.17476192601610	0.91755094320564	11.38506305991463
H	8.99844836296350	0.60693650973691	12.42093336264448
H	10.16715577228448	0.56703692645519	11.08475862840788
H	8.42894439641930	0.43429961476416	10.74651072838081
C	3.62519551927704	4.15382144918268	12.61926450260972
H	3.64342295444244	4.98175090367219	13.33277561049437
H	3.05947362771243	3.32966586097841	13.07012490830858

H	3.09567428571452	4.47217281758355	11.71709761932303
C	10.21987361620568	3.09761318114776	12.10019718157231
H	10.13954769680046	4.18945598592883	12.13400225959426
H	11.19162353513880	2.84074530948351	11.66865332306094
H	10.20847333660945	2.72648149194325	13.13055562515402
C	7.40976691558768	5.26286958903369	8.10617475183545
C	5.90414949051800	5.18221475030649	8.44669213880073
N	5.80887159184953	5.95672036152873	9.72762024351792
C	7.95165281128795	6.33542835547245	9.07021328706704
C	6.70134360315460	7.11683437870098	9.43143830204760
N	8.39538048605033	5.68242998474849	10.33967430803690
H	8.75446864582895	6.94413633645404	8.64331734622289
H	6.80735147119486	7.77319389604728	10.29461918731953
H	6.32430109311820	7.68032541457515	8.56778572931843
H	4.85401574555209	6.24546450206731	9.92856533377655
H	9.12476179184888	5.00315786743971	10.13554113224314
H	8.80390294164863	6.38165956871037	10.96010228769957
H	7.55908112420611	5.57708097386787	7.07063279563985
H	7.92008183346449	4.30482611166336	8.24550901948408
H	5.52889931047213	4.16733695463344	8.56648159945542
H	5.30242532349920	5.69052490994986	7.68259673522646

Charge: 1

Multiplicity: 1

Number of atoms: 42

S_C,R_N,S_{Ru} - [(η^6-*p*-cymene)Ru(L1)Cl]$^+$

Ru	6.73348843843182	4.72152462979752	11.33410989512358
Cl	7.50091176890554	6.61504844456464	12.66215509269052
C	7.31980931950434	3.34248547768857	12.94161272037053
H	8.05731111130109	3.56154306105082	13.70548343263377
C	7.72405786921682	2.77823047665793	11.71530338897752
C	5.96659372960428	3.75279532567391	13.16214341020120
H	5.71377862069864	4.26556462074243	14.08452659254852
C	5.39558491985095	3.05944584313729	10.89618774364570
H	4.68519611258731	3.01520682130119	10.07492113150912
C	9.14519447889421	2.34372391492032	11.44428860769132
H	9.28768547244354	2.37839278550498	10.35473550256979
C	6.72770950255793	2.64254908730753	10.68799959065002
H	7.02641990470983	2.28712302859692	9.70463358816476
C	4.98950278724822	3.62162539620474	12.15549866408456
C	9.30442982221088	0.88091431985780	11.88956707388086
H	9.16941550097277	0.79791274410886	12.97399576699500
H	10.30682437808658	0.52002511642927	11.63821825980187
H	8.57061540797699	0.23207617237906	11.40021546742694
C	3.58323741590874	4.08634589455443	12.36976514810664
H	3.53819859158370	4.86956601315924	13.13018604686019
H	2.97522630919417	3.24034845057627	12.71284571618736
H	3.13688357191550	4.45512730983646	11.44188497064454
C	10.19757919212435	3.24144736296466	12.09571566705852
H	10.04339203055974	4.29347297713480	11.83088583288833
H	11.19395199044598	2.94106270227001	11.75759909773893
H	10.18146808252587	3.15493174894644	13.18726302160422
C	7.44548634621923	7.55403588649219	9.38624902484675

C	8.62692279213410	6.59354227186972	9.64566561416074
N	7.97843403429836	5.24438849258554	9.62372067838469
C	6.27187322747765	6.62518187028285	9.02593944151533
C	6.98861936998785	5.38801285743097	8.51213051717954
N	5.55300749035152	6.18588918920011	10.26365959790246
H	5.57283477746674	7.06798648246946	8.31003814728233
H	6.35574958495600	4.50195484736307	8.42007437198981
H	7.49544342206105	5.58867257989291	7.55828083217528
H	8.66130299422057	4.51651008604397	9.42732903590742
H	5.36673286806041	6.98656451108495	10.86596100597711
H	4.65132914407362	5.79126935002256	10.00453794538513
H	7.65815160672053	8.21315818843151	8.54104067415803
H	7.22032465692697	8.17428631476920	10.25691888492248
H	9.12787182754856	6.75898824061970	10.59887344292253
H	9.36060952803665	6.63968910607491	8.83115935523545

Charge: 1

Multiplicity: 1

Number of atoms: 45

R_C,S_N,S_{Ru} - [(η^6-*p*-cymene)Ru(L2)Cl]$^+$

Ru	6.75453418937274	4.75974262758555	11.31152511543602
Cl	7.59970103881249	6.62294613598264	12.64760650194960
C	7.34279590413674	3.40708928790998	12.94582467024201
H	8.07234545870776	3.64908278845537	13.71017253292016
C	7.76025468971427	2.81746357905851	11.73724785738619
C	5.98666710397707	3.81577266718152	13.14658663808929

H	5.72544335556250	4.35189308351458	14.05322352607527
C	5.43357496774898	3.05660808324546	10.89981865635773
H	4.72109172958593	2.97067609034796	10.08433113670139
C	9.17940899042461	2.36462078714487	11.49033132275424
H	9.37136501761988	2.48581526028953	10.41517696085405
C	6.77088201609833	2.65155568578884	10.70917565768590
H	7.07128169827209	2.24667638518587	9.74702964515339
C	5.01761841624648	3.65160045413908	12.13764042637702
C	9.27766421889093	0.86551413599509	11.81851484686509
H	9.08339833910703	0.69700908210915	12.88387521443405
H	10.28267543067072	0.49739178125522	11.58895318527859
H	8.55536192343628	0.28048795387611	11.23977841140297
C	3.60768924380167	4.11486903992086	12.32970261834949
H	3.55721607935970	4.93921110424540	13.04532942265440
H	3.00772025139512	3.28491427864168	12.72230621734749
H	3.15689704753653	4.42666790234892	11.38331937923200
C	10.22648092933166	3.16833241163327	12.26012610304537
H	10.10708313969877	4.24505697604621	12.09683125921054
H	11.22738069579431	2.87758382995977	11.92742917716238
H	10.16922817990852	2.97494970385048	13.33672653120155
C	5.97592285121809	6.05096547111200	7.84531413221654
C	6.63887062801249	4.66828409232238	7.88282552118920
C	8.05171150801037	4.71099892298121	8.45492514850286
H	6.68616555295234	4.25321884601939	6.87019711502310
H	6.02565416463020	3.98347938979339	8.47368021278704
N	8.07767537389572	5.48574851431398	9.73403583742822
H	8.73663608104156	5.20089715968161	7.74713836904009

H	8.43277278349375	3.70421069487227	8.64359952293314
C	6.21544085653977	6.86245762801978	9.11816028826438
H	4.90101656952681	5.95093867462324	7.65194788292381
H	6.39333583084615	6.64119839879349	7.02026372459691
C	7.69766382858659	6.89687087007189	9.43181955594991
N	5.54727601023100	6.24503318367528	10.31468672385744
H	5.83135216332853	7.88105296900478	8.98106325073897
H	7.91441465101295	7.51988110314224	10.29980694171206
H	8.26699831393646	7.26888165885372	8.56973500761531
H	9.02615307209809	5.47562531284962	10.10486566356506
H	4.62858940030571	5.89696698237420	10.04874855326074
H	5.39237030512188	6.97027901178337	11.01425353422844

Charge: 1

Multiplicity: 1

Number of atoms: 45

R_C,S_N,R_{Ru} – [(η^6-*p*-cymene)Ru(L2)Cl]$^+$

Ru	6.73337439122670	4.71519710598904	11.31630504846678
Cl	7.45539033524879	6.62105114223988	12.64512390848195
C	7.34679768623978	3.36055370518506	12.95001399004710
H	8.08693273161593	3.59214811641552	13.70689307805819
C	7.74416034537319	2.78604807758049	11.72626772385556
C	5.99425719625890	3.77149503689871	13.16535077573308
H	5.74612967282404	4.30521767569767	14.07735084567092
C	5.41111150267965	3.02725126358816	10.92624628013893
H	4.69097392612157	2.96076823348776	10.11410660166706

C	9.16316915429579	2.34839007110360	11.45403206910421
H	9.32529523040803	2.43457183964691	10.37003037188791
C	6.74485088441914	2.62240058905767	10.70534623839568
H	7.03638927556828	2.24438496820648	9.72866235963799
C	5.00496181371662	3.60417899779571	12.17763924966179
C	9.28681363746973	0.85945073645722	11.82061694071569
H	9.12399774051171	0.71878851134697	12.89506329712325
H	10.28965550064850	0.49752018463704	11.57288127041754
H	8.55589079936325	0.25130878669865	11.27783182458626
C	3.59120807614706	4.04133487577541	12.40426284017429
H	3.54173648546063	4.84569768675535	13.14204478281527
H	3.00863846603245	3.19287715908284	12.78344400965518
H	3.11726515287890	4.37293654031341	11.47591339252905
C	10.21982101202570	3.18537184478332	12.17441972708061
H	10.08317022697799	4.25621637835115	11.98713687686000
H	11.21514561855539	2.89744008094012	11.82318842135694
H	10.19247696588916	3.01970707156360	13.25654879758481
C	7.62210323283591	7.38165791812691	8.47303575095196
C	6.90822786403280	8.04568506979312	9.65416018574348
C	5.54850844801087	7.42738009710305	9.97089269123298
H	6.75250236410780	9.10949432574422	9.44383614948052
H	7.54048749732159	7.99105617339135	10.54233406195052
N	5.56521252235501	5.92693338352704	9.93359319640340
H	4.80097665889638	7.75443602857112	9.23285967433003
H	5.21137747186391	7.73463301654546	10.96498831820819
C	7.52189059281427	5.86064658845632	8.54946690080327
H	8.67210395214853	7.69591407931751	8.44122668834886

H	7.16721296725699	7.68396216794335	7.52188309638574
C	6.05564637862969	5.46893722338555	8.60202985808516
N	8.16593824405671	5.32273810403502	9.80785812623898
H	8.00655643042396	5.40388250445041	7.67956757135451
H	5.91689850139380	4.38779477788562	8.52271347324037
H	5.49802871995078	5.95733281862744	7.79035440834804
H	4.59902325976567	5.62492426398591	10.03803603756776
H	8.78798304611146	6.02370017875529	10.21012292961893
H	8.75533802006655	4.53147460075733	9.56428016000059

Charge: 1

Multiplicity: 1

Number of atoms: 48

R_C,S_N,S_{Ru} - [(η^6-*p*-cymene)Ru(L3)Cl]$^+$

Ru	6.70605603951296	4.78609384725017	11.30807362515575
Cl	7.57817133276971	6.61639288498105	12.67803699192070
C	7.32277599117189	3.36324820564088	12.87994649971857
H	8.05700754297445	3.56888791911784	13.65093734193487
C	7.73251614182104	2.81483882756104	11.64994246100476
C	5.96816670791154	3.75881046512030	13.10720601635455
H	5.70943123128284	4.25595897269073	14.03650980542217
C	5.40137865358353	3.08668432855726	10.83450045735370
H	4.68217420213062	3.02844456770825	10.02272211994933
C	9.14126804860169	2.32403283691086	11.41364871184162
H	9.30940321212801	2.32117270826648	10.32887703821704
C	6.74108724719135	2.70245803310633	10.61747511818464

H	7.03628220012703	2.34551208873533	9.63447381141246
C	4.99191589614640	3.62359479627334	12.09978015503313
C	9.23092535896807	0.86732213799836	11.89965601313300
H	9.07287942330402	0.81779351503869	12.98305071983921
H	10.22205645502004	0.45960911518730	11.67647652689388
H	8.47982313582239	0.23690582689712	11.41251821304912
C	3.57992105857937	4.06552951839719	12.32460792494032
H	3.53192683459392	4.88269855321504	13.04880953187382
H	3.00166416223125	3.22328632700677	12.72346538054252
H	3.10234589952737	4.37673653845000	11.39137216939754
C	10.21794959316022	3.19034962994267	12.06704984088661
H	10.12092948765393	4.24139956165775	11.77506163404781
H	11.20706019526818	2.83745988640038	11.75968614005393
H	10.17414122075782	3.13239040180355	13.15974384467861
C	8.51232404473730	4.84800146363538	8.63996279163164
N	8.02229630426507	5.66232435978019	9.79928081818774
C	7.48642660110389	6.99852217132643	9.38998263077774
C	5.97934949043647	6.92214975244785	9.21656003731546
H	7.97259367816451	7.34855277656879	8.47204268293403
H	7.72251531671479	7.70360544738865	10.19106972828298
C	7.61944315830935	4.87757212554042	7.40000951693580
H	8.63360430002514	3.82397205226198	8.99682093900791
H	9.51050070314757	5.21593754294133	8.36500512079825
C	6.10849703328263	4.82884407800757	7.64694985306962
H	7.92033408149463	4.01627016259523	6.79205395527024
H	7.84741486421305	5.76473669522655	6.79568645502821
C	5.50043960146264	6.19947011870276	7.95898409715806

H	5.87162229257949	4.10763123826500	8.43364714182580
H	5.62119492665328	4.46060089454563	6.73656773398166
H	4.40577156245891	6.11764751640998	8.00620251396933
H	5.71915265943733	6.86859937337239	7.11645859812949
H	5.59345089609467	7.95010705986629	9.16799903943303
N	5.44118304907659	6.28730543843992	10.46604311774464
H	8.83966111189370	5.85182909162805	10.37746587305890
H	4.49508160782740	5.95403926512360	10.29378565951174
H	5.36886544438157	7.00144988201113	11.19072360310741

Charge: 1

Multiplicity: 1

Number of atoms: 48

R_C,S_N,R_{Ru} - [(η^6-*p*-cymene)Ru(L3)Cl]$^+$

Ru	6.75653804505606	4.71973767674768	11.35683555847977
Cl	7.58319700212251	6.57803487062766	12.69233679551040
C	7.33374387289622	3.31983056576532	12.96081317121287
H	8.05740649361274	3.53079409265595	13.73965034472083
C	7.76065581302228	2.78041006503563	11.73100228699777
C	5.97522970708295	3.72127222095948	13.16230016416846
H	5.70444225312742	4.22214772794234	14.08642098093566
C	5.44340610196697	3.04265349003330	10.88681217297251
H	4.74236844207673	2.99837863815924	10.05666766941315
C	9.18595094753027	2.35151710658804	11.47722631180078
H	9.37055495271044	2.46981473181628	10.39983659884264
C	6.78419225883855	2.65226903302556	10.68262557795244

H	7.09757556319338	2.30888310113110	9.69999590856169
C	5.01031746955799	3.58123840040549	12.14668269628243
C	9.30584235905060	0.85343236248277	11.80479823566771
H	9.12214995164461	0.68285221962567	12.87158023202792
H	10.31394206103991	0.49940499411800	11.56708387914933
H	8.58672442114476	0.25939060144533	11.23140763831523
C	3.59190527672605	4.01422796503272	12.35374582680985
H	3.52723755555213	4.80142066361495	13.10874095650681
H	3.00193996680225	3.15733192568489	12.70105993548353
H	3.13485832661068	4.36673344775723	11.42442545299566
C	10.22541799260449	3.17079457754912	12.24141708168738
H	10.09173770308849	4.24560425558066	12.07619871513428
H	11.22869124307857	2.89145445271652	11.90610500501236
H	10.17384665389892	2.97948112400005	13.31846257701720
C	5.24238601678362	7.44995299052389	10.42935025329678
N	5.54338732829518	6.00906332629694	10.07814841315382
C	6.06957477689177	5.77201919792894	8.70263633490942
C	7.57671445201831	5.99388262558909	8.65343288148719
H	5.55912739549654	6.39917756428623	7.96154683754450
H	5.84899441904115	4.72669690851679	8.46268281650022
C	5.95307866344467	8.51724064599093	9.60374415892599
H	5.49024439762124	7.57755953273560	11.48561374309023
H	4.16007463008034	7.58898779069777	10.31577537974570
C	7.48139056660089	8.42931704771175	9.54214606954107
H	5.66907586983086	9.46745245619921	10.07312690706604
H	5.54473872141371	8.55486340906672	8.58535333250161
C	7.99876690575284	7.45136733362616	8.48582340754096

H	7.87604919739158	8.18279544601173	10.53503808156518
H	7.87882661450215	9.41983479358868	9.29095340678784
H	9.09557513611529	7.49039180805004	8.44748896197133
H	7.64218936410738	7.77323165177160	7.49836287183849
H	7.95967526108118	5.43589434959353	7.79029287986644
N	8.17943823883751	5.37926395081373	9.89522272702686
H	4.64372108276275	5.53812168091414	10.10893221640026
H	8.77505692421085	6.06373212365461	10.36143227016201
H	8.79071160368362	4.61414305593057	9.62372427541862

Calculated energies for all species

E_{el}(*r^2*SCAN-3c) = electronic energy of the optimised geometry at the *r^2*SCAN-3c level of theory

G(*r^2*SCAN-3c) = total Gibbs free energy of the optimised geometry at the *r^2*SCAN-3c level of theory

G_{corr}(*r^2*SCAN-3c) = Gibbs free energy correction at the *r^2*SCAN-3c level of theory (*i.e.* G(*r^2*SCAN-3c) - E_{el}(*r^2*SCAN-3c))

E_{el}(ωB97M-V) = electronic energy from a single-point energy calculation of the *r^2*SCAN-3c optimised geometry at the ωB97M-V level of thery

Final G = E_{el}(ωB97M-V) + G_{corr}(*r^2*SCAN-3c)

Compound	E_{el}(*r^2*SCAN-3c)	G(*r^2*SCAN-3c)	G_{corr}(*r^2*SCAN-3c)	E_{el}(ωB97M-V)	Final G
S_C,R_N,R_{Ir} - [(η^5-C_5Me_5)Ir(**L1**)Cl]$^+$	-1222.45306802	-1222.12479221	0.32827582	-1222.51547629	-1222.18720047
S_C,R_N,S_{Ir} - [(η^5- C_5Me_5)Ir(**L1**)Cl]$^+$	-1222.45155404	-1222.12321923	0.32833481	-1222.51352595	-1222.18519115
R_C,S_N,S_{Ir} - [(η^5- C_5Me_5)Ir(**L2**)Cl]$^+$	-1261.76068297	-1261.40338997	0.35729300	-1261.82975980	-1261.47246680
R_C,S_N,R_{Ir} - [(η^5- C_5Me_5)Ir(**L2**)Cl]$^+$	-1261.75504654	-1261.39856228	0.35648426	-1261.82382420	-1261.46733994
R_C,S_N,S_{Ir} - [(η^5- C_5Me_5)Ir(**L3**)Cl]$^+$	-1301.05641190	-1300.67197261	0.38443928	-1301.13288310	-1300.74844381
R_C,S_N,R_{Ir} - [(η^5- C_5Me_5)Ir(**L3**)Cl]$^+$	-1301.05101857	-1300.66664674	0.38437183	-1301.12723668	-1300.74286485
S_C,R_N,R_{Ru} - [(η^6-cymene)Ru(**L1**)Cl]$^+$	-1212.40896311	-1212.08566044	0.32330267	-1212.47260095	-1212.14929828
S_C,R_N,S_{Ru} - [(η^6-*p*-cymene)Ru(**L1**)Cl]$^+$	-1212.40860648	-1212.08546394	0.32314254	-1212.47192364	-1212.14878111
R_C,S_N,S_{Ru} - [(η^6-*p*-cymene)Ru(**L2**)Cl]$^+$	-1251.71882435	-1251.36689640	0.35192795	-1251.78976356	-1251.43783561

R_C,S_N,R_{Ru} – [(η^6-*p*-cymene)Ru(**L2**)Cl]$^+$	-1251.71171404	-1251.36048218	0.35123186	-1251.78193665	-1251.43070479
R_C,S_N,S_{Ru} - [(η^6-*p*-cymene)Ru(**L3**)Cl]$^+$	-1291.01301726	-1290.63369460	0.37932266	-1291.09142053	-1290.71209787
R_C,S_N,R_{Ru} - [(η^6-*p*-cymene)Ru(**L3**)Cl]$^+$	-1291.00722306	-1290.62872335	0.37849971	-1291.08481713	-1290.70631742
S_C,R_N,R_{Rh} - [(η^5-C_5Me_5)Rh(**L1**)Cl]$^+$	-1228.67644580	-1228.34892547	0.32752033	-1228.72904711	-1228.40152678
S_C,R_N,S_{Rh} - [(η^5- C_5Me_5)Rh(**L1**)Cl]$^+$	-1228.67549118	-1228.34782981	0.32766138	-1228.72778765	-1228.40012627
R_C,S_N,S_{Rh} - [(η^5- C_5Me_5)Rh(**L2**)Cl]$^+$	-1267.98407667	-1267.62719250	0.35688417	-1268.04313340	-1267.68624923
R_C,S_N,R_{Rh} - [(η^5- C_5Me_5)Rh(**L2**)Cl]$^+$	-1267.97920425	-1267.62356129	0.35564296	-1268.03828184	-1267.68263889
R_C,S_N,S_{Rh} - [(η^5- C_5Me_5)Rh(**L3**)Cl]$^+$	-1307.27900739	-1306.89494678	0.38406061	-1307.34545108	-1306.96139047
R_C,S_N,R_{Rh} - [(η^5- C_5Me_5)Rh(**L3**)Cl]$^+$	-1307.27476341	-1306.89045924	0.38430417	-1307.34124490	-1306.95694073

Diastereomers energies: Calculated in Orca 5.0.4 at the ωB97M-V/def2-QZVPPD/CPCM(DCM) // r^2SCAN-3c/CPCM(DCM)/298.15 K

Compound	E_{el}(r^2SCAN-3c)	G(r^2SCAN-3c)	G_{corr}(r^2SCAN-3c)	E_{el}(ωB97M-V)	Final G
Ru L2 / IPA TS	-984.68855707	-984.28065056	0.40790651	-984.76562330	-984.35771679
Ru L2 / pro-R TS	-1176.39091862	-1175.93398301	0.45693562	-1176.52279098	-1176.06585536
Ru L2 / pro-S TS	-1176.39083551	-1175.93413822	0.45669729	-1176.52255831	-1176.06586102
Ru L2 / pre-catalyst (cycle starting point)	-1251.72535202	-1251.38574154	0.33961049	-1251.79638306	-1251.45677258
Ru L2 / pre-catalyst ring N-deprotonated	-1251.22940897	-1250.90615336	0.32325561	-1251.29611299	-1250.97285738

Ru L2 / pre-catalyst NH_2-deprotonated	-1251.22915156	-1250.90602513	0.32312644	-1251.29706832	-1250.97394188
Ru L2 / metal hydride	-791.57317763	-791.24212507	0.33105256	-791.61205107	-791.28099851
Ru L2 / bis-amide (active catalyst)	-790.36486625	-790.05559642	0.30926983	-790.40307966	-790.09380983
Rh L2 / IPA TS	-1000.94920701	-1000.53699947	0.41220754	-1001.01717911	-1000.60497158
Rh L2 / pro-R TS	-1192.65137592	-1192.18948453	0.46189139	-1192.77434608	-1192.31245469
Rh L2 / pro-S TS	-1192.64945319	-1192.18722527	0.46222792	-1192.77074790	-1192.30851998
Rh L2 / pre-catalyst (cycle starting point)	-1267.99035274	-1267.64660409	0.34374866	-1268.04943402	-1267.70568536
Rh L2 / pre-catalyst N1 deprotonated	-1267.49118516	-1267.16409977	0.32708539	-1267.54862788	-1267.22154249
Rh L2 / pre-catalyst N2 deprotonated	-1267.49114562	-1267.16354675	0.32759887	-1267.54985742	-1267.22225854
Rh L2 / metal hydride	-807.83649514	-807.50055967	0.33593547	-807.87008384	-807.53414837
Rh L2 / bis-amide (active catalyst)	-806.62819920	-806.31476029	0.31343892	-806.65905810	-806.34561918
Acetone	-193.11807988	-193.06935007	0.04872981	-193.16462550	-193.11589570
Acetophenone	-384.81733508	-384.72045550	0.09687959	-384.91763923	-384.82075964
Chloride anion	-460.35379340	-460.37208371	-0.01829031	-460.38137116	-460.39966147
2-propanol	-194.31650198	-194.24327836	0.07322362	-194.35810717	-194.28488355
(R)-1-phenylmethanol	-386.01392901	-385.89450012	0.11942889	-386.11063719	-385.99120830
(S)-1-phenylmethanol	-386.01392860	-385.89449894	0.11942966	-386.11063680	-385.99120714

t-butoxide anion	-233.10289129	-233.01792206	0.08496923	-233.15752627	-233.07255704
t-butanol	-233.62331050	-233.52476937	0.09854112	-233.67123216	-233.57269103

Energies of the species used for the ATH catalytic cycles. Calculated in Orca 5.0.4 at the ωB97M-V/def2-QZVPPD/CPCM(Ethanol) // r^{2}SCAN-3c/CPCM(Ethanol)/353.15 K

Calculated structures of select species

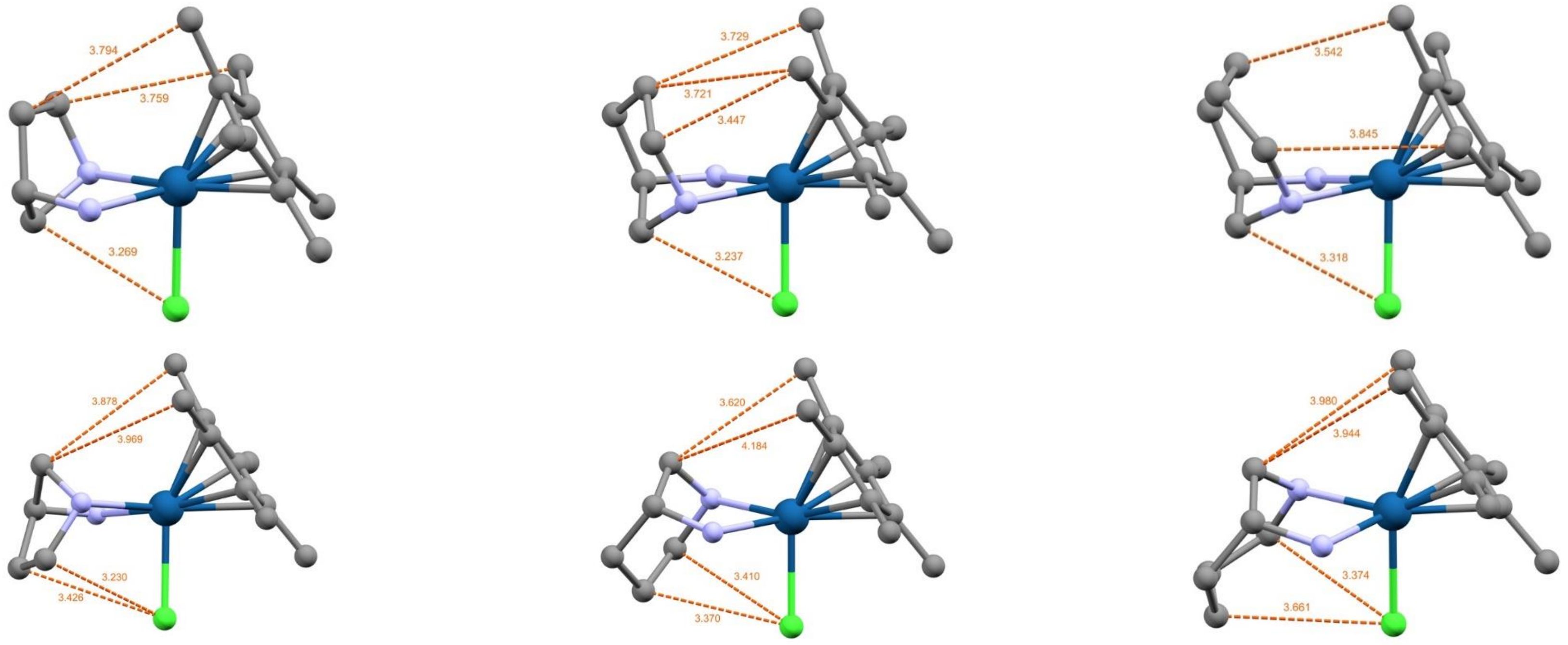

Structures of the calculated diastereomers for the Ir complexes with select distances displayed – from left to right: 5-,6-,7-membered azacycle ligands ; preferred diastereomers are on the top row. Calculated in Orca 5.0.4 at the ωB97M-V/def2-QZVPPD/CPCM(DCM) // r^2SCAN-3c/CPCM(DCM)/298.15 K. Structures generated using Mercury and POV-Ray.

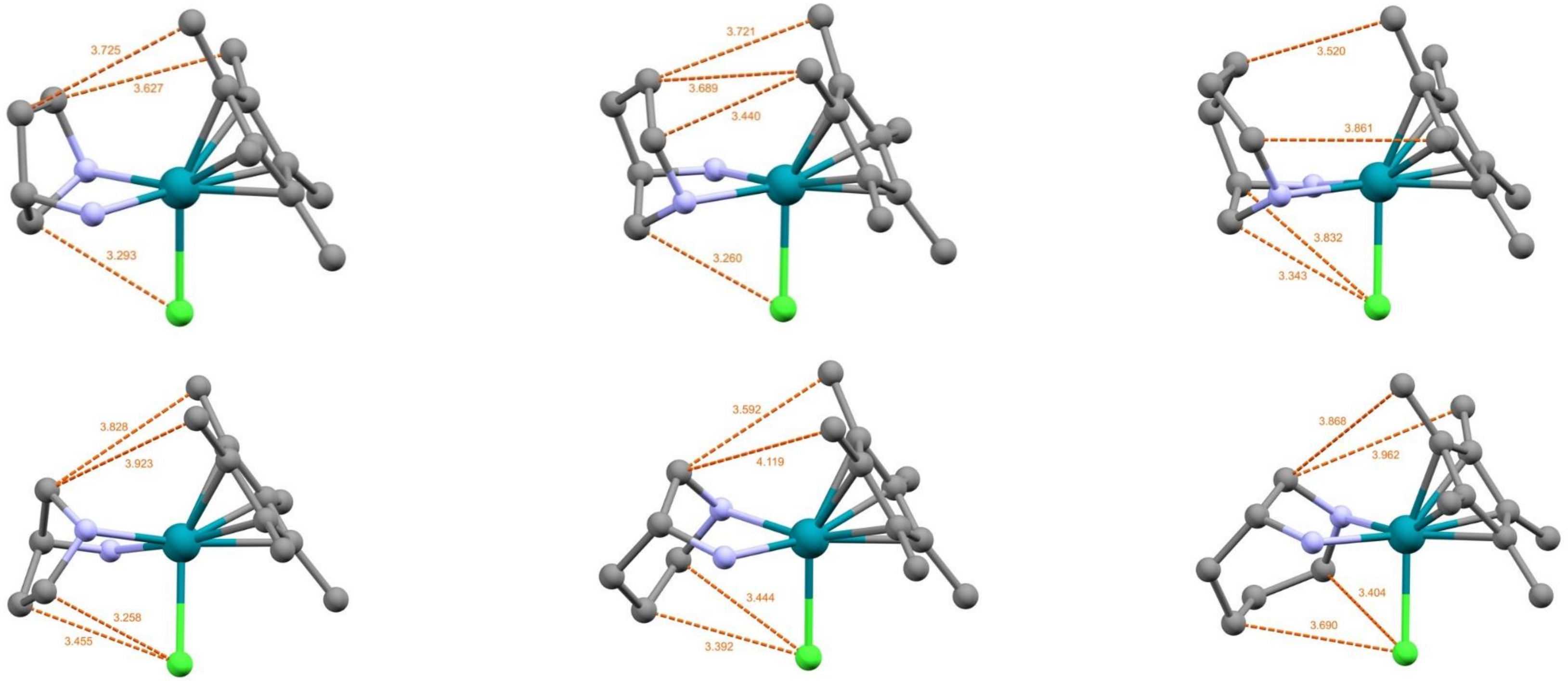

Structures of the calculated diastereomers for the Rh complexes with select distances displayed – from left to right: 5-,6-,7-membered azacycle ligands ; preferred diastereomers are on the top row. Calculated in Orca 5.0.4 at the ωB97M-V/def2-QZVPPD/CPCM(DCM) // r^2SCAN-3c/CPCM(DCM)/298.15 K. Structures generated using Mercury and POV-Ray.

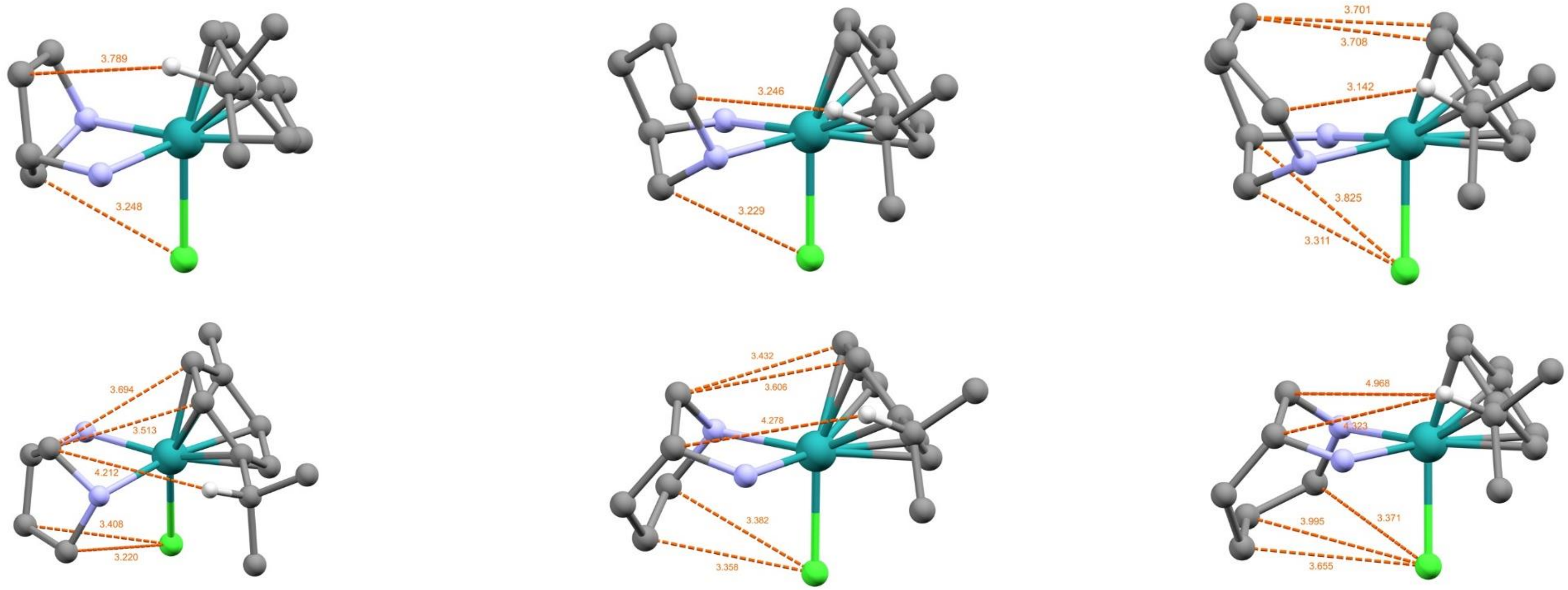

Structures of the calculated diastereomers for the Ru complexes with select distances displayed – from left to right: 5-,6-,7-membered azacycle ligands ; preferred diastereomers are on the top row. Calculated in Orca 5.0.4 at the ωB97M-V/def2-QZVPPD/CPCM(DCM) // r^{2}SCAN-3c/CPCM(DCM)/298.15 K. Structures generated using Mercury and POV-Ray.

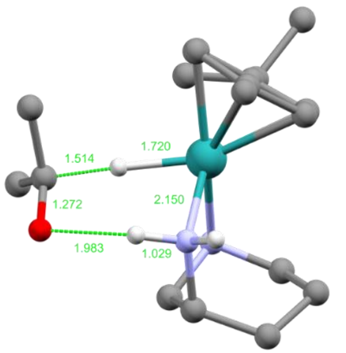

Ru(L2) / 2-propanol transition state
Imaginary mode: -310.03 cm^{-1}

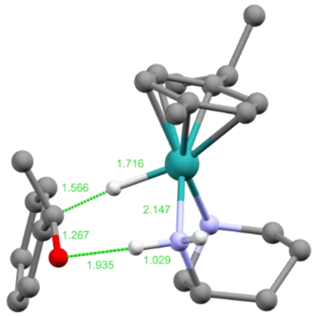

Ru(L2) / acetophenone transition state
(pro-*R* configuration)
Imaginary mode: -324.08 cm^{-1}

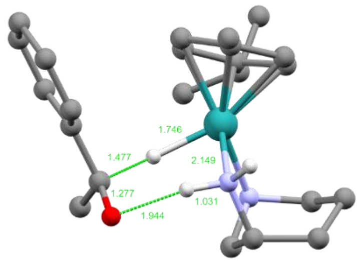

Ru(L2) / acetophenone transition state
(pro-*S* configuration)
Imaginary mode: -247.27 cm^{-1}

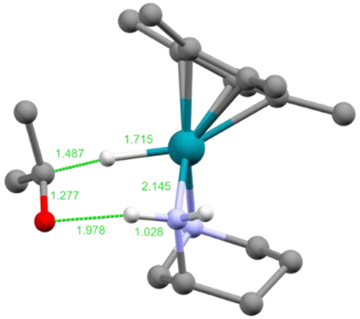

Rh(L2) / 2-propanol transition state
Imaginary mode: -325.40 cm^{-1}

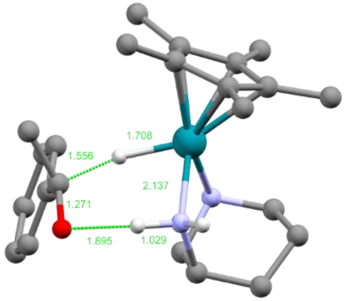

Rh(L2) / acetophenone transition state
(pro-*R* configuration)
Imaginary mode: -392.30 cm^{-1}

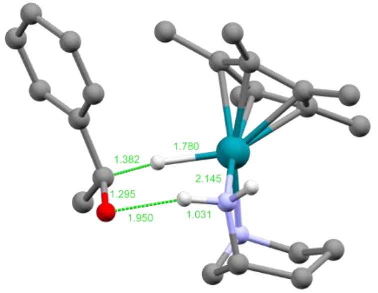

Rh(L2) / acetophenone transition state
(pro-*S* configuration)
Imaginary mode: -177.58 cm^{-1}

Structures of the calculated of the transition state geometries for Ru/Rh(L2) with select distances displayed. Calculated in Orca 5.0.4 at the ωB97M-V/def2-QZVPPD/CPCM(Ethanol) // r^2SCAN-3c/CPCM(Ethanol)/253.15 K. Structures generated using Mercury and POV-Ray.
